# High-throughput identification of heavy metal binding proteins from the byssus of chinese green mussel (*Perna viridis*) by combination of transcriptome and proteome sequencing

**DOI:** 10.1371/journal.pone.0216605

**Published:** 2019-05-09

**Authors:** Xinhui Zhang, Huiwei Huang, Yanbin He, Zhiqiang Ruan, Xinxin You, Wanshun Li, Bo Wen, Zizheng Lu, Bing Liu, Xu Deng, Qiong Shi

**Affiliations:** 1 Shenzhen Key Laboratory of Marine Bioresource and Eco-Environmental Science, College of Life Sciences and Oceanography, Shenzhen University, Shenzhen, China; 2 Shenzhen Key Lab of Marine Genomics, Guangdong Provincial Key Lab of Molecular Breeding in Marine Economic Animals, BGI Academy of Marine Sciences, BGI Marine, BGI, Shenzhen, China; 3 BGI-Shenzhen, BGI, Shenzhen, China; 4 Shenzhen Horus Marine Technology Co. Ltd., Shenzhen, China; 5 Laboratory of Aquatic Bioinformatics, BGI-Zhenjiang Institute of Hydrobiology, BGI Marine, BGI, Zhenjiang, China; Nazarbayev University, KAZAKHSTAN

## Abstract

The Byssus, which is derived from the foot gland of mussels, has been proved to bind heavy metals effectively, but few studies have focused on the molecular mechanisms behind the accumulation of heavy metals by the byssus. In this study, we integrated high-throughput transcriptome and proteome sequencing to construct a comprehensive protein database for the byssus of Chinese green mussel (*Perna viridis*), aiming at providing novel insights into the molecular mechanisms by which the byssus binds to heavy metals. Illumina transcriptome sequencing generated a total of 55,670,668 reads. After filtration, we obtained 53,047,718 clean reads and subjected them to *de novo* assembly using Trinity software. Finally, we annotated 73,264 unigenes and predicted a total of 34,298 protein coding sequences. Moreover, byssal samples were analyzed by proteome sequencing, with the translated protein database from the foot transcriptome as the reference for further prediction of byssal proteins. We eventually determined 187 protein sequences in the byssus, of which 181 proteins are reported for the first time. Interestingly, we observed that many of these byssal proteins are rich in histidine or cysteine residues, which may contribute to the byssal accumulation of heavy metals. Finally, we picked one representative protein, Pvfp-5-1, for recombinant protein synthesis and experimental verification of its efficient binding to cadmium (Cd^2+^) ions.

## Introduction

Next-generation sequencing (NGS) technologies have been employed at a large scale for molecular studies of non-model organisms [1]. They have promoted the development of transcriptome sequencing, which usually presents a complete set of transcripts in a tissue or cell for revealing molecular bases of functional responses at specific developmental stages or to environmental changes [2, 3]. Many molecular changes of an organism upon environmental stress can be interpreted in a comprehensive way through high-throughput transcriptomes [4]. Proteome sequencing by liquid chromatography tandem mass spectrometry (LC-MS/MS) is another effective technique for the high-throughput identification of proteins, and it has proved to be an effective tool to characterize protein structures in model or non-model species [5–7]. In contrast to conventional methods, proteome sequencing allows for the identification of a large number of proteins in one sample.

Many metal ions are essential in organisms for various physiological roles, but they become toxic at high concentrations. Anthropogenic activities and products (such as waste, sewage, and industrial wastewater) release heavy metals into aquatic environments and generate a serious threat to ecosystems [8]. Heavy metal ions are very difficult to remove from aquatic environments by using physical, chemical, or biological methods. However, some organisms have attracted increasing attention due to the effective accumulation of heavy metals in their bodies; they can be used directly or indirectly for decontamination of heavy metals from aquatic environments. For example, certain algae and bacteria can be used for the clean-up of environments contaminated with heavy metals [9, 10]. Mussels have also been extensively applied to environmental monitoring programs [11]. Many Mytilidae mussels have been employed as biomonitors throughout the Indo-Pacific region for assessing chemical and heavy metal pollutants [12, 13]. They are useful due to their widespread distribution and sedentary life style, and they grow enough tissue for studying the accumulation of heavy metals.

Mussels can generate high-performance natural adhesives, which have been applied for surgery, cell culture, immunohistochemistry, sealants, coatings, and anchoring purposes [14, 15]. The mussel byssus has a strong adhesive capacity, which keeps the mussel stably stuck to rocks or growing substrates in strongly flowing waters. The molecular mechanisms of adhesion in mussels have been well studied before [16–18]. We previously reported that the majority of heavy metals accumulate in the byssus, and even after separation from the mussels, the byssus still contains heavy metals [19, 20]. In this study, we tried to reveal the composition of the byssus of the Chinese green mussel (*Perna viridis*), aiming at providing novel insights into the molecular mechanisms of byssal binding to heavy metals. Therefore, we combined transcriptome and proteome sequencing to explore the diversity of byssal proteins in this mussel species. Through this integrative approach, we identified many novel protein sequences that have not been previously reported in any public protein database, and we provide basic data for in-depth studies on novel byssal proteins. Our ultimate goal is to combine our knowledge about the molecular structures and the mechanical features of the byssus and to design byssal-protein-based biomaterials for the removal of heavy metal pollutants from aquatic environments.

## Materials and methods

### Sample collection and total RNA extraction

**Fig 1:**
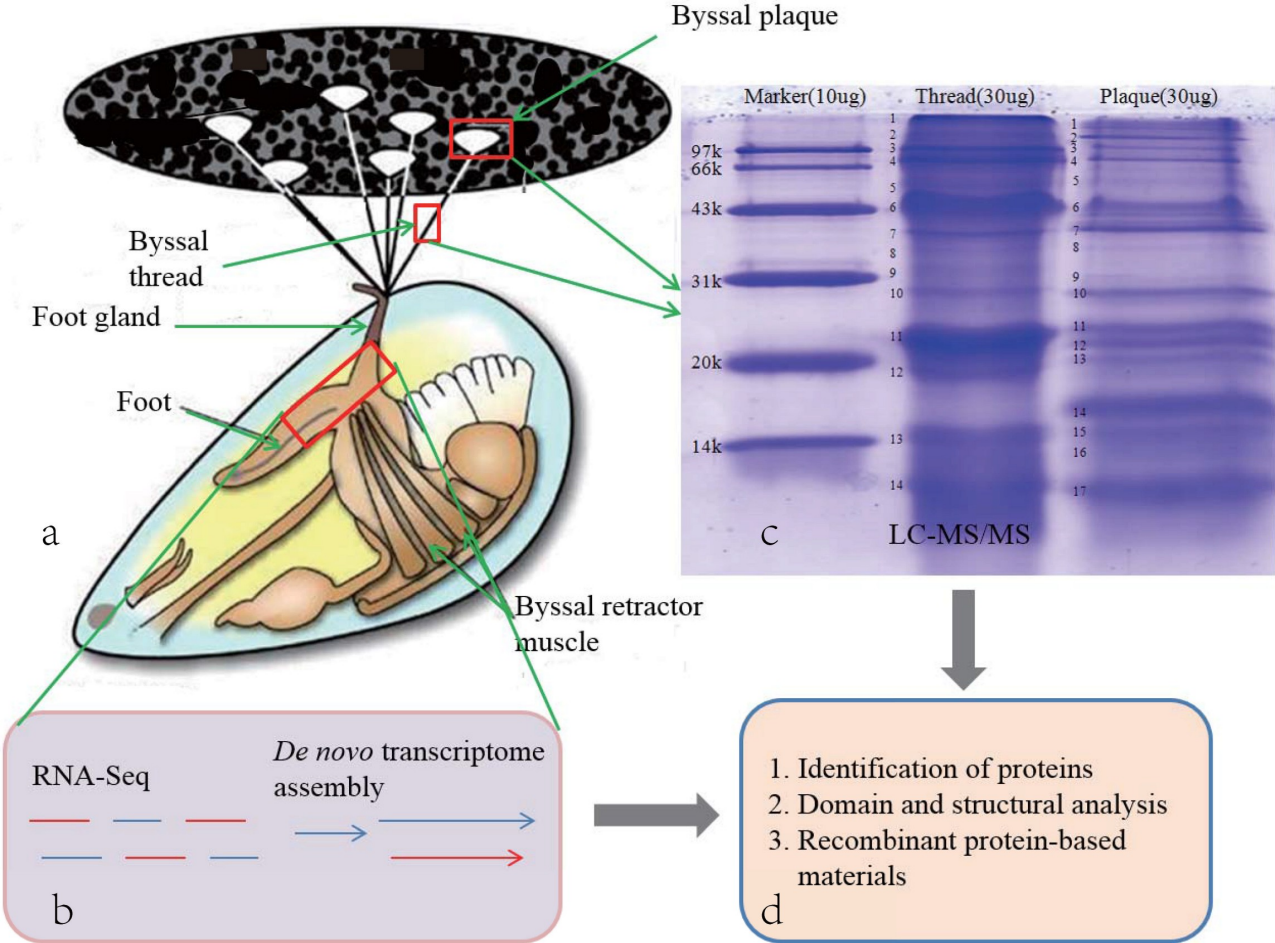
Strategy for integration of transcriptome and proteome sequencing. (**a**) The foot area, byssal threads and byssal plaques (rectangles from bottom to top) were dissected for sequencing. (**b**) Transcriptome sequencing of the foot area was performed for subsequent *de novo* assembly and annotation. (**c**) Thread and plaque proteins were separated by SDS-PAGE before LC-MS/MS analysis. (**d**) The generated transcriptome data were integrated with the proteome sequencing data to identify interesting transcripts and deduce their corresponding protein sequences. Further protein structural analysis, recombinant protein engineering, and biomimetic material processing are examples of potential applications.

Fresh specimens of *P*. *viridis* (30 individuals, shell length 6–8 cm) were collected from a local market in Yantian District, Shenzhen, Guangdong Province, China. The foot areas of 5 mussels (near the foot gland; **Fig 1A**) were collected and snap frozen in liquid nitrogen before storage at −80°C. Total RNA of each sample was extracted using the RNeasy Mini Kit (Qiagen, Hilden, Germany) following the manufacturer’s instructions. After treatment with RNase-Free DNase I (Thermo Fisher Scientific, Waltham, MA, USA) to eliminate genomic DNAs, the extracted mRNAs were reverse transcribed to construct a cDNA library for further transcriptome sequencing.

### Transcriptome sequencing and data analysis

The cDNA library was sequenced using a HiSeq2000 sequencing platform (Illumina, San Diego, CA, USA) with the 90-bp paired-end (PE) sequencing module. We subsequently filtered raw reads to remove adapter sequences and reads with more than 5% of non-sequenced (N) bases or with a quality value below 20. We then employed Trinity software [21] to assemble clean reads to obtain contigs and unigenes. Functions of these unigenes were further predicted on the basis of sequence similarity searches with several public databases, including the NCBI non-redundant protein database (Nr), NCBI non-redundant nucleotide database (Nt), Kyoto Encyclopedia of Genes and Genomes (KEGG), Swiss-Port, and Clusters of orthologous groups of proteins (COG).

We also employed Blast2GO [22] to predict unigenes and obtain gene ontology (GO) annotation for each unigene. Subsequently, we performed GO functional classification of these unigenes using WEGO [23]. KEGG annotation was also applied to obtain pathway annotation for these unigenes. We searched unigene sequences against the public databases using BLASTX (E-value ≤ 1.0e^-5^), with a priority order of Nr, Swiss-Port, KEGG, and COG. The alignment results were subsequently used to determine coding sequences of the unigenes and translate them into amino acid sequences. If unigenes had no hit in any known protein database, their coding sequences were predicted using ESTScan [24], and also translated into the corresponding protein sequences.

### Protein fractionation and mass-spectrometry (MS) analysis

Twenty of the collected mussels were cultured in a glass tank at 26–28°C, where they generated threads and plaques overnight. Threads (0.5 g; pooled from 10 mussels) and plaques (0.3 g; pooled from 10 mussels) were harvested (**Fig 1A**) for further grinding in liquid nitrogen. After the addition of acetic acid (1 ml, 5%) and treatment by ultrasound for 3 min, the protein lysates were centrifuged at 19,160 ×*g* for 15 min at 4°C to remove debris. After the addition of 100 μl of L3 Buffer (7 M urea, 2 M thiourea, 50 mM Tris-HCl, pH 8.0) to each lysate, the supernatants were used as plaque (1.02 μg/μl) and thread (5.91 μg/μl) protein extracts, respectively.

The obtained protein solutions were subjected to SDS-PAGE (**Fig 1C**) followed by in-gel digestion with trypsin [25] in 10 μl of 50 mM NH_4_HCO_3_ for 12 h at 37°C. Subsequently the pooled mixtures of peptides were fractionated into 10 portions using SCX chromatography (GE, Boston, MA, USA). The fractionated peptides were further separated by LC-20AD (Shimadzu, Kyoto, Japan) high-pH reverse-phase chromatography and analyzed by LTQ-Orbitrap Velos (Thermo Fisher Scientific) [26].

The acquired MS data were converted to MGF files by Proteome Discoverer 1.4 (Thermo Fisher Scientific), and then the exported MGF files were searched using Mascot (v2.3.02; MatrixScience, London, UK) against the byssal-transcriptome-annotated database. Mascot parameters were set as follows. Trypsin was selected as the specific enzyme with a maximum of 1 missed cleavage permitted per peptide; fixed modifications of carbamidomethyl (C); variable modifications consisting of oxidation (M), deamidatioin (N, Q) and Gln->pyro-Glu (N-term Q); peptide charge, 2+, 3+, and 4+; 20 ppm of peptide mass tolerance; 0.05 Da of fragment mass tolerance. The automatic Mascot decoy database search was performed, and the Mascot results were processed by IQuant [27]. MascorPercolator was utilized to re-score the peptide spectrum matches (PSMs) [28, 29]. The identified peptide sequences were subsequently assembled into a set of confident proteins using the Occam’s razor approach implemented in IQuant. Finally, the false discovery rate (FDR) was set at 1%, at both the PSM and the protein levels [30].

### Reverse-transcription PCR (RT-PCR)

Total RNA was extracted as described above. Reverse transcription of cDNA was subsequently performed with 2 μg of DNase-treated total RNA using the M-MuLV First Strand cDNA Synthesis Kit (Sangon, Shanghai, China). We randomly selected 6 byssal protein coding genes and designed primer pairs using Primer Premier 5.0 (**S1 Table**) for PCR validation. The primary RT-PCR reactions were carried out in a volume of 50 μl, containing 0.5 μl of rTaq DNA Polymerase (Toyobo, Osaka, Japan), 0.5 μl of cDNA (1,000 ng), 1×PCR reaction buffer, 0.2 μM of forward and reverse primers, and 200 μM of each dNTP. DNA amplification on an ABI 9700 thermal cycler (Thermo Fisher Scientific) was performed with the following cycling conditions: initial denaturation at 94°C for 5 min; then 35 cycles of 94°C for 30 sec, 55°C for 30 sec and 72°C for 1 min; final extension at 72°C for 10 min. All PCR amplicons were analyzed by 1.5% agarose gel electrophoresis for further sequencing validation.

### Pvfp-5-1: Cloning, protein expression and purification

The protein sequence of Pvfp-5-1, a byssal protein, was obtained from the LC-MS/MS analysis. Molecular cloning and standard recombinant DNA techniques were applied to clone the Pvfp-5-1 gene into *E*. *coli*. Codon adaptation of the amino acid sequences of Pvfp-5-1 was carried out by online codon optimization software of the Codon Adaptation Tool (JACT) [31]. Forward and reverse primers containing *BamHI* and *XhoI* restriction sites (5’-GGATCCTACGACTACCGTGA-3’ and 5’-CTCGAGGTAGTATTTACCAG-3) were designed, respectively, using the modified Pvfp-5-1 nucleotide sequence (**S2 Table**).

The Pvfp-5-1 plasmid was mixed with competent *E*. *coli* cells that were subsequently cultured on LB supplemented with 100 μg/ml of ampicillin overnight at 37°C. Sequencing was performed to identify Pvfp-5-1-positive colonies. After the colony confirmation, we used a Prime Prep Plasmid DNA Isolation Kit (GeNet Bio, Cheonan, South Korea) to extract the Pvfp-5-1 and pET-32a vectors and digested them with *BamHI* and *XhoI* at 37°C for 4 h. The Pvfp-5-1 construct was separated on a 1% agarose gel, purified with a Prime Prep Gel Purification Kit (GeNet Bio), and then ligated into the multiple cloning site (MCS) of the T7lac promoter expression plasmid pET-32a with T4 DNA ligase (Thermo Fisher Scientific). To confirm the successful cloning of the full length of Pvfp-5-1 into the pET-32a vector, we extracted and sequenced these recombinant plasmids. Only the validated pET-32a-Pvfp-5-1 plasmid was transformed into *E*. *coli* BL21 (DE3) to obtain purified cells for expression of the Pvfp-5-1 gene. The cells were cultured in 50 ml of liquid LB, incubated in a shaker at 37°C for 12–16 h, and then inoculated in 200 ml of liquid LB at a ratio of 1: 100. After incubation at 37°C until an OD of 0.5~0.7 was reached, IPTG was added to the cell culture at a final concentration of 1 mM, and continuous shaking was performed for 4 more hours. Subsequent centrifugation at 1,532 ×*g* for 15 minutes (4°C) was carried out, and the cells were collected and stored at −20°C until further use.

Moreover, we collected 200 μl of the upper bacterial supernatant for SDS-PAGE analysis. We added 25 μl of distilled water and 25 μl of 2× protein loading buffer to each sample before boiling at 100°C for 10 minutes. After a short centrifugation, the protein products were separated by standard SDS-PAGE [32].

### Enrichment experiment of Cd^2+^ by the recombinant Pvfp-5-1 protein

Cadmium solutions (50 and 100 μg/l) were prepared by dissolving cadmium chloride (CdCl_2_) in double distillated H_2_O (ddH_2_O). A CdCl_2_ concentration of 50 μg/ml (experimental groups 5A, 5B, and 5C) or 100 μg/ml (groups 10A, 10B, and 10C) was used. In each experiment group, 100 μl, 300 μl, or 500 μl of recombinant Pvfp-5-1 solution was added to 3 ml of CdCl_2_ solution. In the corresponding control groups, the same volume of pET-32a was added to the CdCl_2_ solution (**Table 1**). Cd^2+^ quantification was realized using inductively coupled plasma mass spectrometry (ICP-MS) with a NexION 300X (PerkinElmer, Boston, MA, USA) for the calculations, following the manufacturer’s instructions. Each experiment was repeated three times. We used the Student’s *t* test for statistical analysis, where *P* < 0.05 was considered statistically significant.

**Table 1:** Design of the enrichment experiment.

Volume (μl)	Cd^2+^ 50 μg/L		Cd^2+^ 100 μg/L	
	Experiment	Control	Experiment	Control
100	Pvfp5-1 (5A)	pET-32a	Pvfp5-1 (10A)	pET-32a
300	Pvfp5-1 (5B)	pET-32a	Pvfp5-1 (10B)	pET-32a
500	Pvfp5-1 (5C)	pET-32a	Pvfp5-1 (10C)	pET-32a

## Results

### Data summary for the high-throughput transcriptome sequencing and *de novo* assembly

We sequenced a foot transcriptome of *P*. *viridis* (**Fig 1A**) and generated a total of 55,670,668 raw reads. After filtration, we subjected the 53,047,718 clean reads to subsequent *de novo* assembly using Trinity software. Finally, we obtained 73,571 unigenes. Lengths of the assembled unigenes ranged from 200 bp to 14,157 bp, with an average of 599 bp and an N50 of 794 bp (**S3 Table**).

### Functional annotation of the predicted unigenes

BLASTX alignment (E-value ≤ 1.0e^-5^) was performed for these unigenes to search public protein databases. The results (**S4 Table**) indicate that within the total 73,571 unigenes, 29,973 were annotated against the Nr, 18,615 against the KEGG, 9,466 against the GO, 22,988 against the Swiss-Prot, and 6,721 against the Nt.

Based on the COG annotation, 8,834 unigenes were predicted and classified into 25 functional categories (**S1 Fig**). “General function prediction only” was the most popular group (19.72%), followed by “Replication, recombination and repair” (9.10%) and “Translation, ribosomal structure and biogenesis” (7.45%). For the GO annotation, 9,466 unigenes were assigned GO terms and categorized into 51 subcategories (**S2 Fig**) belonging to 3 main categories.

“Binding and catalytic activity” was the largest group in the category of molecular function. In the category of biological processes, “cellular process” was obviously the most dominant; however, in the cellular component, “cell part” was the largest representative. According to the KEGG annotation results, 18,615 unigenes were annotated and assigned to 241 KEGG pathways. The most common classifications include “metabolic pathway” (2,295 unigenes), “focal adhesion” (955 unigenes), “pathway in cancer” (852 unigenes), and “regulation of actin cytoskeleton” (838 unigenes). For the KEGG annotation, we observed that 955 unigenes were annotated in the focal adhesion pathway, which is related to the adhesive function of the byssus. Jointly, the annotations of GO terms and KEGG pathways provide a useful resource for further identification of specific cellular structures, pathways, processes, and protein functions in the Chinese green mussel.

In summary, we employed BLAST searches against the important public databases (Nr, Swissi-Prot, KEGG, GO, COG, and Nt) to show that a total of 31,710 assembled unigenes were annotated to known biological functions (see more details in **S4 Table**).

### Byssal proteins revealed by the LC-MS/MS analysis

Proteomic analysis of the *P*. *viridis* byssus has previously been reported, but few byssal proteins were identified [33, 34]. In order to uncover the complexity of the byssus, we determined the byssal proteins on a more sensitive Prominence Nano-HPLC system coupled with Q-Exactive. After separation of the total byssal proteins using SDS-PAGE, we obtained 14 (named as S1–S14) and 17 (named as P1–P17) protein bands from the byssal thread and plaque, respectively (**Fig 1C**).

The total 31 protein bands were cut out individually and digested by trypsin for subsequent LC-MS/MS determination. The generated data were analyzed by Mascot software (v2.3.02) with the byssus-transcriptome-based protein database (i.e., translated from the transcriptome-based transcripts) as the reference for protein prediction. A total of 1,031 unique peptides were identified, and 187 protein sequences were predicted (**S5 Table**), in which 130 proteins matched with multiple peptides and 57 proteins matched with only one peptide. Interestingly, the numbers of peptides and proteins from the byssal thread are higher than those from the byssal plaque (**S5, S6**and **S7 Tables**).

Detailed information about the identified foot proteins was listed in **S6**and **S7 Tables**, including identified peptide sequences, unique peptide numbers, and protein coverage. The spectra of all unique peptides labeled with PDV software (https://github.com/wenbostar/PDV) are provide in **S3 Fig**; the precursor m/z, mass error, and expect value for each spectrum are presented in **S8 Table**.

We subsequently used the CD-HIT program [35] to remove redundant sequences, and we finally identified 187 protein sequences (**S9 Table**). Among these predicted proteins, 181 proteins showed only partial sequence similarity to known proteins, implying that most of these byssal proteins are novel. Many byssal proteins were only partially resolved in our present work, possibly due to their low abundance.

**Fig 2:**
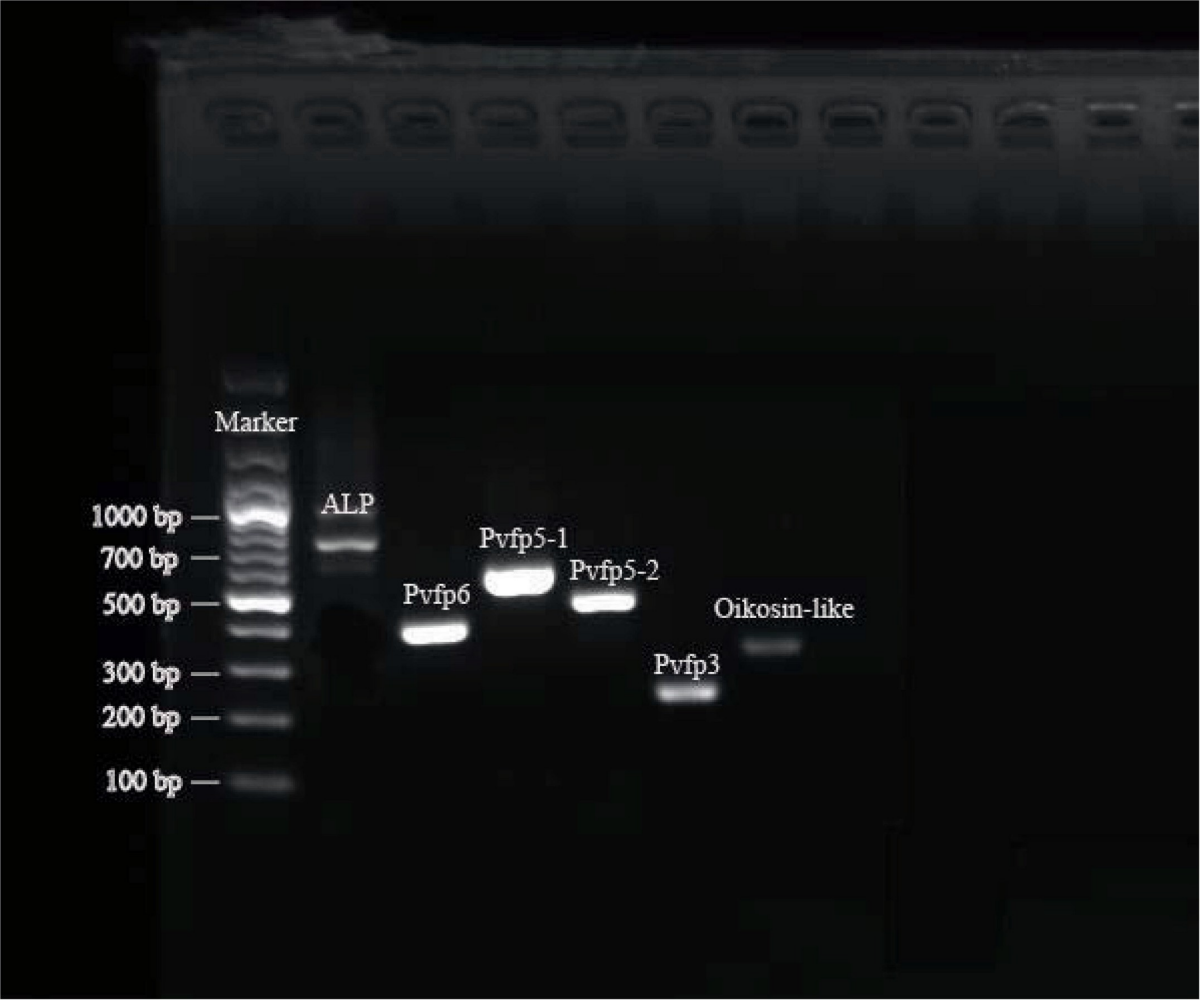
Validation of byssal proteins by RT-PCR with further confirmation by Sanger sequencing.

Among the identified 187 byssal protein sequences, 113 sequences were assigned to 79 KEGG pathways (**S10 Table**), in which “Focal adhesion” was the most common group (15.9%). To validate the accuracy of these predicted byssal protein sequences, we randomly picked 6 sequences for validation by RT-PCR (**Fig 2**) with subsequent Sanger sequencing.

### Content and distribution of histidine and cysteine residues in byssal proteins

Histidine (His, H) and cysteine (Cys, C) residues play important roles in heavy metal binding peptides and/or proteins [36–38]. In particular, the metal binding properties make cysteine an important component of many proteins and a key catalytic component of enzymes [39]. As is well known, cysteine-rich metallothioneins (MTs) are important metal binding proteins, in which the Cys-Cys, Cys-X-X-Cys, and Cys-X-Cys motifs (X denotes any amino acid) are remarkable [36, 40, 41].

**Fig 3:**
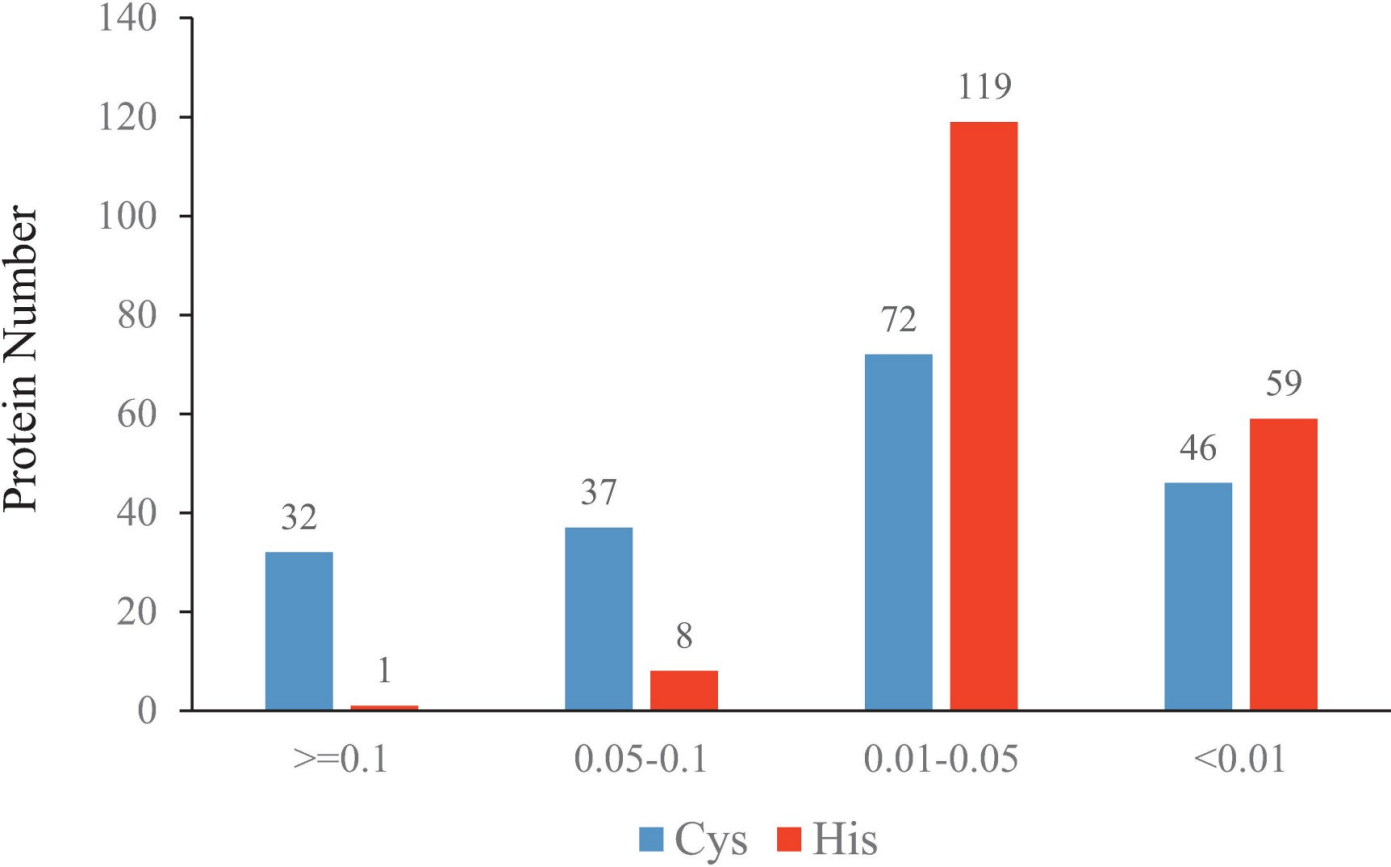
**Content and distribution of histidine (H) and cysteine (C) residues in the byssal protein sequences of *P*. *viridis*.** The x-axis represents the content of histidine (red) and cysteine (blue) in each protein. The y-axis represents the number of proteins.

In our present work, through protein structural analysis, we observed that several byssal proteins are rich in histidine residues or cysteine residues or contain a cysteine-rich domain. A cysteine content of >10% and 5%–10% was found in 32 and 37 byssal proteins, respectively; the histidine content was mainly in the range of 1% to 5%, and one protein contained more than 10% (see more details in **Fig 3**). In the byssal proteins of our interest (i.e., Pvfp-2, -3, -5-1, -5-2, and -6), cysteine residues or Cys-X-Cys motifs are abundant (**Table 2**).

**Table 2:** Identified byssal proteins from the Chinese green mussel.

Foot proteins	Search conditions	Identified foot protein sequences and their characteristics
**Pvfp-1** (partial)	Homology sequences	MARNMNILTLFAVLLGSASAVYHPPSWTAWIAPKPWTAWKVP PPAWTAWKAHPPAWTAWKATPKPWTAWKA
**Pvfp-2** (partial)	Homology sequences	(CXXXPCXXXGXCXXXXXXXXYKCXCXXGYTGXXCX)n High content of Y and C
**Pvfp-3**	Homology sequences	MKCTLFSIWVVVFAISGMNYVDAQLTCFPTIDCGFNIDGCQS FCRDRNCSPYGSECRGNNQCCCFGCTYG Cys content > 15%
**Pvfp-4** (partial)	LC-MS/MS	**HETFIAR**MW = 34kDa; High content of Y and H
**Pvfp-5-1**	Homology sequences	MLKFVVLAVVLCAFYVQAYDYRDPCKPRPCVNGGTCCRKGSS YTCKCRYGYYGKNCQYNSCSPSPCKNGGTCKCLGGSKFRCYC KKGYKGKYCQYGPCYTNPCLNGGTCAYMYGLPFYKCSCVPGY YGKKCQIKRYYKDRCGGCLNGGNCICNKYGKYFCKCKSGYSGKRCSGKYY C content > 15%; Y content 14.2
**Pvfp-5-2** (byssal thread)	Homology sequences	MLKLVVLIIVYVCYVQARDYYLNPCLPNPCRYGGTCK**SIGLF** **GYK**CFCTNGYKGKNCQFNACTPNPCLNGGTCALIYGPPFYQC SCPYGYYGTKCEFKRHYYDRCGGCLNGGLCISDSYGKYVCRC KPGYYGKRCIDPYY MW = 13.6kDa; C content 13%; Y content 14.3; 7% peptide coverage
**Pvfp-6**(byssal plaque)	Homology sequencesLC-MS/MS	MISAVCIYFFLVGQIQAGVYIPYEKPGQCPVTR**GITPCVCIP** **ENFECRFDSNCPGAMK**CCDFGCGCNKRCPPVPSPLQCYYNGQ YYPIGAHFPSVDGCNTCYCNDDGTVMCTLKACGYGYK MW = 11kDa; C content >14%; 20.6% peptide coverage

Note: Sequences of the identified byssal proteins were searched by BLAST homology from the foot transcriptome database. Underlined sequences are signal peptides. Bold area are mass spectrum-matched peptide sequences. Prediction of signal peptides was performed with SignalP 4.0 software [42]. MW, molecular weight; X, any amino acid other than cysteine.

### Foot proteins of *P*. *viridis*

**Fig 4:**
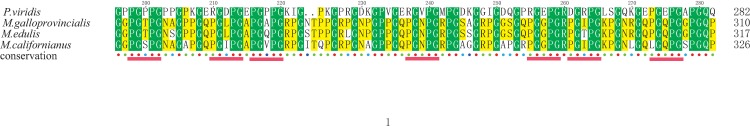
Comparison of partial preCol-P sequences between *P*. *viridis* and *Mytilus* species. Red underlined sequences are XGXPG repeats.

Using known foot protein sequences from other mussels (such as Mefp1–Mefp6 from *Mytilus edulis*; downloaded from the NCBI database) as the queries to perform BLAST homology searches against our newly established transcriptome database and byssal protein database, we identified 7 foot protein sequences (named as Pvfp-1, -2, - 3, -4, -5-1, -5-2, and -6 respectively; **Tables 2 and 3**) in *P*. *viridis*. Interestingly, Unigene22875_2A (**Table 3**) is similar to Mcfp-4 (from *Mytilus californianus*); hence, we renamed it Pvfp-4 (although the sequence is only partially available; **Fig 4**). Despite that only 2 foot protein sequences have been confirmed (Pvfp-4 and -6) in the public protein databases, we should pay attention to the low sequence homology between our predicted Pvfps and previously reported foot proteins from other mussels. The significant species differences may be due to various environmental conditions, such as water temperature, salinity, water flow, and microbial influences [33, 43].

**Table 3:** Byssal proteins identified and annotated from the transcriptome and proteome of *P*. *viridis*.

Unigene ID	Amino acid length	Identity (%)	E-value	Known/novel	Database	Description
Unigene24586_2A	71	100	2e-34	Known	T	Pvfp-1
Unigene26149_2A	348	98	0.0	Known	T	Pvfp-2
Unigene22855_2A	70	91	1e-45	Known	T	Pvfp-3
Unigene22875_2A	281	23	1e-7	Novel	T and P	Pvfp-4
Unigene23062_2A	176	99	2e-118	Known	T	Pvfp-5-1
Unigene24321_2A	140	100	2e-98	Known	T	Pvfp-5-2
Unigene25134_2A	121	98	1e-84	Known	T and P	Pvfp-6
CL121.Contig1_2A	619	99	0.0	Known	T and P	Precollagen NG
Unigene25995_2A	457	95	0.0	Known	T and P	Precollagen D
Unigene26029_2A	561	41	8e-103	Novel	T and P	Precollagen P
Unigene23721_2A	330	100	0.0	Known	T and P	Tyrosinase 1
Unigene24919_2A	284	99	0.0	Known	T	Tyrosinase 2
Unigene23727_2A	340	94	0.0	Known	T and P	Tyrosinase 3
Unigene10448_2A	279	100	0.0	Known	T	Tyrosinase 4
Unigene25716_2A	353	100	0.0	Known	T and P	Tyrosinase 5
Unigene24116_2A	236	41	9e-42	Novel	T and P	Antistasin-like protein
Unigene23933_2A	289	45	1e-66	Novel	T and P	Serine protease inhibitor
Unigene24349_2A	116	42	2e-20	Novel	T and P	Oikosin-like protein
Unigene24173_2A	655	100	0.0	Known	T	Heat shock protein
Unigene62001_2A	526	88	0.0	Novel	T and P	Pernin precusor
Unigene23611_2A	266	54	7e-79	Novel	T and P	Proximal thread matrix protein

Note: Known, existing in the Nr database; Novel, identified by our present work; T, foot transcriptome of *P*. *viridis*; P, byssal proteome of *P*. *viridis*.

### Other byssus proteins: Precollagen and tyrosinase in *P*. *viridis*

The byssus contains 3 peculiar collagen proteins, named preCol-NG, preCol-D, and preCol-P [44]. It was reported that preCol-D localizes to the stiff distal portion, preCol-P is present in the proximal portion, while preCol-NG is evenly distributed [45]. By homology searches against our proteome database, we identified 3 preCols (**Table 3**), among which preCol-P is novel. Homology was predominantly found in the conserved central domain with several pentapeptide repeat sequences, XGXPG, where X denotes a glycine or hydrophobic residue (red underlined in **Fig 4**); the glycine residues of the mature proteins are highly conserved between *P*. *viridis* and *Mytilus* species [44, 46]. Interestingly, these identified collagen proteins exhibited subtle but substantial species-specific modifications, compared with those from other mussels.

Tyrosinase, a copper-containing enzyme [47], can convert tyrosine into adhesive DOPA residues [48]. It has been recognized as a key component of byssal adhesion proteins [49]. By BLASTX homology searches against our transcriptome and proteome databases, we identified 5 tyrosinases (**Table 3**) from the transcriptome and proteome data. Homologous sequences of these tyrosinases are largely localized in the conserved active sites (comprising 7 histidine residues), which contain 2 copper binding sites, Cu(A) and Cu(B) [33, 50, 51]. Interestingly, tyrosinases have been reported to bind copper directly, and the Cu(A) and Cu(B) sites are both required to bind copper for catalytic activity [51].

### Accumulation of Cd^2+^ by the recombinant Pvfp-5-1 protein

**Fig 5:**
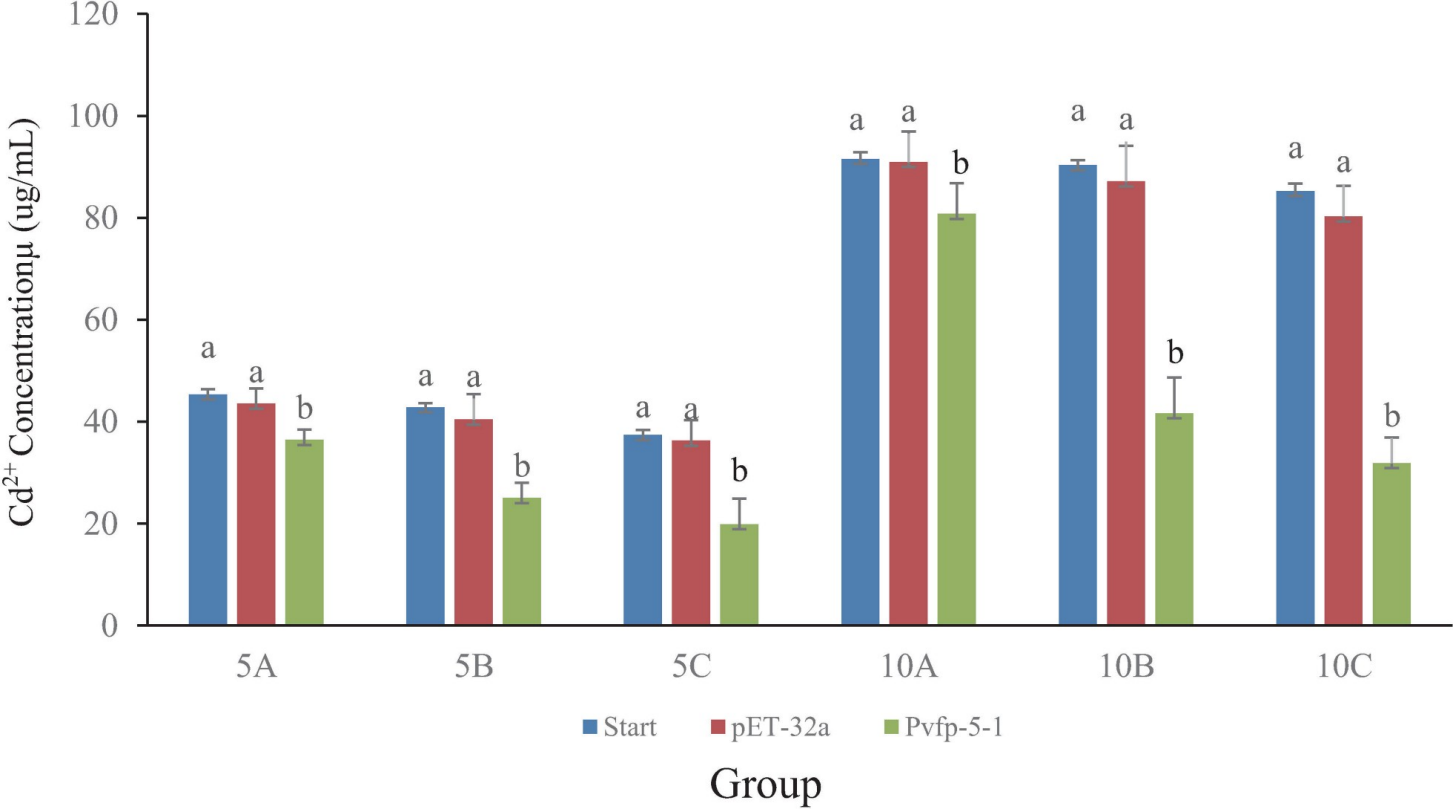
Accumulation of Cd^2+^ by the recombinant Pvpf-5-1 protein. Blue bars represent initial Cd^2+^ concentration, and red or green bars indicate the Cd^2+^ concentrations after addition of the empty pET-32a vector or Pvfp-5-1, respectively. See more details about the groups in **Table 1**.

Our previous studies demonstrated that the byssus can bind heavy metals effectively [20]. In order to examine the heavy metal enrichment ability of byssal proteins, we employed recombinant Pvfp-5-1 (159 mg/l) to study its binding to Cd^2+^. Our results (**Fig 5**) show that the Cd^2+^ concentrations decreased significantly (*P < 0*.*05*) after addition of the purified recombinant Pvfp-5-1 protein to the initial solution. With increasing Pvfp-5-1 concentrations, the final Cd^2+^ concentration decreased. In summary, these data obviously proved the enrichment ability of our recombinant Pvfp-5-1 for heavy metals.

## Discussion

The mussel byssus is composed of many byssal proteins, which present differences in function and biological activity. Several byssal proteins have been identified before, including foot proteins, precollagens, tyrosinases, and proximal thread matrix proteins [37, 46, 52, 53]. It was reported that different byssal proteins, with differential biological functions, make the byssus a valuable resource. For example, natural foot proteins from various *Mytiliu* species have been used as a resource for underwater coatings and adhesives [33, 43, 54]. Interestingly, foot proteins (Fp-1–Fp-6) that presumably act as adhesives can also bind heavy metals [53, 55]. Hence, in the future, we may be able to design novel byssal-protein-based biomaterials to remove heavy metal pollution from aquatic environments. This is our main drive to examine the diversity of the byssal proteins in *P*. *viridis*, i.e., to deal with heavy metal pollution and radioactive waste from local factories.

Proteome sequencing is an efficient and widely used technique for identification of functional proteins. In this research, we combined proteome sequencing with transcriptome sequencing to construct a comprehensive library of *P*. *viridis* byssal proteins. Thousands of peptide fragments and 187 proteins were identified by LC-MS/MS. Six proteins had been reported before, and 181 are novel.

**Fig 6:**
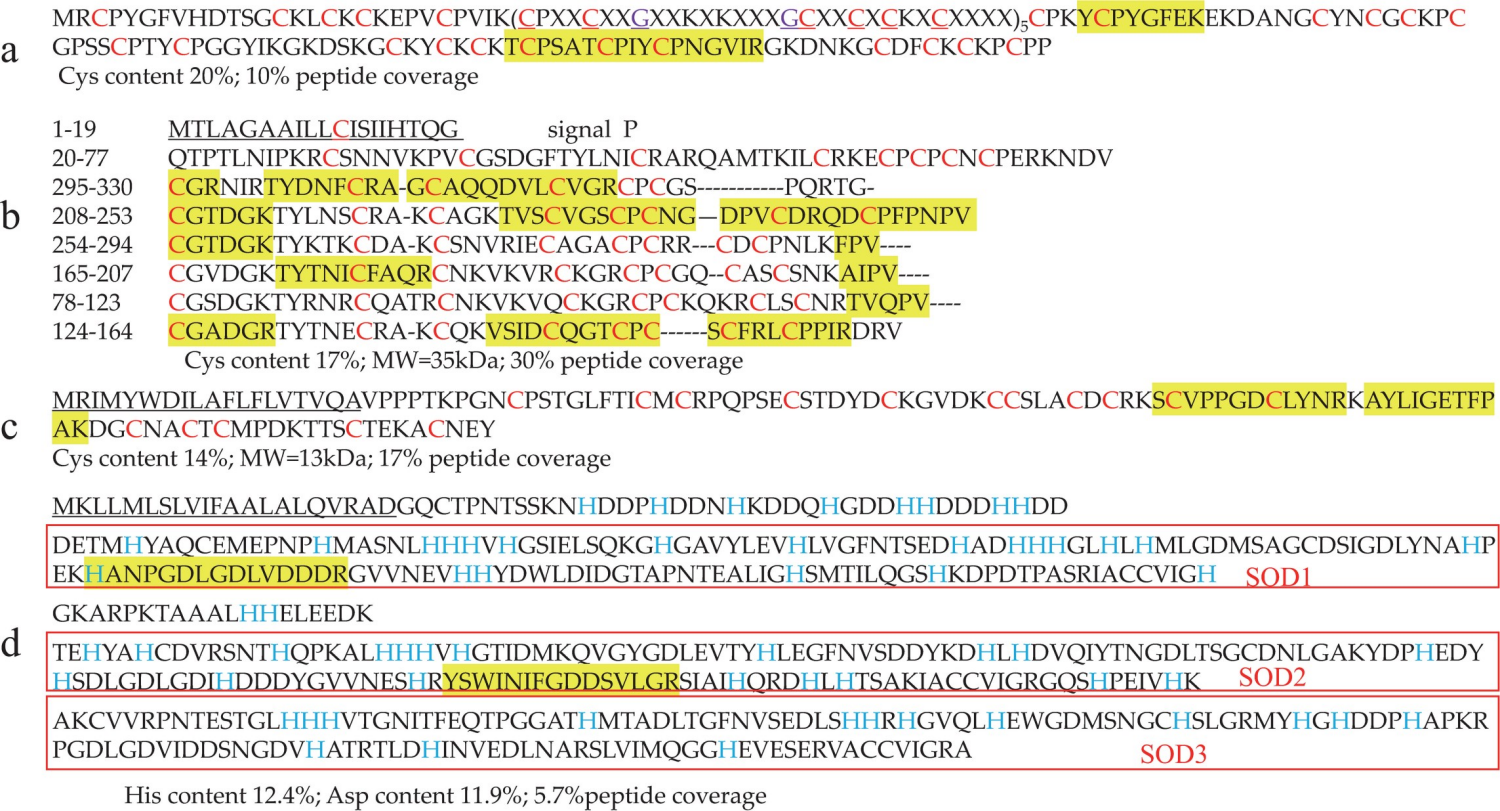
Sequence comparisons of several important byssal proteins. (**a**) antistasin-like protein (ALP). (**b**) SPI-like protein, which contains 6 repeated regions. (**c**) Oikosin-like protein; (**d**) Pernin precursor protein, which contains 3 repeated regions (Cu-Zn SODs in the red boxes). Note that the underlined regions are signal sequences. The cysteine (Cys, C) and Histidine (His, H) residues are highlighted in red and blue, respectively. Yellow areas are the identified peptides by LC-MS/MS.

Metal ions are essential for organisms, but excessive metal ions produce toxic effects. In the face of heavy metal stress, organisms protect themselves by various defense systems, such as synthesis of metal binding proteins or peptides. Histidine and cysteine residues play important roles in heavy metal binding proteins or peptides [38, 56]. In this study, we analyzed the content of cysteine and histidine in byssal proteins, and we observed that several novel byssal proteins are rich in histidine residues or cysteine residue or contain a cysteine-rich domain. For example, Antistasin-like protein (ALP, Unigene24116_2A; **Fig 6A**) is a novel protein in the byssus of *P*. *viridis*, containing internal repeats of a 30-aa sequence with a highly conserved pattern of 6 cysteine (Cys) and 2 glycine (Gly) residues; however, no similar sequences have been identified in other mussels. Over 20% of amino acids in the mature sequence of ALP are cysteine residues, with Cys-X-Cys and Cys-X-X-Cys motifs similar to MTs, indicating that this new protein may be able to bind metals.

Two more novel protein sequences (Unigene23933_2A and Unigene24349_2A; **Table 3**), with molecular weights of 35 kDa (30% peptide coverage) and 13 kDa (17% peptide coverage), respectively, have remarkably high contents of cysteine residues and homology with serine protease inhibitor like (SPI-like) protein and Oikosin-like protein, respectively. The mature peptide sequence of SPI-like protein contains 6 kazal domains of duplication (6 highly conserved cysteine residues, **Fig 6B**). The equence of Oikosin-like protein (Unigene62001_2A) is rich in aspartic acid (11.9%) and histidine (12.4%) residues. It comprises 3 active Cu-Zn superoxide dismutase (SOD) domains of obvious sequence duplication (**Fig 6C**).

Aspartic acid and histidine are known to participate in the binding of many metal cations [57]. The pernin precursor (Unigene62001_2A) has a high histidine content and contains 3 Cu-Zn SOD domains (**Fig 6D**), which might explain its remarkable metal binding capacity. Interestingly, our previous studies have confirmed that, under Cd stress conditions, expression of these byssal protein coding genes (including ALP, Pvfp-1, Pvfp-5-1, Pvfp-5-2, and Pvfp-6) are upregulated [20].

Mussel foot proteins have been applied in underwater experiments and for medicinal purposes. However, the process to extract byssal proteins from the mussel byssus is labor-intensive and inefficient, and approximately 10,000 mussels are required for isolation 1 mg of adhesive proteins [58]. *E*. *coli* can effectively be used for the expression of adhesive proteins, and the microscale assay showed purified recombinant Mgfp-5 has significant adhesive activity [59]. However, not all the foot proteins can be expressed by *E*. *coli*. For example, the recombinant Fp-1 protein has to be decoded in a yeast expression system [60, 61]. The failure in *E*. *coli* system may be due to the highly biased amino acid composition, the long amino acid sequence, or the different codon usage preference between the mussel and *E*. *coli* [62]. In this study, hence, we cloned and expressed recombinant Pvfp-5-1 with sequence modifications, and we confirmed that the newly recombinant Pvfp-5-1 has the capacity to bind Cd^2+^ ions. Our results suggest that the recombinant Pvfp-5-1 could be developed into a commercial product for the removal of heavy metals and/or radioactive waste from aquatic environments.

### Conclusions

In this study, we performed a combination of transcriptome and proteome sequencing to investigate protein components in the foot and byssus (threads and plaques) of the Chinese green mussel. By BLAST homology searches of known sequences from other mussel species against our generated transcriptome and proteome databases, we could rapidly predict and identify a collection of protein sequences in a high-throughput way. Since the mussel byssus has been proved to accumulate heavy metals effectively, we chose several byssal proteins that are rich in cysteine and/or tyrosine residues for structural analysis. Metal binding experiments were further performed to prove the Cd^2+^ binding ability of recombinant Pvfp-5-1. In summary, we have established a valuable resource for the identification of more important proteins, engineering of more recombinant proteins, and development and processing of biomaterials for the removal of heavy metals and/or radioactive waste from aquatic environments.

## Supporting information

S1 Fig COG classification of all unigenes in the *P*. *viridis* transcriptome. (PDF)

S2 Fig GO annotation of all unigenes in the *P*. *viridis* transcriptome. (PDF)

S3 Fig The labeled spectra with MS identification information of all identified unique peptides. (PDF)

S1 Table Nucleotide sequences of primer pairs for the RT-PCRs. (DOCX)

S2 Table Nucleotide sequence of the modified Pvfp-5-1. (DOCX)

S3 Table Summary of the assembled foot transcriptome of *P*. *viridis*. (DOCX)

S4 Table Statistics of functionally annotated unigenes in the foot of *P*. *viridis*. (DOCX)

S5 Table Summary of the proteome data from the byssal samples of *P*. *viridis*. (DOCX)

S6 Table Byssal thread proteins identified from *P*. *viridis*. (XLSB)

S7 Table Byssal plaque proteins identified from *P*. *viridis*. (XLSB)

S8 Table The precursor mass, mass error, and E-value of partial unique peptides from identified proteins. (DOCX)

S9 Table Byssal protein sequences identified from *P*. *viridis*. (DOCX)

S10 Table The KEGG pathway annotation of byssal proteins. (XLSX)

## Abbreviations

ALP: Antistasin-like protein
BLAST: Basic local alignment search tool
Cd: cadmium
CDS: coding sequences
COG: Clusters of orthologous groups of proteins
FDR: False Discovery Rate
GO: Gene Ontology
KEGG: Kyoto Encyclopedia of Genes and Genomes
LC-MS/MS: Liquid chromatography tandem mass spectrometry
Mcfp: Mytilus californianus foot protein
Mefp: *Mytilus edulis* foot protein
MTs: Metallothioneins
NGS: Next generation sequencing
Nr: Non-redundant protein database
Nt: Non-redundant nucleotide database
PreCol: Precollagen
PSM: peptide spectrum match
Pvfp: *Perna viridis* foot protein
RNA-Seq: Transcriptome sequencing
RT-PCR: Reverse transcription PCR
SDS-PAGE: SDS-polyacryl-amide gel electrophoresis
SPI-like: Serine protease inhibitor like protein
