# Supplementary figures and images for: High-throughput identification of heavy metal binding proteins from the byssus of chinese green mussel (Perna viridis) by combination of transcriptome and proteome sequencing
Source: PLoS One. 2019 May 9;14(5):e0216605. doi: 10.1371/journal.pone.0216605 (PMC6508894; doi:10.1371/journal.pone.0216605)

**Fig S1. COG classification of all unigenes in the *P. viridis* transcriptome**

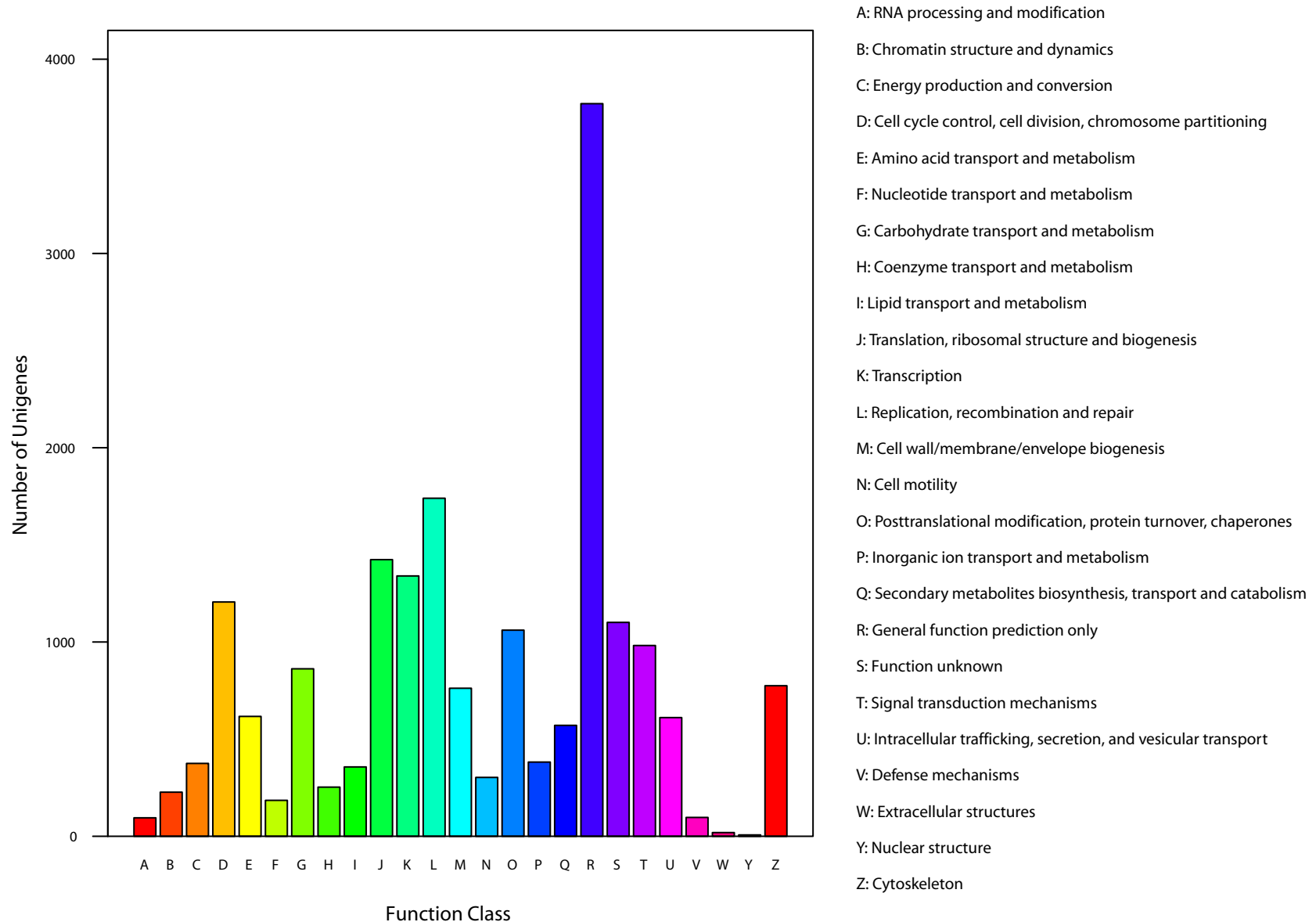

Supplement: S1 Fig — (PDF) [file pone.0216605.s001.pdf]

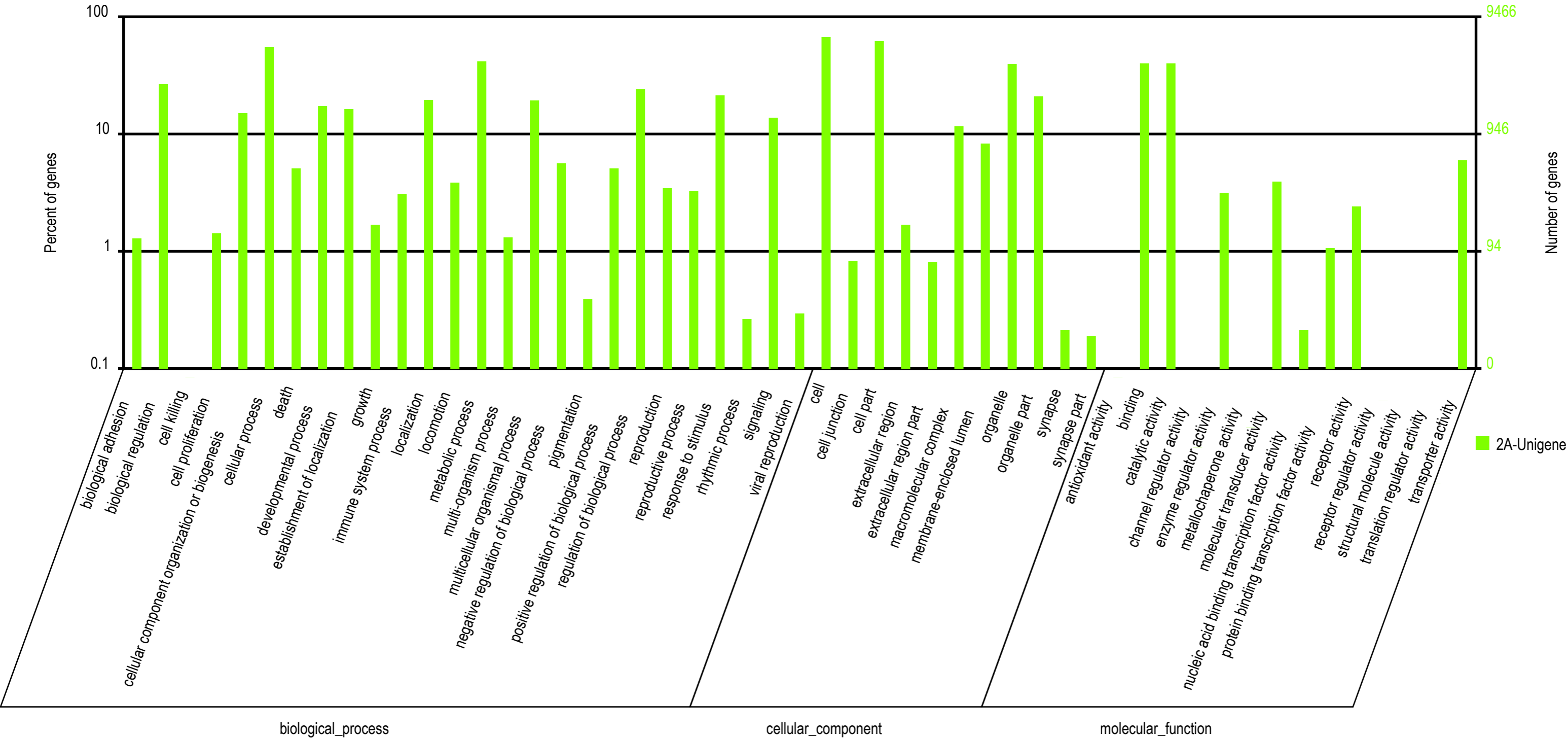

Supplement: S2 Fig — (PDF) [file pone.0216605.s002.pdf]
