# Supplementary material for: High-throughput identification of heavy metal binding proteins from the byssus of chinese green mussel (Perna viridis) by combination of transcriptome and proteome sequencing
Source: PLoS One. 2019 May 9;14(5):e0216605. doi: 10.1371/journal.pone.0216605 (PMC6508894; doi:10.1371/journal.pone.0216605)

Fig S3. The labeled spectra with MS identification information of all identified unique peptides.

Unigene23727_2A

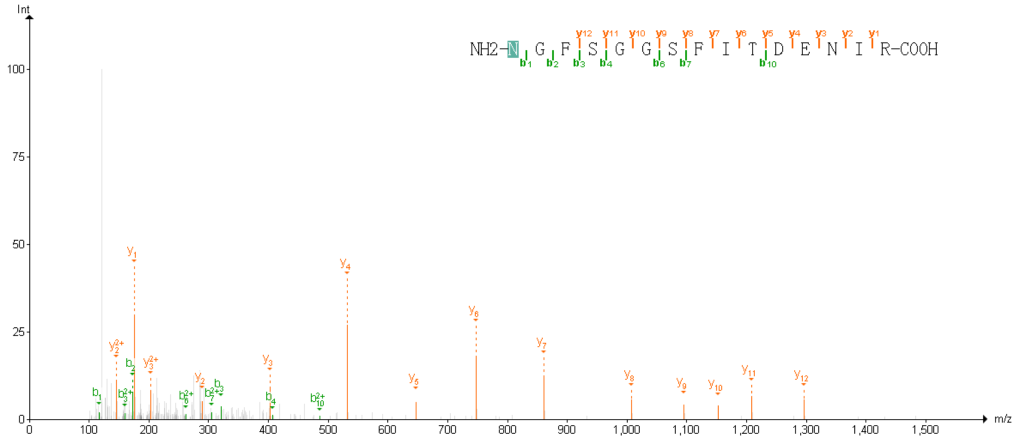

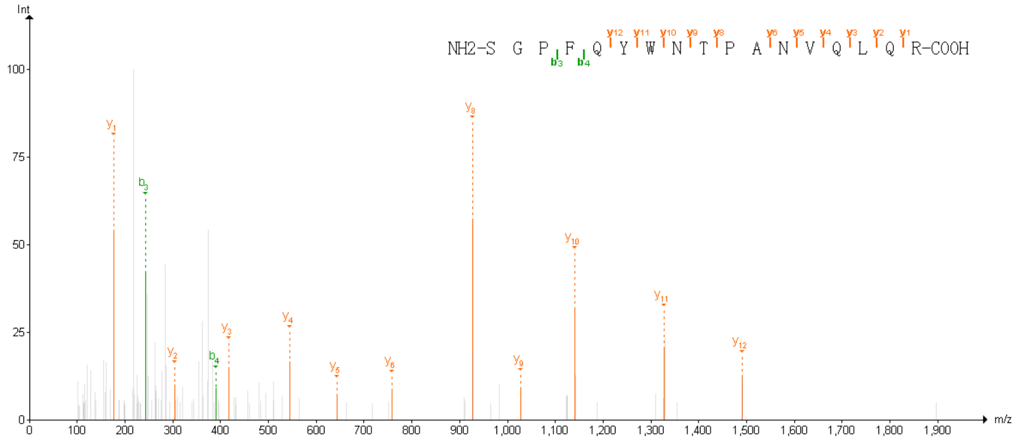

Unigene23721_2A

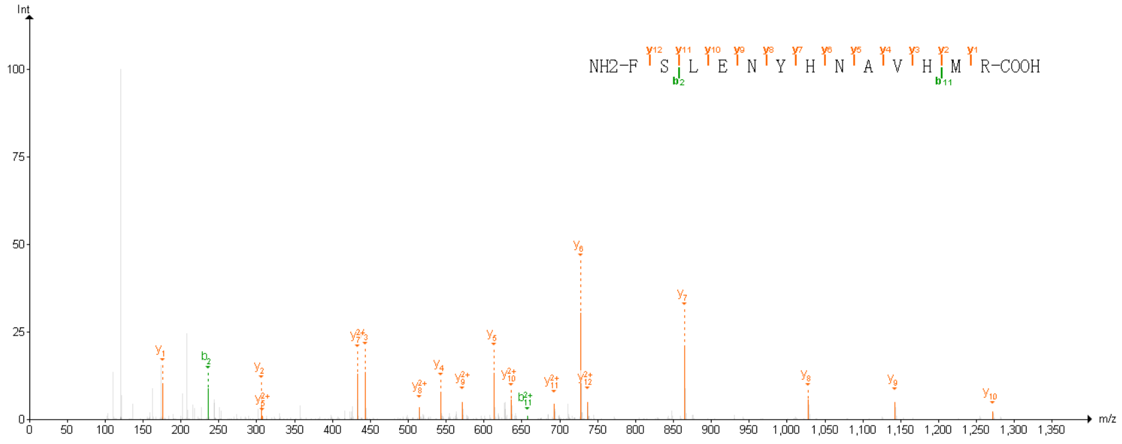

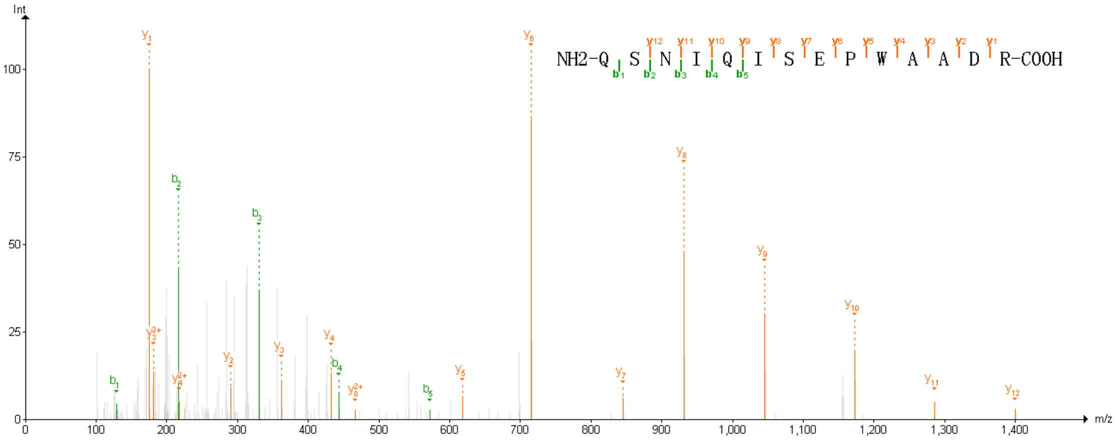

Unigene23611_2A

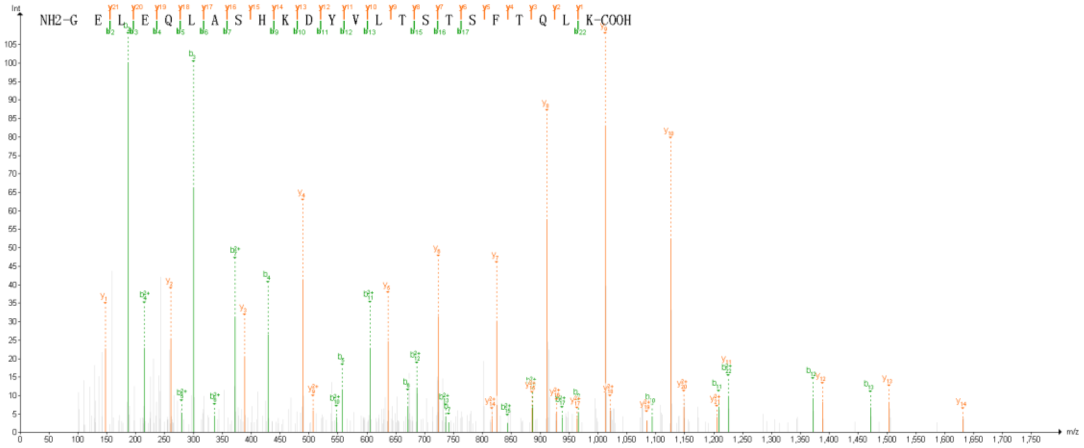

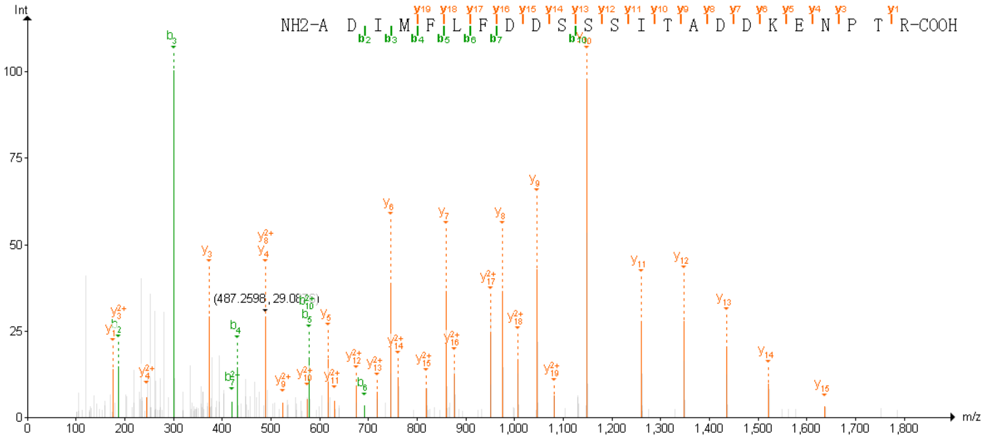

CL121.Contig1_2A

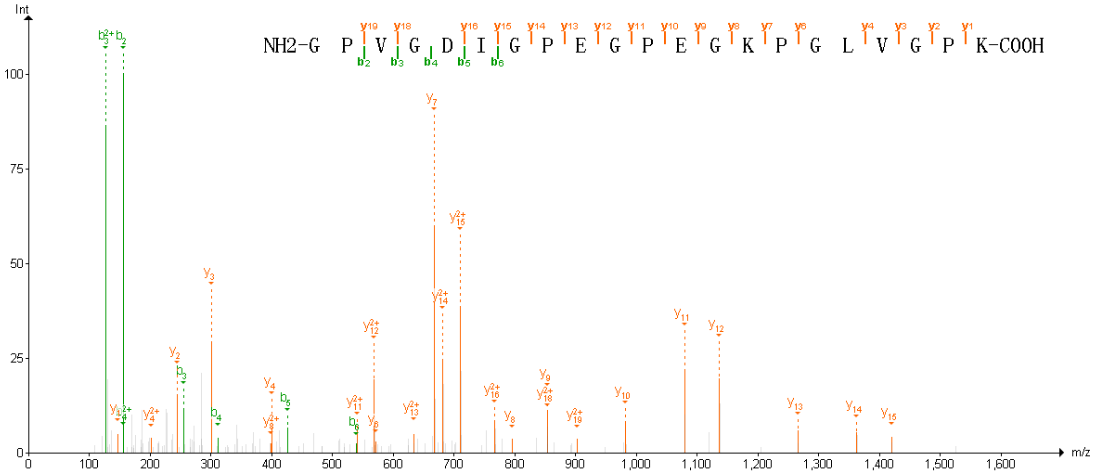

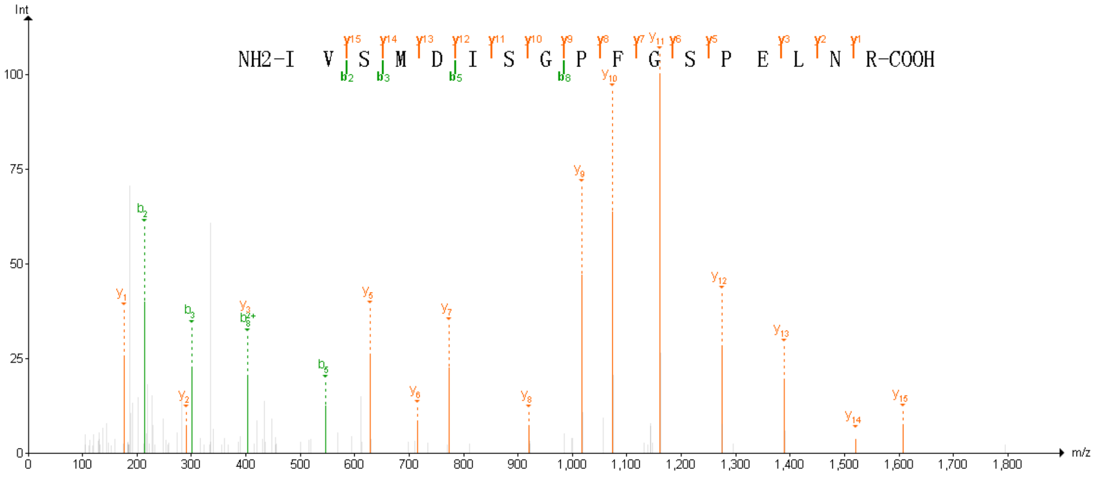

Unigene26029_2A

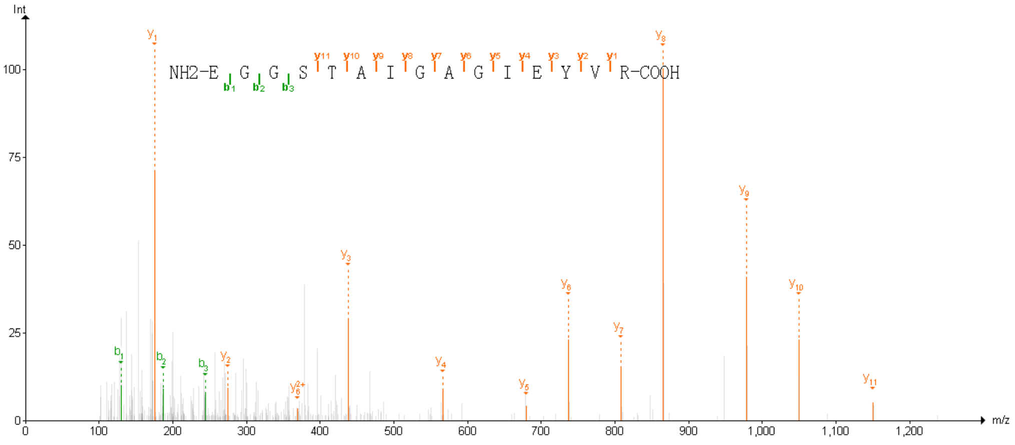

Unigene23611_2A

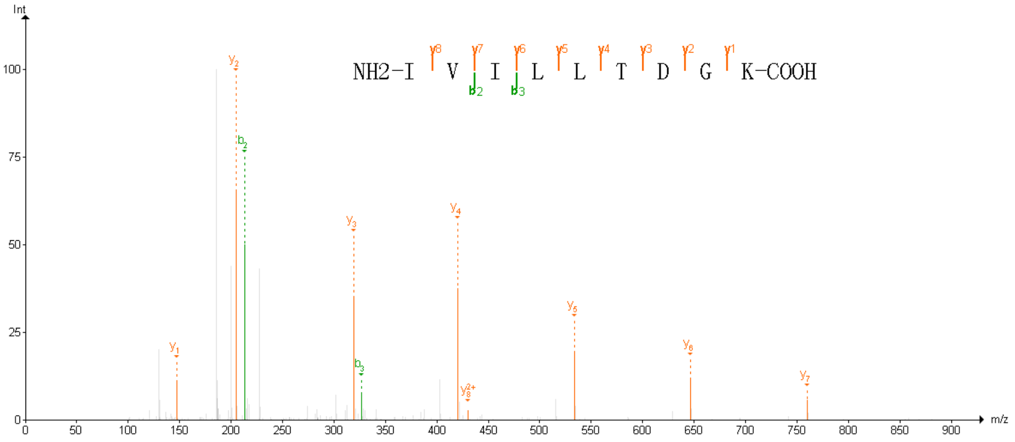

Unigene25995_2A

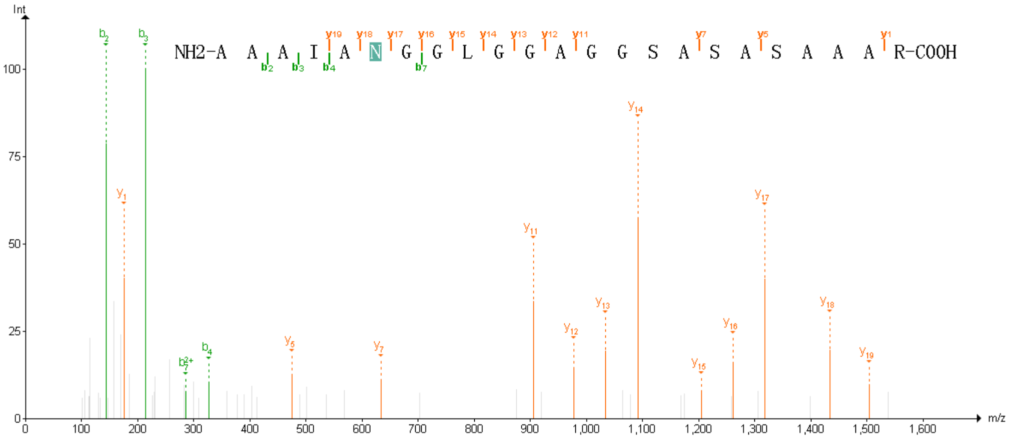

Unigene25716_2A

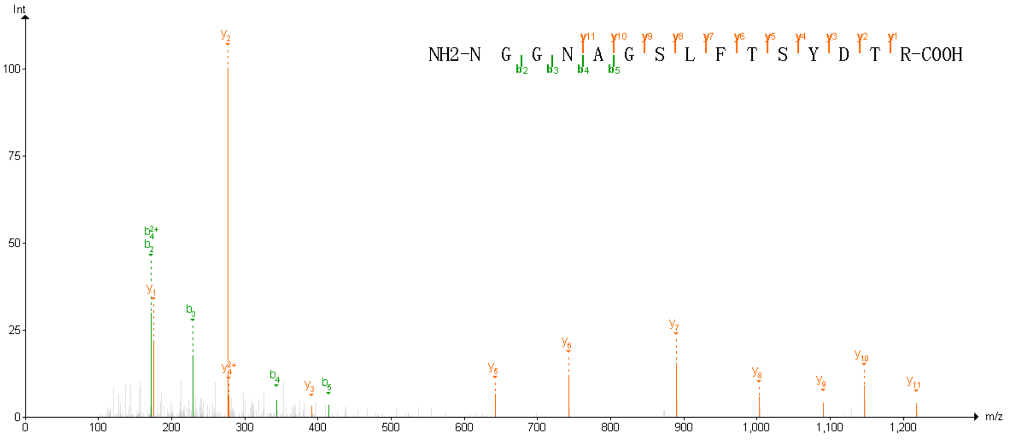

Unigene23933_2A

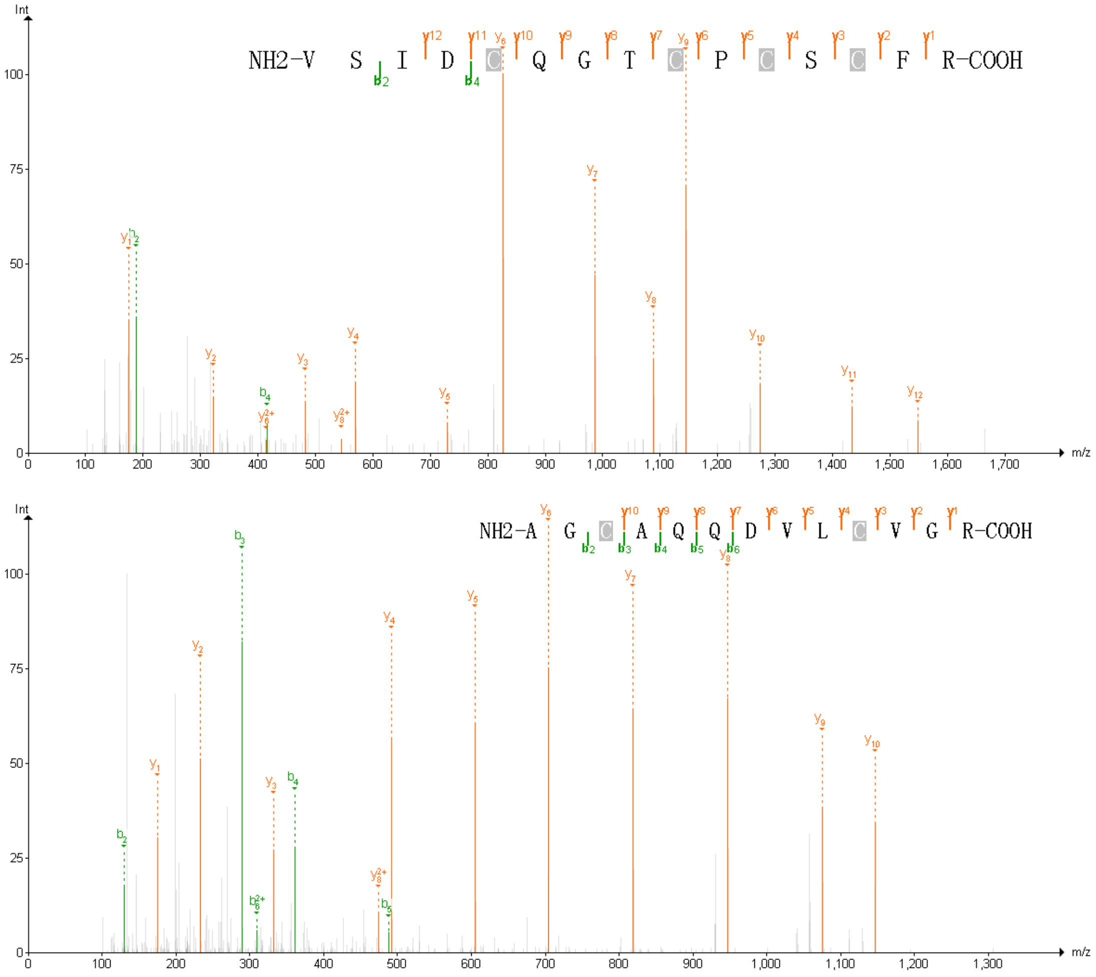

Supplement: S3 Fig — (PDF) [file pone.0216605.s003.pdf]
