# Supplementary material for: High-throughput identification of heavy metal binding proteins from the byssus of chinese green mussel (Perna viridis) by combination of transcriptome and proteome sequencing
Source: PLoS One. 2019 May 9;14(5):e0216605. doi: 10.1371/journal.pone.0216605 (PMC6508894; doi:10.1371/journal.pone.0216605)
Supplement: S1 Table — (DOCX) [file pone.0216605.s004.docx]

**S1 Table** Nucleotide sequences of primer pairs for the RT-PCRs

| **Gene name** | **Forward primer (5’ to 3’)** | **Reverse primer (5’ to 3’)** |
| --- | --- | --- |
| *ALP* | ATGAGATGTCCCTATGGCTTT | GGCTGGCGGGCATGGTTTACA |
| *Pvfp6* | ATGATAAGTGCAGTTTGTATATATTTC | TTTGTATCCATAACCACATGC |
| *Pvfp5-1* | ATGCTAAAGTTTGTTGTATTGGCAG | GTAGTATTTACCACTGCAGCGTTTT |
| *Pvfp5-2* | ATGCTAAAGCTTGTTGTATTGATTA | ATAGTACGGATCAATGCAACG |
| *Pvfp3* | ATGAAGTGCACACTATTTTCTATCT | GCCGTATGTACAGCCAAAGC |
| *Oikosin-like* | ATGAAGCTCGTGCTGTTGG | CGTTGAAACGCATCGCA |
