# Supplementary material for: High-throughput identification of heavy metal binding proteins from the byssus of chinese green mussel (Perna viridis) by combination of transcriptome and proteome sequencing
Source: PLoS One. 2019 May 9;14(5):e0216605. doi: 10.1371/journal.pone.0216605 (PMC6508894; doi:10.1371/journal.pone.0216605)
Supplement: S2 Table — (DOCX) [file pone.0216605.s005.docx]

**S2 Table** Nucleotide sequence of the modified *Pvfp5-1*

>modified *Pvfp5-1* nucleotide sequence GGATCCTACGACTACCGTGACCCGTGCAAACCGCGTCCGTGCGTTAACGGTGGTACACGGTAAAAACTGCCAGTACAACTCTTGCTCTCCGTCTCCGTGCAAAAACGGTGGTACCTGCAAATGCCTGGGTGGTTCTAAATTCCGTTGCTACTGCAAAAAAGGTTACAAAGGTAAATACTGCCAGTACGGTCCGTGCTACACCAACCCGTGCCTGAACGGTGGTACCTGCGCTTACATGTACGGTCTGCCGTTCTACAAATGCTCTTGCGTTCCGGGTTACTACGGTAAAAAATGCCAGATCAAACGTTACTACAAAGACCGTTGCGGTGGTTGCCTGAACGGTGGTAACTGCATCTGCAACAAATACGGTAAATACTTCTGCAAATGCAAATCTGGTTACTCTGGTAAACGTTGCTCTGGTAAATACTACCTCGAG
