# Supplementary material for: High-throughput identification of heavy metal binding proteins from the byssus of chinese green mussel (Perna viridis) by combination of transcriptome and proteome sequencing
Source: PLoS One. 2019 May 9;14(5):e0216605. doi: 10.1371/journal.pone.0216605 (PMC6508894; doi:10.1371/journal.pone.0216605)
Supplement: S3 Table — (DOCX) [file pone.0216605.s006.docx]

**S3 Table**  Summary of the assembled foot transcriptome of *P. viridis*

| **Parameter** | **Number** |
| --- | --- |
| Total number of raw reads | 55,670,668 |
| Total number of clean reads | 53,047,718 |
| Total number of clean nucleotides (bp) | 4,774,294,620 |
| Total number of contigs | 164,473 |
| Minimum unigene length (bp) | 200 |
| Maximum unigene length (bp) | 14,157 |
| N50 of total unigenes (bp) | 794 |
| Average unigene length (bp) | 599 |
| Total number of unigenes | 73,571 |
| Length of all unigenes (bp) | 44,037,451 |
