# Supplementary material for: High-throughput identification of heavy metal binding proteins from the byssus of chinese green mussel (Perna viridis) by combination of transcriptome and proteome sequencing
Source: PLoS One. 2019 May 9;14(5):e0216605. doi: 10.1371/journal.pone.0216605 (PMC6508894; doi:10.1371/journal.pone.0216605)
Supplement: S4 Table — (DOCX) [file pone.0216605.s007.docx]

**S4 Table**  Statistics of functionally annotated unigenes in the foot of *P. viridis*

| **Hit database (E-value≤1e-5)** | **Number (percentage) of annotated unigenes** |
| --- | --- |
| Nr | 29,937 (40.74%) |
| Nt | 6,721 (9.14%) |
| Swiss-Prot | 22,988 (31.25%) |
| KEGG | 18,615 (25.30%) |
| COG | 8,834 (12.01%) |
| GO | 9,466 (12.87%) |
| Total | 31,710 (43.10% of the total 73,571 unigenes) |
