# Supplementary material for: High-throughput identification of heavy metal binding proteins from the byssus of chinese green mussel (Perna viridis) by combination of transcriptome and proteome sequencing
Source: PLoS One. 2019 May 9;14(5):e0216605. doi: 10.1371/journal.pone.0216605 (PMC6508894; doi:10.1371/journal.pone.0216605)
Supplement: S5 Table — (DOCX) [file pone.0216605.s008.docx]

**S5 Table** Summary of the proteomics data from the byssal samples of *P. viridis*

| **Sample** | **Total spectra** | **Identified spectra** | **Identified peptides** | **Identified proteins** |
| --- | --- | --- | --- | --- |
| Thread | 55,566 | 5,307 | 994 | 153 |
| Plaque  All | 67,086  122,652 | 1,422  6,729 | 335  1,031 | 103  187 |
