# Supplementary material for: High-throughput identification of heavy metal binding proteins from the byssus of chinese green mussel (Perna viridis) by combination of transcriptome and proteome sequencing
Source: PLoS One. 2019 May 9;14(5):e0216605. doi: 10.1371/journal.pone.0216605 (PMC6508894; doi:10.1371/journal.pone.0216605)
Supplement: S8 Table — (DOCX) [file pone.0216605.s011.docx]

| **Protein_Accesstion** | **Peptide** | **Experiment_Mass** | **Theory_Mass** | **Delta(Da)** | **Evalue** |
| --- | --- | --- | --- | --- | --- |
| CL121.Contig1_2A | IVSMDISGPFGSPELNR | 1817.9 | 1817.898 | 0.00145 | 9.31E-10 |
| CL121.Contig1_2A | GPVGDIGPEGPEGKPGLVGPK | 1956.029 | 1956.032 | -0.00294 | 7.68E-09 |
| Unigene23611_2A | GELEQLASHKDYVLTSTSFTQLK | 2594.325 | 2594.323 | 0.002258 | 3.84E-09 |
| Unigene23611_2A | ADIMFLFDDSSSITADDKENPTR | 2587.178 | 2587.175 | 0.003067 | 2.56E-13 |
| Unigene23721_2A | FSLENYHNAVHMR | 1616.752 | 1616.752 | 8.20E-05 | 7.04E-07 |
| Unigene23721_2A | QSNIQISEPWAADR | 1613.783 | 1613.78 | 0.003413 | 9.17E-08 |
| Unigene23727_2A | SGPFQYWNTPANVQLQR | 2004.981 | 2004.981 | 0.000322 | 6.70E-06 |
| Unigene23727_2A | NGFSGGSFITDENIR | 1612.749 | 1612.748 | 0.000714 | 9.42E-08 |
| Unigene23727_2A | NGFSGGSFITDENIR | 1613.734 | 1613.732 | 0.001826 | 1.76E-07 |
| Unigene23933_2A | VSIDCQGTCPCSCFR | 1845.728 | 1845.727 | 0.001063 | 7.39E-08 |
| Unigene23933_2A | AGCAQQDVLCVGR | 1432.654 | 1432.655 | -0.00087 | 5.25E-08 |
| Unigene25716_2A | NGGNAGSLFTSYDTR | 1558.701 | 1558.701 | -0.00055 | 1.37E-07 |
| Unigene25716_2A | NGGNAGSLFTSYDTR | 1559.686 | 1559.685 | 0.001277 | 1.74E-07 |
| Unigene25995_2A | AAAIANGGLGGAGGSASASAAAR | 1828.896 | 1828.903 | -0.00698 | 3.78E-13 |
| Unigene25995_2A | AAAIANGGLGGAGGSASASAAAR | 1828.901 | 1828.903 | -0.00124 | 2.04E-07 |
| Unigene26029_2A | EGGSTAIGAGIEYVR | 1478.737 | 1478.737 | 0.000897 | 5.97E-07 |
| Unigene26029_2A | IVILLTDGK | 970.6062 | 970.6063 | -4.90E-05 | 2.43E-07 |

**S8 Table** The precursor mass, mass error, and E-value of partial unique peptides from identified proteins
