# Supplementary material for: High-throughput identification of heavy metal binding proteins from the byssus of chinese green mussel (Perna viridis) by combination of transcriptome and proteome sequencing
Source: PLoS One. 2019 May 9;14(5):e0216605. doi: 10.1371/journal.pone.0216605 (PMC6508894; doi:10.1371/journal.pone.0216605)
Supplement: S9 Table — (DOCX) [file pone.0216605.s012.docx]

>Unigene2417_2A 44 1759 minus strand thioester-containing protein [Euphaedusa tau]

VLCLLSVVGFVSSKNTYIATVPRYIRPGEDAEVCISAIRRVTKSVTIALTLLGKNNTVVQSSKSKKFIRKGPVTVNIKVPLNIGQNEYSLRVKASGGLNFDKTARDINVKWKSSSIFIQTDKKAYRPGDLVQYRVFGTDSNLNVLKDPINIFISDPNDNTVQQHLMVQPSYGLYTDSFQLSTLTTLGNWYIQVKQRKETTYKSFTVQQFVLPRFYVSLILASFQLTTERFFRIGIITKYTFGKPVQGDCKLQIKQRWNDKEGIIKKFKINGTVKVKVLMSDVYKLGSSLEVIATVTESISGISAKDSKSTELHTTPEKVTFSSSMADTFKPKLPYNIILRVTQHDDSSLKPPLGRVNITVTYKLPKPTQPPGSHQDSSASLPTPVSSLPTLLWSRNIQIPDSRIITATAVFPVRAVSADIEANYRKASTSKYPDKARSPSNNFIQVSIIKDQKAVPNKTLPLKIQTTQRNIPFVYYKVVSKGGLVANGRFRLRRGNGKVNLRVTYAMTPTAKLLVYYTRQNGEIIADAITFHVDNIFRTKVDIRFDKRKALPGEDVTLNIKSTARSLVNVLA

>Unigene3127_2A 3 533 papilin, isoform F [Drosophila melanogaster] >gi|272477222|gb|AAF56794.4| papilin, isoform F [Drosophila melanogaster]

PKNEGQCSASRLKWYYDINDMECKQFTYTGCRGNRNRFGSLNKCRRRCNKVVKDACPPQTPCRRPQPDECAGLAYIFKNGVRCAFGCIYPPCKPGNCPGGQPASSCIVCELNRDCDGSQICCEGCCRIPVIDPKRGKCPTTGLRKCIGTSSECSLDSQCTGKMKCCSIGELKCCVDP

>Unigene6759_2A 69 227 serine protease inhibitor 2 [Rana chensinensis]

CELPKKRGTCNARIERWFFNIRIGKCRKFIYSGCNGNPNNFLTKEDCNTACNA

>Unigene9204_2A 42 638 minus strand bactericidal permeability increasing protein [Crassostrea gigas]

KILFLVSLFCIVQLTLGIHNANINIAGKTLQGMIKNDMLSLIKIPDYKIKKGKVTSIKIVSLTLPDVKITERGTTLTVQISNFGVKLEANWRVKFKIVLVSVKGKGTLKASANGVGITITLSKTTLQVIRCSDVIKKLDVDFKGKNFVGKVLTFITKTFKKRVIKFFKRQLKGKICAAVRRLIDSNAAKIAKILGITLK

>Unigene9523_2A 1 681 minus strand PREDICTED: nematocilin B [Hydra magnipapillata]

ELIRERDDFKARWHQEQKSRKDVEERFAAYSKKAEFDADVLRQQINDLKQRLEATTSTILALETRIRELSKTDTNIPQLLSQVRRAAEEELRKHETDIEQKYSKNINSLKQRMDNDASVIGRLEQEKTQILGSVGDLKARISDLESQVSALAQHKQSLQDLIAQERATASAQIREMDRRFKELQDDFFAKLNDARFSKDSFIPLKAEIEAMKLLLTEEERRLSAPIN

>Unigene9553_2A 133 870 minus strand Hypothetical protein CBG05433 [Caenorhabditis briggsae]

YHSETRDSAHKGVAFAGWHRFFLVLYELALQEKVASVMLPYWDFTLDREMKNPVDSVIFTDLFLGKSDNENGEVISGPFGHWRRKIIRSVGKREIAKRSYIEQMIERSPNQRRFVADLESVHDHVHSWVGGDMAFVGNAPRDPIFYMHHAFIDCVWEMYRDREKRLGRNPEIYPQTNDTLHDPNVIMLNLTINGVFQRNADGYKNFWTEIYYTCAPFPSCSFVSPDCGSEWLQCNVFGDTARCESK

>Unigene9567_2A 124 498 minus strand hypothetical protein BRAFLDRAFT_84494 [Branchiostoma floridae] >gi|229299135|gb|EEN69755.1| hypothetical protein BRAFLDRAFT_84494 [Branchiostoma floridae]

LLIVYLPLIYGTELSYNYSDQMAQTYCQGRFVPGWVFAIRRDCRGTAPTCNTVCATAKSDILGFIRFQRNNVACFDAIQVKKGHVQLAVNPTGSKADAGMATYGYGAGGCTWKPNHCGPNYCCCK

>Unigene9796_2A 203 853 PREDICTED: papilin-like isoform 1 [Acyrthosiphon pisum]

GKDENCTEGRMCCGVQCGTACVEPNVCTLPKVVGACNSKVQRWYYDLSEGKCRRFNYSGCNGNTNNYKTEKECRKGTKQCACLLENKTGPCKASIRMYFYNYRTHRCEEFIYGGCKGNKNRFETKSDCKEGCKSILEASTPLPPLLPPKENCEYGIPHRIKTCNRSAVSDCPPEHYCENHPDAKVTACCPNAVPSSKPGVCPNPGPNKPCDKKCKAD

>Unigene9798_2A 260 457 PREDICTED: similar to ovulatory protein-2 [Gallus gallus]

DCDGTKKCCDNGCGRVCLEPLGPKVGFCQVIPPATPGVCVNYCNDDKQCPGSEKCCSNGCGKSCKI

>Unigene9861_2A 83 424 PREDICTED: histone H2A.V-like [Loxodonta africana]

KKMAGGKAGKDSGKAKAKAISRSQRAGLQFPVGRIHRHLKHRTTSHGRVGATAAVYSAAILEYLTAEVLELAGNASKDLKVKRITPRHLQLAIRGDEELDSLIKATIAGGGVIP

>Unigene10173_2A 42 617 minus strand PREDICTED: similar to GH18457 [Hydra magnipapillata]

FILYGILLPCCFRMSMTCSCFIHRRVHMQNLFCSKKFLFLGRVYREEVVDGFLFERVKYSVKVIKPYKGSLEMGETFDIYTGSGSESCAIKIKNGTVRYFMTDDDKTISPCDSEGSPRTFRQWLYVVSKGRYSYAANCRCKVKDDMYQNPDGRQSCKYDINNRPLHRCYTDLALCVWNGDRCVWDNELSCKQ

>Unigene10233_2A 285 1937 minus strand CRE-MLT-7 protein [Caenorhabditis remanei] >gi|308258220|gb|EFP02173.1| CRE-MLT-7 protein [Caenorhabditis remanei]

IGIGPYKRRCRRVMNKYRRLDGQCNDFDSPAAGSLLYRFGRNIPLENTPDTFCNLYTPNPRDISRTLLQRKEFIPATSINLLVAGWIQFMTHDWFDSGENDRSRTLQVQVREDDPDFVSTIKIPMTRPDSCVKIPGLTKMMCQKLPPNLKMCKNVKDYETYQNTVTHWWDASQIYGSNRRTNRRVRSGRGGKLVLTQEGRLPLDRSTGLPITGQNNNWWVGLELFHIIWTKEHNYVCDQLKQQNPRWNDKKLFETARLIISAVMAKIHTMEWTTAILQNYVLKLGLKSNWYGFTMLDVAQGNRTLAESLSQQFPELANGIPGAVGNPKNTRGVPFSLTQDFVAVYKFHPLMPDYIVIRDRNGGTELNRYNMTDVVFEKTDKVFRENSIEDIISSFGNANPGAMALHNYPNFLRHLFLPSHSPHGEGYVDLATIDLMRERERGVPRYNEFRRQLHLTPLSSFEDLTPNKEHVEELRKAYNNDIEQIDLLVGSLAESPLPDGFGFSDTFNRIFIVMARRRMEADRFFTDDYTSEFYTQWGLDYVDKTYMKEIL

>Unigene10680_2A 183 1421 PREDICTED: dihydrolipoyllysine-residue succinyltransferase component of 2-oxoglutarate dehydrogenase complex, mitochondrial-like [Meleagris gallopavo]

YIRFTKKYCCNSQLIIKRNFHVTNRYFDDVLTAATPPFADSISEGDVRFEKAVGDFVKEDEIVCEIETDKTSVPVQAPKSGIIQSFLVDDGATVQAGTPLFTLKLSDSPGESAPESVAASEKPPPPAVTSKAPETPVATPASGPIPTTPPPPQPIPKAPISTKPLDSIKPIPATDAPVMGARTEKRVKMTRIRQKTSQRLKAAQNECAMLTTFNEIDMSNVIEMRNQYKEAFQKKYGLKLGFMSAFVKAAAYALTDQPAVNAVIDESEILYRDYIDISVAVSTPKGLVVPVIRNVGSMNYADIERAIAELGEKARTGSLAIEDMDGGTFTISNGGVFGSLFGTPIINPPQSAILGMHAINDKPVAIKGKVEIRPIMVVALTYDHRLIDGREAVTFLKKIKSAVEDPRVLLLDL

>Unigene10729_2A 374 1921 tyrosinase precursor 2 [Illex argentinus]

IRKSYHSLSAQERRRFHRAVLAMKADTRVSPNAYEALANIHNENALYAAHFGPAFCPWHRYYLYLFENALRRYDASVTIPYLDSTYEDLIGSNTMNSILWTEGYLGNVDGNLRTGPFKNWRYRTTAGFTVPVTRNMGSGPGWFTRQCLQEVINHTRFPRVFERLETCHNYAHVQIGGTMNDATVSPHDPIFFLHHCFIDYVWEKFRENSRRAIGNFYQYDYPIAGDPEHLPHRRMVNLNELKNRQGMSSLFTDYIYQYEEPTYSCTTTCPGDALVCVRGTCVPRTSVTGSVTNIGRKKRSTDETIPIEHIKTYLDQSYQNDFVIDGIADVQGWCIIPVALMYKRPIGTSFGCKPIREGYIDETTDIYSINNSSEAWNVFTPGKTKFVNSDSGSGATKIFLQADGITYKGRYIDYGIVDSRQIVSETIGYVGVKHPKNGPVIAYLSAYDTHGHVCQPHCIDESSTTLHYKKCSGVIKVTNEEPKMFGNDIGDAILYRYNFTDQSNEPRSRYRDQFIM

>Unigene10883_2A 348 5027 minus strand PREDICTED: SCO-spondin-like [Xenopus (Silurana) tropicalis]

CFNFTSYVLGGYQLDNEKSFPGKTDKQINQILMEEMGKSDSSTMPCYYYKEVTRKVQECCAGWGGTNCDQPVCTNECKNGGVCTGPNKCTCTNGYTGYMCEDLLEVANSDRQYCFVGPNCNSAKAVPGTHTQGDCCNIPEAHSWGVGDKCHPCIKTQVEGIVSQQVKLDFRTCANFGASYYRTFDGVEFSFGGRCTYTLAMEKVTTGQPRWHVQSTVINCASSVDQCKKRITVEINGETLTLEGGNVWVDNKEFLLNLEQPRETPGKTATIMDKGDWVFVSSHIGVSLKVDAKSAVYITLEKSRISAGSVIGVCGNFNDDPSDDKITRSGETAPNMAYAANTWQIANEAQPCQGAGTTPDYCETTADKQLAEQACSTILSGIFGECHAMISPYMLYHMCMNDVCRSKNDTDVTKCAYTSNFAQSCASLGVVVFWRSSTYCPKTCEDGKVYRECSSKCPRTCKALYSVMPDSCFDDCVSGCECPVGQFIQDGVCVTADQCECEYNRQRYKSGETVKNGCNTCKCLMGRWQCTEDKCSEVCQVIGLNNIKTFDNSEYSFMPQGSCQFDVVKPIDTPTVLDDPRSHLHITLHSHQCPNMMSGIFCMNKIVIKYRDTRVTLEGKNVKVEDLVNNQNIDLTNSIDNQPYHTKHMYVKAATDKYRMVQAFGVKVLYDMRRAFYIYLNPFYINKVIGLCGKYNYQYLDDFSTQNGLPESNVHDFVSRMCDSGCTISPQEKEDYAQGNSIGNAKEDCRFLDPTQGETGSGLFAACTKYTDKMSYYYDKCVEDTMLRGGLNVKETICDLVAAFARHCAQYGIPDSNWATESYANYDFLQVHCADWVRCEKDGQTYTECGRLCDASCRDFAIADASCEEECIPGCQCPKSEYLSDHGQCVVRSKCTCFDKYTNRLYEPGANITRQCAICSCVNGEWNCDQDNCEDIVCPRNQKHITADSYCSVSKTCANYDLPLTCTNDVPYQGCGCADGTVMAPDGSCVEPDRCPCLYGHEYYQEGEEVTINCNKMQCQKRHFVKVGTVNCPGVCWVFGDPHYETFDGKKYAFQGSCKYIMTRAKDSTFSVTTENVPCGSTGVTCTKSAHITIKNTVIHLIRGENVTINGKAFLNSQYISEGLEVGTFSYWTVISAKALGIEVRWDGGTRLGVALDKSKWMNKVEGLCGNFDGMSESGELIKQEQDVATNVLDFARSWTVDTTCDETASAEPTGPPCQDSPNREKWAQASCGIIYDAVFKDCRDAMAEQLVTAAYEECLYDSCKCDMGGDCECLCTAIANFAEKCNQQGKPIKWRSPKFCPIMCEYGYEYKACAKPCPQTCKNIGDEPDSFCESSFCMEGCFCPDGMVETVQYGKKMCVPASQCNCVHNHREYPAFTSLLMDNCLNCTCINGKFECVGESCEEKCTENQFQCNDNTCINNMYRCDHHMDCKDGSDEANCTYNCTIEQHRCDNGQCISKSHRCDGMSDCLDDSDEMYCEVECDKDEFTCDSGRCISIKHRCDGSYDCGREDLSDEKNCTKKEECQENHGFHCGETMVCVPLTKLCDRHDDCGNGLDEVGC

>Unigene10994_2A 46 1326 minus strand RecName: Full=PIF; Contains: RecName: Full=Pif97; Contains: RecName: Full=Pif80; AltName: Full=Aragonite-binding protein; Flags: Precursor >gi|256252203|dbj|BAH97338.1| Pif177 [Pinctada fucata]

LKMASIHLLVLLVAGFSSAEAAPKTVAKCDKTGDVVFSIDGSESIKRRDFPKLIQFTLNIVDSFKISADKAQVGAMVTSSEINDYFEPLDIAADVKSKISAFVHPRNSSRTDLALAYLRARLLKTSRARVGVIISDGDSKFSDLTKSEAESARKEGISLIAIGVGTGPDEEKELENIAGSKAMYRSITDINKLTDAATLKDIKELICNVLRTSRRLTYPVEPDEIIIDPLCDGCIIANGFGYNAYKHDCSKFVQCYPSQDSKGVKVMTSTVKQCAYGTFWSQDDISCVEPENTECKYSICKGKSDGYTLPYIGCKTYWNCNKGIPEAKCCAAGHYYDNKRATCVVGVPSGCSGVECGEDAAAKKKKLRLCEFEGYKKDPAKYYQILKISSSKTMKLLRSCAPGTIFDERKCSCVFDINGISKSTDCN

>Unigene11647_2A 63 2399 minus strand peroxidase-like protein [Euprymna scolopes]

QPTFAAIAAEKAPAASHDFNVPESELAAPRADLSFSQEANMNTMIKGTLDPASIKAVSDIEKGGSIHIPGIQLPEKTSPHIQAPSRSVMESAVKTVQAKIQIEDATNQKAASVDTANEKTQGFGPHLLTSAEAIRISESARIIIDTAKEAMDSLKVPKEDRRKALEAIPSDFVKSIQGQSRCSYTAGPCVATSQYRTADGSCNNLNKPVWGKSHTPFERFMPSFYEDGVDDPRSLDIFGDPLPGPRTISLNVHTDSDVTTQMPDLGHFSMEFGQFVSHDIQMNALSKGGYNSNLNCCAFPNRRNCFKIDLPTNDPFYSIFNRTCMNFVRALGVTDLDCSVGPRQQMNQNTHYLDGSALYGSTQWVANSLREFSGGRLKSANSGELLPKDTNNAASCILPPNPNVKCFKSGDPRVNQQPALMALTTIWMKEHNRIAARLLQLNPSWTDTKVYNEARKIVGALIQHVTYNEYLPHILGNQEMTNFGLNSLTSGYFTGYDDTVKPQIRNSFSAAAFRFGHSMVQQKMAYNGPGHVNQSPLLHNEFLRPDKLYDVNGGVSSVTRGLYEEFSQKVDRKLTKELTERLFERASGTGNDLAAINIQRGRDHGIASYNLWRSACGLTPADTFTAGVSGGLEHHTPAAALALENVYKNPWDIDLFTGGVSEIPVSQGKVGPTFACIIGLQFKALKVGDRFFYESDTNVKFSPNKLAEIRKATMARIICDNTDINNIPVDVFQKTSPSNPEITCSSLPEIDLTPWEECINGGWSPYRTLGPCYKVRKCD

>Unigene11867_2A 25 2634 minus strand oxoglutarate (alpha-ketoglutarate) dehydrogenase (lipoamide) [Nasonia vitripennis]

QRRGHNIASLDPLGILDADLDPEIPKELLLSSYQWDETDLDRKFTLPTTTYIGGNESQLTLKDIVKRLEDVYCNHIGIELMHVNNKEQVDWIKQKFETPGIMTLNSEEKTTLLARLVRSQKFEEFLAKKWSSEKRFGLEGCEVLIPAMKTVIDKSSEQGVDDFIIGMPHRGRLNVLSNVCRKPLEKLFCQFDSELEASDEGSGDVKYHLGMSQERLNRVTNKNVTLAVVANPSHLEAVDPVVQGKTRAKQFYRGDTDGKKVMSMLLHGDAAFSGQGVVFETFHLSDLPDYTTHGTVHIVVNNQIGFTTDPRYSRSSAYCTDGARMVNAPVFHVNADDPEAVVYVCKVAAEWRAKFGKDVVIDLVCYRRNGHNETDEPMFTQPLMYKKISKQRTILENYGEKIINEGVVTQTAYEEEIEKYNKICEEAYQNAKKETSVRNVEWLDSPWSGFFEGRDEMKHPNTSVSEDILKHIGEKVSSSPENVDIHGGLKRTLKARKKMVNDREADWALGEAFAWGSLLHEGIHVRLSGQDVERGTFSHRHSVLFDQNIDKKKYVPLNHLYDNQAEYHVHNSSLSEYGVLGFEVGYSMQNPNALVIWEAQFGDFANTAQCIIDQFISSGQSKWTRQSGITLLLPHGYDGMGPEHSSARLERFLQMSNDDCDFFPPENENFVMQQLHETNWFVCNLTTPANLFHAMRRQIHLPFRKPLIIMSPKNLLRLKEARSSFDEMTGESSFQRAIPENGVAAQNPNAVRKLNFCTGKVYYELVYEREKKGLQDKIAICRIEQISPFPFDIVKQEILKYPRASIHWVQEEHKNMGAWSYVAPRVQTIFRKMDMNTNIEYCGRTPSAAAAAGSKHRHFLELNQFLNSAV

>Unigene11889_2A 132 554 minus strand PREDICTED: catechol 1,2-dioxygenase-like, partial [Xenopus (Silurana) tropicalis]

EGRKCRPTSSDVIGPFYVSHPPKLAGMCRYRRTIRRNKHEVPLFVFGTVKSHDCKTPLGGVRVEVWQADHTGSYKNSSKCRGYRKTNKFGFYQFATIHPGRYTPDNSDSNYRPAHIHFKVIGPDGHKTLVTQMYFEDDIYL

>Unigene19899_2A 120 500 minus strand Papilin [Camponotus floridanus]

CKGNDLKCPKGQICCNHICGTACRTPVCNLPKAVSSCGPFKPVPLWYYDRKKKRCCKFKAGECSGNYNNFSSKKNCKMVKDACVCFLPKEPGSCKQNLKRFFYNPMSGKCEKFVYGGCKGNKNNFKT

>Unigene22808_2A 71 739 minus strand trefoil factor [Polyandrocarpa misakiensis]

LFAVLLVASVSYGMTLGNDETCDVQPPYRKECGWVGISQETCEARGCCFNSSTSGVKWCFQKAKLFSDNPECAVQPSNREECGWSGISQLTCEARGCCFDSSLSGTKWCFQKEKEFSNDTNCDVAPIFREECGWVGISLQTCEASGCCFDSSISSAKWCFQTAKPECWSLPNERRECGWPGISKRTCEARGCCFNSNTENTKWCFHKKQUYSSAYTRRRAEDD

>Unigene23001_2A 6 1145 Serpin-like protein [Ascaris suum]

SQYCYNRHUQMDIDTAQSSFENSLISFSHDVYKEIQIDQSECVSPYSIISALLMLMVGTGGTSKSQLRSAVLKNNWNDEFIYQQYKALGDKVFNSTENELSIATKLFVMKGLKVKQNVRDVAKNYFNAEIGHKDFSKAVKSATEINSYIAKWTKNRITDFVSPNSLSSDIVMFLVNAVYFKGVWDRAFDPKSTRKRDFFLTETNTVQIDMMYMKHYVRYYNGKDYSAISLPYAGRKYEMVIVLPRRIDGLNALKQSFSPAMALDIETKLVNMSIVVNIPKFTFESKTDLKALLPKLGIIDIFDERSADLSNLIQGIQKRLVVSEARHKVFIEVNEKGTEAAAVTGIGISITSIPPSFTANHPFLFYIKHEPTDVILFMGH

>Unigene23007_2A 67 648 minus strand PREDICTED: metalloproteinase inhibitor 3-like [Anolis carolinensis]

MERWLQIVFCIVIVSVSDVLGCSPIFPIHMQNLYCNSDRVIVGEVLSVKAIGDLKTDPFFNFGKWRYTVIVWSVLKGYGQKVVGKKRYVFTPGNDGLCKLGLKVGVSYLLGGKKQGKNMSVLSVGLNSEYFRLTIDQLFFLYTKFGDYSYGDNCDCKVTQTDNANSCAVDFSNSSCFATKDICRRKNGTCSWLR

>Unigene23025_2A 164 694 minus strand PREDICTED: similar to metalloproteinase inhibitor 3 [Tribolium castaneum]

SEECSPPGPDYVFHPQNQFCSAKFIMVGQVLSTNVKNGVRRYRVRVLAAINGFKRTDVGKVKNIYTPDSGAACGITLTIGKVYALGAHGGNDGKFSIRAFMFRLEYSKMTVQQHMYMWLKRGKLSYRQNCRCKVSRKATDKTCVPKSNDSCYADNAICSYNRKTKTCGWVNDDTCRE

>Unigene23114_2A 461 2176 Peroxidasin [Acromyrmex echinatior]

IDCPQSNITCDDSYHYRSDNGSCNNLQFPEWGMAFTAQQRYVPAAYDDGVDIPRDYDLPSPRLISNMMFNNTDGPTYDRRRTAELMAWGQLIAHDFVLTPTIDGTLKCCKDENIDNPACFPIEIPDDDEHFTTDCMNFVRSSPATEGCVPTHREQINEITSYIDGSMLYGSTTKQMNNLREFHGGRMRCTDTDLLPENINGTCLKSSSKDFCQAAGDERVDVVPNLGGNHILLMREHNRIADTLAEVNPHWDDEKIFQETRKIVAAEIQHITYAEYLPVVLGQDAMQVYELEVTNRGYDDQYNPMINPSVRNAFAAAAFRFGHSQVMPEQAYLLNDFISFQYKSLEKQFLNTHMIQKQDGRKLPELMRWLSFDKSMGTDRFFEKEVRDLLFLKNGSSTDLVARNIQRGRDHGLGSYGAYREHCGLSPVTSWNPNQPEGLVDHRWRDTRVLKDLYQNIEEIDLFVGGMTEKNVQGGLVGPTFACLIGEQFRDFKKGDRFWYERPDSRIGFTEAQLDSIKRVKLAKLFCENFGLDEIQRRVFTVVSDKNDLVPCADLPDINFTLWKDULTRRNC

>Unigene23133_2A 144 560 dopamine beta hydroxylase-like protein [Pomatoceros lamarckii]

LAVCLFFSFISVALCYPGFASKIPNGYIVPCPQGCQCNTPYWTAVGHYHNSRHTLAKNPFGKDFRLHRFQWTAELCQADSDGDGYTNGQELGDPQCQWTPGNRMVLSTPTGHPGICEPVGDIRCQHSQFTCGCLINQRC

>Unigene23208_2A 131 1279 minus strand PREDICTED: LOW QUALITY PROTEIN: HHIP-like protein 2-like [Ailuropoda melanoleuca]

CVEEIVSNFTDPLIIENAGDGSNRLFIGEQKGIIYVLDKHGSRKQNPFLDISNQIVTGGERGFLGLAFHPKYERNRRFFVYYSSKAKPKDPTYDHVARLCEFKTSSSNPNEADKSSEKLILELQQPYSNHNGGQLLFGDDGYLYVFTGDGGSAGDPLGNAQDTSSLLGKVLRIDIDKRTTSMRYGVPISNPFIRNKKFRPEIYAYGLRNPWRCGKHPGALYKGRSSKKGWILCGDVGQDKQEEIDFIRSGANYGWNAREGPECYNETLCGKIGNTTGPIFSYTHDVGSSVIGGQFYRGCQNPRLRKFYIFGDFVSGKLFVSMPTRTLEVWRAWELRICNKCRKPFTNDFEHSFTSFGLDEYGEMYVTSFGSPGKLYRIVDPFR

>Unigene23267_2A 256 774 metalloproteinase inhibitor 4 precursor [Bos taurus]

CRPSYQIHPQNRFCQSDRAIVGTVLSITKIPGELNDPAENTVEYRYTVQILAVLKGYSQKLIGQTRYVYTAVNGALCMLNLQVKNTYVLGGSIRGDHMYVNTLKLHEDASALSWQQQMYFYSVAGPYSYRRNCNCKVTTQDTASSCGISPGSPQHECYAKTGICSQVSGQCAW

>Unigene23294_2A 140 526 PREDICTED: hepatic lectin-like [Xenopus (Silurana) tropicalis]

SWTCEKGWQFANGRCYYVSRDKIEFYSALSFCNVHRGSLVEMDSIEEYKAVTRITKSQNVNHFWVGVTDMFVEGHWIKATSMTPQHFFKWRSGEPNNYGHQEHCILQEPNGNWIDYSCVTRWHFVCYKR

>Unigene23310_2A 148 690 minus strand PREDICTED: similar to metalloproteinase inhibitor 3 [Tribolium castaneum]

IAILLALAIFKLSEQCSPPGPPFVVHPQNFFCDAKYLMVGKVLSSKVGTIYTIYRIRVLAVINGFTNSDVGKVKSIYTPSSGAACGTTFRIGITYAFGANKKRDGKLVIRLSMFQYQYFKMTFQQQMYMWLRRGKFSYRANCRCRVSTDASDRTCVPKSNTYCYEKSAICSYNRISQKCGW

>Unigene23327_2A 188 1183 Tt52 [Trichuris trichiura]

CPADLTSYGRLCSHYNDKCQTNQDCIIPGHGCCYVPTCGKECFNLARPRVCPTPPPFDSDFCSTVKLAPGCSTDDDCVGEDKICCPAGKCHVKDCVYPPRYGRPNPGVCPVPRALTPLQCSRLTIVTDCIRDTDCRDGKKCCETGCDVLKCLTPMINRPECPSVKCTQYTDLCKSDKDCKYGEVCCYGENSDAYCKKCVKRDTTNGHSRRKRSSAPVPMCLTCCGPPPCCGPCISCGAGQNSSHMFCNVNESTCPEGEYCKTLHPGSHGICCAGFVIRRKRQILPNTKAGYCPPPRVDCGMAFHPDECNTDYSCPGKQKCCSNGCFKDCTNP

>Unigene23476_2A 104 703 minus strand PREDICTED: metalloproteinase inhibitor 4-like [Loxodonta africana]

IALKPLLFLSCLSVVFCCSFAPPSHPQNKFCSNDRVIIGKVLEEKFIPGEPDDLANNTANYRYTVQLVNVIKGYRKSAIGQKRYVFTPGNSALCMVNFDVGTTYYLGGNKRGTGMSMNGHDLFKRIQDVTWQQHIYLTSAGGKYSYKDNCKCQVTPTFTATSCGIPNLTRDEFCYAKTGICIEEDGRCRWFNDNCPLPVE

>Unigene23482_2A 2 1033 minus strand RecName: Full=Paramyosin >gi|4115774|dbj|BAA36517.1| paramyosin [Mytilus galloprovincialis]

ELQRTLVEVETRYKTEITRIKKKYETDIRELEGALDNANRANAEYLKQIKSLQNRNRELELQLEETQRQLDDARNQLSISERKRIAIQQELEDARSLLEHAERARKNAENELGEVSARLTEVQLQVTALTNDKRRMEADIAAMQSDLDDALNAQRAAEERADRLQAEVNRLADELRQEQENYKNAESLRKQLEIEIREITVRLEEAEAFAQREGKRQIAKLQARIRDLENELEADQRRLREAAASARKFERQWKEVVQQADDDRRQVAELTSLTDQLTMKCKTYKRMIEEAEDVANITMNKYRKAQSMIDEAEQRADMAEKNLTAVRRSRSMSVSREVTRVVRV

>Unigene23530_2A 39 641 AGAP003319-PA [Anopheles gambiae str. PEST]

KMNARLRIIAVHVCLVSWIYQTTDSCSCFPTHPQNQICRDGFVMVAKIVSRKEAPGSAGPFPVGQIEYTVRVIIPVKNSPTHGSTFKFTTADNGAACGVSYPIGSIQLLMGNNNRIGLCDSVFAKSLPQLSYIFSKGKRTSYLRNCDCKIYDGGVFPKPSAKQSCKAPSDLNTKACYVNQGLCVKIGRGQRTKCVWKNIKR

>Unigene23533_2A 1 501 AGAP003319-PA [Anopheles gambiae str. PEST]

PTHPQNIVCSNGFVMVAQILSRQKAPGSSPFPAGEIEYTVRVVIPVKNSPSHGSTFKFTTADNSAACGVSYTIGSVQLLMGNNKRIGICDGLFAKSLPQLSYIFSRKKDTSYLKNCDCKIDDGGVSPKLSAKQTCKTPSNQNTKACYANQGLCVKVGRGRRSGCVWK

>Unigene23600_2A 2 199 PREDICTED: leukocyte elastase inhibitor A-like [Ornithorhynchus anatinus]

MYVSQARHKVVIKVNEVGTEAAAATVIVVTRRSLPRAEFFANHPFVFYIRHVGTSSILFIGHFNGP

>Unigene23611_2A 150 947 proximal thread matrix protein 1b [Mytilus edulis]

YQPVIVPVKHKVTEHSHGYNDGTCDAKADIMFLFDDSSSITADDKENPTRMKNFMKTIVTKFNGVGPGGTRFGVVMFSATVTEQFPLTKYQNKEDILKAIDKIQPTNGGQTAIGAGLQYVSENSFTSSGDGTKRQKIVILLTDGKNNAQPPPLTWANKLKGENVTIIGIGVGSDFTTGDAKGELEQLASHKDYVLTSTSFTQLKTLIDQVVQMACEVCEVDCDGNADIAFLFDDSSSISEDSKENYPKMKEFIKAAMGRFDRIGED

>Unigene23623_2A 85 1191 minus strand ANISERP protein [Anisakis simplex]

IIPTQSLFEEKLISFSHDLYREIDGTRSECLSPFSIISALLMLMAGTGGSSKTQIKSAVLDSVWNDALIYHQYQSSKDHVFSAKENKLYIATRLFVKKGLQVTRNVRNTAKTYFDARIRNLDFKKSKRSARVMNRYVARQTNNKIKDLIDPNWLGSNTIMVLVNALYFRGQWETLFNPKDTKNDVFYLTNGRKVNVDMMELKHTLRYYNGRSFEAIALPYEGKRYEMVIVLPRRIYGLKQLKSSFSPSVAHVIDSSFVNTGLLVKIPKFSFESETNLKVLLPELGITDIFKPGIANFSKLVKQVKKGMYVSEARHKVFIKVNEFGTEAAAATVFVISKFHPQQDFTANRPFLFYIRHVQTGAVLFIGHY

>Unigene23707_2A 13 1146 PREDICTED: similar to HyTSR1 protein [Ciona intestinalis]

CLVSDWSEWSKPDPSGKRYRTRKVIRPALNNGAKCPDLMQEDNVEVPCIVSEWGPWSYPDDQGLSKRVRTITQAPLHGGTACPPLEENRNVERDCVVTEWTLWSKPNRRGNSERTREVQVKSLHGGKECPPLRETRQVDLDCIVSEWGIWSSPNQNGISRRTRTVLRQPIGNAGACPDLEETKQVAIDCIMGPWSEWSEMIEGGLVFRSRKIRKAGNRFGKECGPTEESKKLAVDCVVGEWGEWTTNQFTGVKSRRRRVKQGNLNGGKPCPRLFETEESVVDCVVSDWSSWGSADRKTGISTRTRRIVHKAVNGGKECPDLIERKKVKIDCIVSEWGPWSLPFGFGTKTRSRTIERAPANGGKPCPALEQEAPATITP

>Unigene23721_2A 223 1212 tyrosinase [Pinctada fucata]

CVNKYLAITWRDRYYQKMSNDPEVINWFKSLYRKNKSLEYHSRLKRQTGSLLHGTRQEIRTLSNAQRRRFITAVQRLKTTRVGSTNRYDAIAAAHDGPALENAHFGPNFLGWHREYLLLFETALRQIDSRVVLPYWDTRLEYNLRNELESNIWDLSFMGDSQGVVSYGPFARWRQPNGAFLERSAGADGSYIPPVGIQRVLSRQSNIQISEPWAADRFSLENYHNAVHMRVGGSMTGLNTAPADPIFFLHHAFIDCVWEDFRRQQTYPERDYPWNNQARRSQRPSERMINLESNKRNIEGYSNEYTQRYYRCARSPQTCRECRNRRNLRC

>Unigene23727_2A 274 1293 minus strand tyrosinase [Chlamys farreri]

LNKHMAKLNAHKDMITPLTDSQKKYLLHLGRKLKGIEYAYRRRKRSTRLPAHFLHKRQEIRVMPEKQRKGYFDAINKLKNRGFYDAISNLHQQTAIEGAHFGCGFLGWHRVFLLILQLAVWDINPTVMLPYCDTRLDYNMNNPRDTILSSEKYFGNFNGIVRSGPFQYWNTPANVQLQRNGFSGGSFITDENIRQTMTKRHTREVCAVPGIFNIEHYHNSVHVIIGGLMGDLNWAPCDPIFFCHHNFIDYVWEQFRIHQRKDEGIDPGNDYPNTDDKWHKPDAKMEMIDRYLNLNLTHKHGYDNMFSDEIMIAWENSPSYPNCGNSPDIIKDDTRKVCHS

>Unigene23797_2A 218 4504 minus strand thioester-containing protein [Euphaedusa tau]

MTALLLTPVVLAACKNTYMVTVPQSIRPGHEFSVCVVPLVRHLGFASMQFTLMNRKNKTITYADKDILLHSDPVVIKIKVPQNVSVGYGYQLDVKSMGGLSFAKRVTDMRMLSKTSSIFVQTDKAAYKPGDLVQFRVFGTNSRLRFLNDPINVILTDPKRNRVDQYLNVKPSYGVFTGSFQLSTLTNLGSWNIKVEQKKTKYTKSFTVEKFVLPKFEMNIVFPPFQMRSDKFFRFDLVAKYTFGKPVEGKVILNITRRWGWNNNDVITKHFKMNGKASIRVLMTEITRFGSYINVKAKLVERLTGKSATDSQSTRIFYRREKLTTSSSMSDYFKPGLDYTIIIRASRQDDAPLENPLGTMNITVWYSVLKPIKKDFNTQEMRIYPGSTEEKILWSKDIQISDAGIAVATAKFPIAAVRARITAEYRKAYAYKYLSRAYSASNSFLQVTIANDAKAKPGRRLPLILETTDKRRCINYKIFAKGSLVTQGKWRCWSKKQRVNIMITNDMAPTAKLLVYYIRGKNGEIVADAITFPIKDIFGVNKVSLKFNKSKVLPGEKVELKIRAEPNSLINILSVDKSVILYKTGNDITLRDVMNELSRYDSRYSRFFWGWNFWFPRRVRGVDAFTIFDQTGLYVLTDALLYQYISDCQGSSSSHFRSSMDSGPMFASAQAGPPGPTSTVARGTAPSTLATPARTRKRFPATWIWANTTTGSDGTAVYKTTAPDTITTWVTSAFAVNPTSGLALATELENITTFQTFFMRFDLPYSAIRGEVVIVLISVFNYVPRDQEVYVTMASNSNFSFVDTNGLNINPGPNGLTKKIRVSSNGIEGVYIPIIPTKVGTITFNAQARSTEAADAVEQKFLVEAEGSPQSYNVPFLINLKTARSFSASVSISFPPNFVKDSEFIKIQVIGDLMGPVLNNIQNLLRMSYGCGEQNLAVFVPSVYVSSYLKATNRLTPELAERAQRYLKAGYQRQLTYSRSDGSFSGYGNRDKSGSTWLTAYVIKAFSQATEFTFIDKNVITRAIQWLLKQQQDKGYFNEPGIVFSKSLQGGSASSTRSLTAFVLIALIEAKQNKQIEVKIQKEVDDAISKATSYVAAGAPSSIRNVYELAISFYALILVKHFSATSLLSALEKRANTEDGQKYWQLPDFDIYKISQWRSWRPPRSRARAVDVEATSYVLLGYNLRDDLDNGIRVLKWLGRQRNPWGGFISTQDTVIALQGLTHLGKKLYSTSTSVTITAKGDTWSGKTFNVNNANALVLQSEDLPKSVRKIEIIARGRGICLLETAVFFNVFKEMRQPAFELKPTVLRDSTRGFVLKMCFRWLRAGQSTMGYLEITLPSGMEADLGSLNTKQTFGQFKKIEKAFRQINLYFDAILSNEMCIEINVDRVSMIARHKPVPCRLSEYYEPSNEVIVMYQSKALANATIIEVC

>Unigene23807_2A 131 1150 minus strand papilin, isoform C [Drosophila melanogaster] >gi|272477220|gb|ACZ95054.1| papilin, isoform C [Drosophila melanogaster]

CKGNDENCPSGQICCGVVCGTSCKRSVCDVPQSVGPCVNSKRRWYYDQKKMRCRKFDKKGCAANKEVNGFESRKKCRKLTKSCVCLLPKQPGTCRGSFPRYYYNSKSGKCENFIYGGCKGNKNNFRTPQACQRRCGSHPPRPSTPPPPPPPPPQGPHKCPSGIPIHDCIIGPGNRPCQTGYYCVRSPIGGPGSCCPIPCKHGSPHKTLSCGMVASSNKCPPGYHCLGGPADEYFGCCPNGPMEKPGKCPVPTGVGICVEQCSKDEDCKGTQKCCSNGCGHTCTEVLPVVKPGFCPPLPPNTVGHCAELCTGDASCPGTQKCCSNGCGHVCSFPTGGIKPP

>Unigene23851_2A 511 2127 minus strand PREDICTED: chorion peroxidase-like [Acyrthosiphon pisum]

CGNDKYRRPDGRCNNKLYPFMGSAGTPQSRFVQPEYGDLWNLGFFPRIKGKNGNILPSPRDISNAVFKPTGQSPKSQKFNIALTHFGQFIDHDVIATPIFAESDGSDIECCNGNSMIFRPECFPFNTPPGDFKYTCMNFVRSLPAATFPWFPGPRQQLNDITSWLDLSSVYGSSNDELNLLRGSNGKLKEGNNGLLPNEPTGINTCEKEGAQNLCFLAGDHRNSEVPLLTVWHTIFMREHNRIADRLKFGGINQKTFQEARKILTAVYQHIVYNEFLPVLLGFSFSNAIGLISRPEGHISLYSPFHDGSTRNAFGAAAFRMGHSLVGNFVGGYNNKQERLQDTFFETAITRDQTDYGPSGIGEWMSENFGRQMDRFISDQLRNHLFETAHGEGLDLGALNVQRGRDHGLPGYNKFREFCGLIPAGFFSNTPGGLVDHTVDAVNKLRSVYSNVDDIDLYAGAISERPKSPDTILGPTFRCLIAVQFLNYKYGDRFFYENDFSRTGFTREQLDDIKKQTLAGIYCRTLKLDKIQPKLFLNP

>Unigene23862_2A 8 220 minus strand PREDICTED: similar to HyTSR1 protein [Ciona intestinalis]

CPDLMQLNKVPLPCLMGPWTPWSKPDATGTIYRVRYVVRPALNGGKECEDLMQLKKVPLPCIVSDWTQWSA

>Unigene23897_2A 21 857 PREDICTED: cubilin-like [Saccoglossus kowalevskii]

CISSEFACADGKQCIPLSKKCDGTPDCNDRSDENCPASQCGVQAIQPDIKHPDCPKFKLWRIVGGCSVRPKHSWPWVLQLILDVNKNGKFEHECGAALISESYAITASHCFLTSKDTNRWKVVAGRHKKVGQEPTHQIRKVSKILSYPNPLNDDYNPLDKDITLIKFDKPMKYTDYVSPVCLPQREPVINDICIVAGWGETQGTGNESVLKNTIMPVVSNDKCNGSDALKNQITKYMFCAGFQEGKNDACQGDSGGPLMCQINNAWHVSGIVSWGIDCA

>Unigene23899_2A 50 358 minus strand PREDICTED: dynein, light chain, roadblock-type 2-like [Oryctolagus cuniculus]

VALKVNTNKMAEVEDTLKRIQSHRGVIGTIVVNCDGIPIRTTLDNSTTVQYAGLITTLTNKARSTVRDIDPQNDLIFLRIRSKKHEIMVAPENEYLLIVIQNP

>Unigene23905_2A 2 2830 minus strand thioester-containing protein-F [Chlamys farreri]

QTDKAAYKPGDLVQYRVVGTNSNLKVLRDPLDIIILDPKNNRVQQHLKVRPVFGVYTDSFQLSTLTNLGEWTIQVEQGKEKTTKTFTVERFVLPRFEVNVIIPSFQLITDRYFAVKISAKYTFGKPIKGQCELKIQQSWTDENAIVKTFPINGIATIRVLMSDIVQLNRYNVRVTATVTEGITGISANDTQLSSLYETPEKLTFSSTMPYNFKPGLPYNIILRVTKRDDSPIDTPLGRVNVTVTYRLPREPSGPISMPGPGLAAPAQSIGIIGPRPPFPPRSHEIVLWSREIQIPKSGVIRATASFPKNAESANIQASYRKANAVNYMSRAQSPSNTYIQVSILNDVQPVPNKYLPLKIDTTEKRVRFVYYKVISKGSLIAQGRWRLSRGKENARLRITYAMTPTAKLLIYYTKRDGEIVADALTFSIDDIFRTQIGIRFNKEKALPGEDVALNIKGKSRSLVSVLAVDKSVLLYKSGNDITVQDVMQDLQEYDGYGFFPRFGGWDFWLPRPIGGTDADSVFRSFGVYVLTDATLYQHRRPFRQFFGLEGPMAAGGVVEDGVQALPAATAGNSNGGGGSNLATPTRTRKNFPQTWIWSNVTIGRNGRAVINTKAPDTITEWITSAFAVHPKTGLGVASEFANLTTFQRFFMRFDLPFSAIRGEQIIVQISVFNFLDTDQEVTVSLSNSNDFMFVDNNGNEITPGSGGLKKTLTVKSNGVNAVYIPIQATAIGKIKLKATARSRAASDSVERDFLIEAEGIKQSYNVPLLIDLRSQNNFKSTIEIDFPPNLVKDSEFIKLQVIGDLLGPTLNGIDDLLRMSYGCGEQNLVGFSTNVYVTSYLHATGRLTNALETKSEKILAAGYQRELSYAHDDGSYSAFGNNDKSGTTWLTAYVIKSFSQASKYTFIDINVMTKAIEWIIKQQQPTGAYNEPGKVLHKAMQGG

>Unigene23921_2A 131 607 minus strand PREDICTED: ganglioside GM2 activator-like [Anolis carolinensis]

SFSYKECDDSTGLVQIKDLSVSPSPIVSPGNLTVSGQVILKTVVTGDLQVSLVVRKKMFFFWLKAPCIASYGSCSYDDICGRLKNAFVYYGTTLCPEPFTMMNMGCTCPIKTGTYHLPPIKFHIPDLHSFLLGDYKVELKLRDMTLDKVLGCYTVNLFL

>Unigene23927_2A 2 283 lysozyme [Cyclina sinensis]

DALNVNSNGSKDYGIFQLNNRYFCDRPNGTSSSICWRVNTYGCADTCASFRNSDITNDANCAVRIKKCGGFGKWYGWRNHCSDVSGSAYNYSNC

>Unigene23933_2A 261 1127 minus strand serine protease inhibitor CFSPI3 [Chlamys farreri]

KPVCGSDGFTYLNICRARQAMTKILCRKECPCPCNCPERKNDVCGSDGKTYRNRCQATRCNKVKVQCKGRCPCKQKRCLSCNRTVQPVCGADGRTYTNECRAKCQKVSIDCQGTCPCSCFRLCPPIRDRVCGVDGKTYTNICFAQRCNKVKVRCKGRCPCGQCASCSNKAIPVCGTDGKTYLNSCRAKCAGKTVSCVGSCPCNGDPVCDRQDCPFPNPVCGTDGKTYKTKCDAKCSNVRIECAGACPCRRCDCPNLKFPVCGRNIRTYDNFCRAGCAQQDVLCVGRCPC

>Unigene23948_2A 128 895 PREDICTED: mannose receptor C type 1-like [Saccoglossus kowalevskii]

KCPSIDGWNRQGSFCFWFSASTDTWFGANRKCKRLGGFLAEPKNSDIDGYISRMGTSKDVMYWIGLSDLKREGKFVWKSSGQAVRYTNWRPGDPNNYKGIEHCGLTNWNKAGEGWVDAPCNWKMNYVCQAKLECAPGWHKKGKSCFWFSDITDTWYGAKEFCRKVGAFLAEPKDAETEKFITNDAIWKRVNSWIGLSDLKREGHFVWESSSESLRFSNWVPGDPNNYNGNEHCVLTNWHRKWADAPCTWRKHFVCQ

>Unigene24012_2A 78 1181 PREDICTED: similar to serine protease inhibitor [Ciona intestinalis]

MTTFEDSLISFSHDIYKEIDGTVNGCVSPYSIISALLMLMAGTRGSSKTQIKSAILNNDLNDQLIYSQYQKLTKDVFSAKDNKLYIATKLYVKKGWKVTKKVRKVAQNVFDAGIDNLDFSKSTESADVINRYIAQRTNNKITDLVKSRWLNSLTVLVLVNALYFKGTWETQFNPENTRKEDFYLTVTNKVKIDIMTMDHTVRFYNGRSLTAVALPFMGKKYEMVFILPKQIDGFNDLKTTFTPSVARIIEAGLKTDFVRIKIPKFSFSSETNLIDILPLLGIIDIFKLGIADFRNLVKDAKKDMYVSQARHKVVIEVNEFGTEAAATTVIVVSRKSLPRVEFIANHPFLFYIRHAETGSILFIGHFNG

>Unigene24015_2A 124 252 minus strand PREDICTED: delta/notch-like EGF repeat containing [Taeniopygia guttata]

QGRHFPGHEDPCYGNPCGDYGTCDHDGVKHWCKCAEGYAGEPC

>Unigene24027_2A 314 463 minus strand Beta-microseminoprotein precursor [Esox lucius]

CEKCNCHNDGVSCCSLFTTPTDFPNDCKLIYDGCKPFVVKKNDETVECPV

>Unigene24103_2A 73 582 minus strand PREDICTED: tissue inhibitor of metalloproteases-like [Nasonia vitripennis]

ILTVSVFVGLLAIVFGCTCFHGHQQDKFCRSQFVIHAVPIAEQVPNPPDQTKDRVYKLVIIEVFKDTSGGLVPYTINQAYTAFHGAACGVYLTKYKSYVLGGRFDSNGNMRLQICQLNKLYSSFEPCELAWMRGFVTYNYQNSYLGHQKKPGYDCNCKVKICFKGQKDCY

>Unigene24116_2A 725 1432 antistasin-like protein [Haliotis discus discus]

MRCPYGFVHDTSGCKLCKCKEPVCPVIKCPHYCPNGYNKGKDNKGCHYCKCKTCPDEVCPKYCPNGYVKGKDSNGCSYCKCKTCPEVKCPKYCPNGFEKEKNSNGCYICGCKPCPPVACPKFCANGYEKEKDANGCYNCGCKLCPPVACPKYCPYGFEKEKDANGCYNCGCKPCGPSSCPTYCPGGYIKGKDSKGCKYCKCKTCPSATCPIYCPNGVIRGKDNKGCDFCKCKPCPP

>Unigene24117_2A 11 904 antistasin-like protein [Haliotis discus discus]

CPPASCPLDCPNGLIRGKDNNGCDTCGCKPCPKVSCPADCPNGVIKEQERFQCEICKCKPCPLVVCPADCPNGVIKEHKPYHCETCKCKPCPLVACPHYCPNGVIKGKDSNGCPYCKCRTCALLKCVSCPNGYIKDSNGCDTCKCKPYLCRGVSCTIHCPYGYKKDSHGCKTCKCLPPVCPASACPTYCPYGKTEGKDAKGCDYCVCKVCPQVSCPSTCQYGYDKGKDNKGCKYCKCRPKPPCPQVKCLVPCPYGYVKDSNGCQTCKCESYQCPLVKCARHCPYGFKKNSHGCQTCDC

>Unigene24118_2A 5 202 Cysteine-rich motor neuron 1 protein [Cricetulus griseus]

KPCPPASCPKYCPHGVIEGKDNNGCDICTCKDCPSTTCPIYCPNGVIRGKDSKGCDFCKCKPCPPA

>Unigene24159_2A 229 1110 papilin, isoform C [Drosophila melanogaster] >gi|272477220|gb|ACZ95054.1| papilin, isoform C [Drosophila melanogaster]

WFNDECKSNDANCPKGQICCGHTCGTTCSIPVCDQPKSVISCKIGPPRTKSLWYYDKNMMRCRKFKADECSGNNNNFSSRKSCNKVKATCVCLLPKVPGPCRGSFQRFFYNSLTDKCEQFIYGGCKGNKNNFKTLRKCKRRCGEHPPQPPTPPPPPPPLKCQKGIPLEITCGLGPGSKSCPTGYYCVFHRRSSAGFCCQVPCKYGSPHKTLSCGMVVGSNKCPKGYYCEAHPADQYFACCPNGPGNKPGICPVPTGAGACVEQCSKDGDCVGTQKCCSNGCGHTCTNAVPETKP

>Unigene24304_2A 335 922 minus strand GF19243 [Drosophila ananassae] >gi|190618374|gb|EDV33898.1| GF19243 [Drosophila ananassae]

GRFRCGSQNNALSKTRQLCDGKKKCTIFSSNSIFQDPCPGTSVYLTVEYECDCGPRFFKCKNENKCINKRLVCNGKRNCNDGSDEVNCPTKGCRPPRFQCKGSRKCIPKRKRCNGRYDCPDGSDEAFCECTNRQFKCKKSGQCIPKNRKCDGNRDCPDGSDEIGCGCTSSQFACADGQQCIPKKKVCDKYPDCKDG

>Unigene24330_2A 100 1587 protein disulfide isomerase [Haliotis discus discus]

NMLPILLSACVIALTFGADIKEEEGVLVLTTANIEDALKENTNILVEFYAPWCGHCKALAPEYAKAAKALADEGSDIKLAKVDATVESSLAEKYEVRGYPTIKFFRDGKAIDYSGGRQSADIVNWLKKKTGPPCLALESVDDAKKMVEKDEVVVIGFFKDLKSADAQQYEKAAQGIDDIPFGITSNTDLFKEYEMESDGIALFKKFDEGRNNFEGSVTAEAVNAFVMANRLPLVIEFTQESAQKIFGGEIKNHILMFLEKTADGSDKLIDGYKKAAETFKGKVLFITLDTSDEDNARILEFFGLKKEDCPSARLITLGEDMTKYKPESNDLSEDAVRSFVQNFLDGKLKPHLMSEEIPSDWDAKPVKVLVGKNFQDVAMNKDKAVLVEFYAPWCGHCKQLAPIWDELGEKYKDSADIVIAKMDSTANEVEDVKVQSFPTIKYFPKGSSEVVDYNGERTLDGFVKFLESGGKDGAGEAEDEDDDDEEEDEDEPAAKD

>Unigene24349_2A 279 485 minus strand PREDICTED: oikosin-like protein-like [Saccoglossus kowalevskii]

ECSTDYDCKGVDKCCSLACDCRKSCVPPGDCLYNRKAYLIGETFPAKDGCNACTCMPDKTTSCTEKACN

>Unigene24388_2A 460 1707 fibropellin Ib [Strongylocentrotus purpuratus]

CISKPCQNGGICTEEAKGYRCTCRPGFSGFQCQIDNTIKKYCSSKPCKNGGVCKEGVRGYTCICKPGLTGKQCQANINECVSKPCKNGGICKDKINDYTCKCKPGYTGKQCQTDIDDCVSKPCKNDGTCKDGINMYTCICKQGFTGKQCQSNLNLCTKTSCMNGGICKNGIGGFTCTCKPGFTGKLCHVDIDECASKPCLNDGLCQDEVNSFKCICKRGYTGATCKTNINDCIGKPCKNGGICKDAIDDFTCKCKPGFTGKKCQTDIDDCISKPCKNGGICKDGINSFSCICKSFYTGKVCEIAPKGCVSMPCQNGGVCKDEPNGHKCICKPGFVGANCQTGMTCKNKPCENAGVCKNVVDGYLCTCKPGFTGVNCQIDIDDCAAKPCQNGGVCKDQINGYKCTCKPGFVGVNCDK

>Unigene24400_2A 6 395 minus strand PREDICTED: serine (or cysteine) proteinase inhibitor, clade B (ovalbumin), member 1-like [Saccoglossus kowalevskii]

MVIVLPKKLDGLKDLKTSLSPFMANLIESNFVNTSVIISIPKFTFEAETNLKVQLPKLGINDIFIEGKADFSNLVKEKNDIYVSEARHKVVIEVNEQGTEAAAGTVVVTLFRTTPTEPTFTANHPFLFYI

>Unigene24504_2A 236 358 minus strand RecName: Full=BPTI/Kunitz domain-containing protein

AAKAADVCQLPKKPGPCRGYVPSYYYDIQTKSCQKFIYGGC

>Unigene24528_2A 225 782 PREDICTED: collagen alpha-1(XIV) chain-like, partial [Amphimedon queenslandica]

LDIVFVLDMSGSIGKANFEKMKESVISICNTYTIGSNAVQVGVDVFNNRAVTKIRLNQHNNINSLISAIKKIRYRGGGTKTYLALLNLLCKSFTRRYGDRSNAPNLAIIMTDGNSDDKEKTLAAAKLVRMFGISISVIPIGENIDMSEITGMTGGTSGLVIPALNFNTLLTKAFQQKVSSLKCSAA

>Unigene24624_2A 114 1466 minus strand PREDICTED: GL12416-like [Saccoglossus kowalevskii]

MRECISIHVGQAGVQIGNACWELYCLEHGIQPDGQMPSDKTIGGGDDSFNTFFSETGAGKHVPRAVFVDLEPTVVDEVRTGTYRQLFHPEQLITGKEDAANNYARGHYTIGKEIVDLVLDRIRKLADQCTGLQGFLIFHSFGGGTGSGFTSLLMERLSVDYGKKSKLEFAIYPAPQVSTAVVEPYNSILTTHTTLEHSDCAFMVDNEAIYDICRRNLDIERPTYTNLNRLIGQIVSSITASLRFDGALNVDLTEFQTNLVPYPRIHFPLATYAPVISAEKAYHEQLSVAEITNACFEPANQMVKCDPRHGKYMACCMLYRGDVVPKDVNAAIATIKTKRTIQFVDWCPTGFKVGINYQPPTVVPGGDLAKVQRAVCMLSNTTAIAEAWARLDHKFDLMYAKRAFVHWYVGEGMEEGEFSEAREDLAALEKDYEEVGVDSVEGEGEEEGEEY

>Unigene24729_2A 726 2540 Peroxidasin [Acromyrmex echinatior]

PVPRDSCKPGYPYRVPDGTCNNPDETVTKGASIIAQPRILPNAYDDGKGTPRSKSKIPGRFLKSPRLISNVVLSTARNNRSPKSNRFTLTLVHFAQFVDHDIIFTPNSGIPKIGENGDIYLGKIENCDENCIDPGQTHPECFPIPLPEGDDNFQNEFKSSCLHFVRRSPMHGDILCTDGIREQRNDRTSFVDLSSTYGSSKRRIDDLREQNGGRLNASKGDLLPFGPKVDDCKSTVQQCFNSGDDRHQEIPMLTVIHVNFLREHNRIAGELEQNNNWDDEKLFQETKKILTGIYQHIVYNEYLPAILGYFHSYWFGLMSSNYGHRNIYNSNVDPSTMNEFGVAAYRFGHSLVGRFVNALNQNSQPVLQNGDLLKHDFFDNGKIRDKDFGPEGIGRWMAKTSMRKSDRFISEQLRNHLFENVTTTESNGGKSDGFDLAAFNIHRGRDHGIPGYNEYRKWCGLQPAYVFDNGFWGFRDHDSQTVKKLKQVYDHPDDVDLFVGGISEKPQYPFPYFSGILGPTFSCIIANQFRKYKEGDRFFYENPFPDTGFTLDQVNDIKRQTFAGIYCRSLGIEYIQKNVFEKISLQNPLVRCDSLPKPNLQLWKE

>Unigene24747_2A 123 1520 minus strand sulfatase 1 precursor [Helix pomatia]

PNIVFVLVDDFGYNDIGYHGSEIKTPNLDRLARNGVILENYYVQPICTPTRSCLLSGRYQIHTGLQHGNIMPSVPKALPTDSYTLADKLQQAGYNTHCVGKWHLGFYKEEFMPMNRGFDTFYGFLVGSKSHYHHRRCHKGMCGHDFRENGTPVNTKRVYSTKLFSDKVVDIIQNHKSRKPLFIYLPYQAPHSPVDAPRKYVNLYPNIQSRERRVFAGMVSAIDEGVGNITKALKDQGLWENTILIFSSDNGGQVIRGGSNYPLRGNKGGNFEGGIRGVGFVSGGYMRNRGRISTELMHVTDWFPTFINLGKGNYIGVKPLDGVDQWKAINGGRSQRRVLLHGIDPLARKVGKPWASDTFDSRTSAAIRIGDMKLLTGKTGSREYNPNTVTGQKRQTPNEPRNKNVWLFNITADPLETRDLSSSHPVIVRELLNRLADFDRTSISPQEARSDPRADPDRQGGFWGPW

>Unigene24756_2A 2 868 minus strand thioester-containing protein-F [Chlamys farreri]

KAMQGGSASSQRSLACFVLIALEEARRNNQVVPKINDSVQRSIDSATKFIADGAPATTDNLYELAIMFYTLSLVNHNVKDQLLIELERKSQTAGGGKYWKLTDTVANKVQPWRSWRPPRTNARAIDVEMTSYVLLGYNIRSDTPNGILVLRWLGNQRNPNGGFISTQDTVIALQALSGLGVKLFVKTFSMTVGTQAATWKGRTFTVDDGNALVIQNEDLPKTVRSISIDAKGRGLCLLEAAVYYNVFEELREPAFDLTPTVMKETINGFTLRACFRWLRGGESTMGLLE

>Unigene24801_2A 161 484 alpha-2-macroglobulin [Branchiostoma belcheri tsingtauense]

TYIATVPQQIRPGEDAEICIIAIRPVRRQVNIGVTLMDKDNKTIQSTRTVRFLRQRPITVRLKVPLTITQQDSYSIRVKGSGGLNFDATARFISVSRKISSIFIQTDK

>Unigene24814_2A 38 505 PREDICTED: similar to trefoil factor [Ciona intestinalis]

SSELSVSDSTGNVEDLRPTSKTPFESSQPTLSVEYTPSEERPIKEVKLVSTDNIKSFTVKFTAPDGTVTTKQVPVNEVATNVGTEPDVKKVEIIITPQDKIDAYKPVKLQVQIHACFELESTTTATTPVSSVSKKVTSTTAPPSTSSTGTASTTPL

>Unigene24838_2A 1 300 PREDICTED: similar to HyTSR1 protein [Ciona intestinalis]

GGKECPDLLQSRKVKLPCIVSDWTEWSKPDTTGTIYRYRMVTRPSLNGGAECPDLLQSKKVELPCIVSDWTEWTKPDSTGTSTRSRYVIRPPLNHDGECP

>Unigene24840_2A 4 312 PREDICTED: similar to HyTSR1 protein [Ciona intestinalis]

CIVSEWTGWSKPDATGTRYRFRMVLRPALNGAKECPDLLQMRKVPLPCLMSDWTAWSAPDATGTIYRVRYVLRPALNGGKECEDLMQLKKVPLPCIMSEWTQW

>Unigene24841_2A 5 244 PREDICTED: similar to HyTSR1 protein [Ciona intestinalis]

NGGKECPDLIQSRKVPLPCIVTEWTRWSAPDASGTRYRVRYMVRPALNGGKECEDLMQLKKVPLPCIMSEWTQWTKPDAT

>Unigene24896_2A 390 965 minus strand PREDICTED: similar to metalloproteinase inhibitor 3 [Tribolium castaneum]

ILLLLSMVKISEECGFPGPPFVIHPQNYFCDAKYRMTGKVISSDQANLQKIYKVKVLTVMNGFKQSDIGKEVNIYTPSSSAACGITLTVGKIYALGANKKDGKLVIRMFMFYYEYFNMTVQQHMYMMNKHGKLSYKANCRCKVSADTSDRTCVPGENASCYINNAICSYNRQRDKCNWINGDTCUDLLVEYL

>Unigene24906_2A 52 4323 minus strand thioester-containing protein [Chlamys farreri]

ILKCMATSCNHFITVPKSVTAGSELDISITVMKKGSQSMTMLAILKNSASKGISGASRNVRGKSGKTETLKIKIPDDTPPGSSYQVEISVSGWIFLHKTTYNVVIQKEKSIILIETDRPIYKPGDLVQFRTFGIDTDLKPLDNPLNITIFDSSGNRVKQYTNVMDKFGMFGDSLQLSMVTNLGNWKIDVRQASHRDSVSFEVAEYELPKFEVKVVLAGIPHIMDDFFKIRVTAIYTFGKPVEGYATIKIKRRWSSTDILVHKTIKILGKVDIKISTWDLYPVYGTYFDVNAVVNETVTGKNDTDFMSFDLKSSKWRVTFSPTMTLMKPGLKYDILFRIEDINGKPVKSKGIAKLIVGYDYTIPYKRSTLRKKGILVQKNLKLGPTGEVDYGVLFPPNTTSARIYVIYEKDNIGNKYIGKATSPSENFIQIRTKGIHTVQVDHLLTLILSVTENIRLCNYKILSSGVTVKEGIFRMNRRKTRKQKVYITRDMIPAARILVYYIRHDGEIVADAITFPVSDIFENKVTISFDKDKAEPGQRVNLRLTADPGSLLNVAAVDKSILLLKDANDIDADEVLSEVTSGDFFTQRLSKVWGRTVTTPETGSTANTVFQNSQLNVITDCELVGEITRQPFRGHPGAARTIGTTHTGLLSTLRTSVAATEALQSSLSPTAPVEENLVTPSRKRSNFQETWLWMNTTASSNGVSTLGVVVPDTITQWIASSYAINPLTGLGISLEKANITTFQRFFIRIELPYSVIRGETLILKISVFNYMETDQMVHVSMKKNENLTFVDENGNTIPVQGDSRLKLVNVPKDSIRSVYFPIIPTRIGKTSLDVTAQTTEVADAVKRNLLVKPEGISQKYNLPVAIDLREERRFSTSVKITFPSNVIPDSEFIKVSATGDLLGSTLDGIEDLLKMSYGCGEQNMLNFVPNVFVSEYLNVTNRFDKEIEKKVKKILADGYQRQLSYSRYDGSYSAFGKSDRRGSTWLTAFVLKSFAQAAKFTYIDPNVMQRATQFLINQQRSGQFTESGRVLHKAMQGGSAASVASLTAYAVIALSETLRHQQIPKHMVVRTVGAIRSGVDFIANSLVAKKNTTREMYELVICTYALTLQKALLANTLVKDIEKMANYSDGQMFWVLPDNEVDRLQPYTFWRPPSKQYRALDIEVTAYMLLIYNIRNEISKGVQVMRWLNDRRNPYGGFVSTQDTVIAIQAISGLAERIYSPKFDALLTTYGKDWTGATFNVNNENALVLQSVDVPPTTKEITFTGVGTGFLFSEVSVFFHVSEELRKPSFSLSLSILNDSILGFGLRICFRWLRGKQSTMGYLEVTKPTGMEINQETVNVTESRDQFKKKENSGNQLNMYFDAITEKIMCVEVDFERISLVVKQKAASVRLSEYYEQSNEVIKMYQSRMLARASLADVCEKGQC

>Unigene24924_2A 8 340 PREDICTED: similar to HyTSR1 protein [Ciona intestinalis]

WSAPDATGTSFRWRMVTRPSLNGGKECPDLIQSRKIPLPCLVSDWTTWTAPDATGTTFRWRFVTRPAINHDGECPPLIQSKKIPLPCIVTDWNEWSAIDATGTTFRYRMVT

>Unigene24925_2A 4 303 PREDICTED: similar to HyTSR1 protein [Ciona intestinalis]

RYRMVTRPSINNGKECPDLIQSKKVPLPCLVSDWSQWTAIDATGTIFRYRFVTRPPINHDGECPPLIQSKKIPLPCIVTDWNEWSAIDATGTTFRYRMVT

>Unigene24937_2A 411 722 lipopolysaccharide binding protein-like protein [Lymnaea stagnalis]

TNFGIRLSANWNINFKIVFVRIKASGTLNVSAKGIDIILTLDKKTFKVKTCKDSIKTLDVKFEGKDLAGNVISFLANTFKRPVSRRLKKELKGKICKAVTKLIN

>Unigene24939_2A 281 1258 minus strand predicted protein [Naegleria gruberi] >gi|284086956|gb|EFC40629.1| predicted protein [Naegleria gruberi]

VPKCKQYRCINGVVHTLYSGCVFKDLSGTETCYAVDQRWNEGCSSRVCSYVETIHDGRKAHMYVLDGQPKCQAQNKKCKKSGDVWEWECQKYTCKEKDLLYMKVANVERIGKVDNCFCCNYKGKCYLNGTAWAGENCTVLSCGLHYSAETGKHVAAIAVIPGCDYGGKCYDYGSKIKQGCCVKECKQKAGTMDVFWHTVSSGCSYGGKCLNPTEFVRNASECLQYTCNSDGSITSKVYGCEYKNKCLPFGESGFDDECYFKKCFLKDGIAVLDTLRGACMGINNMCYEYGDTGFSKEIHGKIYHNCKCEQKGKTGSEVTCEAPQSY

>Unigene24951_2A 226 1578 minus strand extracellular matrix protein [Dictyostelium fasciculatum]

SQSCTKDGKTRLDGSEWKEGCTVFTCKAGHIDHELKGCYYSDTDTCREFDQVWGDPNDPCVEKGCKLVDEYSVTEYFTKKGCQKSDGTCVDVGKQFEENCSVYECVEGDNTLGREVVSQGCQFKDEDKCFKDGESVKRKCSTYRCERGHAIPDLKCSHGENCYAINSTWTEGDFEYTCKFNGTNLILSPRRTSDEPAVVVTGCKHNGIEKELGDKWTDGCFEMECIEAPNGDFVTKILSGGCEKDGQCLQDQEEWTSDCSIFKCVKKAENHYASVVVERKCQVKYENGTVMCVAEGWEGQYKKCVKQTCVVEGNRWKLKLDIACPVLDTDKTEMCVQDGWTGKHPEKRCVFQRCITNATGYVVAYDIGCPWNKNECVTLNDTRIHRCTSFMCKKTEKGYKMEKIGKAGCYVPGGYESFSETESIRDNCVNVNSLLKKYKKNKCLVYQCQQS

>Unigene24983_2A 186 755 PREDICTED: collagen alpha-1(XII) chain [Rattus norvegicus]

SGGMDLVFLLDTSGSIGRRHFQKMLDLVIGITSRLAIGQYATQVGVEVFSTRAATEIRLKDHNNIYTLRSAIRKIRYRGGMTNTYLGLDRLRLRSFTSYYGDRWRYPNVAIVMTDGNSQYKKKTLNAAKLVRLYGIEIIAIGIGDIIDWDEIDGIGGKAFSRQASKALRAESFSTLVTKSFQKLVFSRVC

>Unigene24985_2A 141 635 minus strand oocyte protease inhibitor-2 [Oncorhynchus mykiss] >gi|38049039|gb|AAO16105.2| oocyte protease inhibitor-2 [Oncorhynchus mykiss]

VFLLSTAGEAFYYNETPCQRELRINTNNAAPGARIPVCDKEGNYAPKQCSGSQCYCVTRVSGTTIPGYSVNRWEAQDQNCQCARDKVENTVPGKIFLCDKMGNYKETPCQKEYRENTEATNNGAMGARIPKCGPDGHYDPKQCSGSQCYCVTRIQGKMIPGYSIN

>Unigene25030_2A 34 2400 minus strand precollagen-P [Mytilus galloprovincialis]

MVGIQLGLIFLVAVSSKVLANPVGYGIVPKVGYGGGGGVGIGSASAQAAANAAARASGGGFGIGGGSAGASSNANANANANGGGFGGVGGGSARASSAANANANANGGGFGLGGGSAGASSSSSANANAQGGGFGGGSAFGSANSNANAFGSGGFGTGSASASAAAQAAARAAAAAHYIGLPGVPGEPGPQGPPGPPGHRGPAGTRGAPGVTPPGHPGEPGQPGQPGQDGAPGAPGIPGKAGPPGRPGAPGIPGKAGEKGEQGALGESGQPGHPGSPGLSGRPGTPGNPGTPGRVGDKGARGNPGAPGLPGIPGEAGAAGGIGPRGPRGPPGLIGLLGPRGNPGALGSVGLPGEPGAVGPTGPQGPAGIPGFPGEDGDKGEQGSVGVQGPRGVPGSPGSQGPPGNQGDIGDTGNTGNAGTPGLPGNKGADGDVGPQGPQGAQGLQGPNGGAGVKGQTGDQGARGSEGGAGEPGPPGVMGLKGPTGAQGPPGLPGPTGMQGPSGEAGSQGPPGREGPSGPLGQRGAVGPQGPPGERGAQGAPGKNGQPGPRGQQGAQGAPGKPGARGERGPKGSTGSPGRAGAKGKRGEAGPQGPVGPRGVKGPNGAPGSVGPSGPPGVIHLVVEDLRNEPVVQYAQAQAAARAHAHAIAGGGSAAAHAHAHANAAASGGAGGVGGGSAAANAAANAAARASGIGALGFNLGGASAGANAGANANAAGGGGGASGGSAHSQASASGGNQNIYRTQSFNTGNSQGSTISHVQTYGSGSSASSSSSSQASSSSGGGIYKADY

>Unigene25031_2A 34 2190 minus strand precollagen-P [Mytilus galloprovincialis]

MVGIQLGLIFLVAVSSKVLANPVGYGIVPKVGYGGGGGVGIGSASAQAAANAAARASGGGFGIGGGSAGASSNANANANANGGGFGGVGGGSARASSAANANANANGGGFGLGGGSAGASSSSSANANAQGGGFGGGSASAAAQAAAQAAAASPVIGYPGLPGAPGPRGPPGPQGHRGPAGTRGAPGITPPGIPGDAGQPGQPGQDGAPGAPGIPGKPGSQGRPGAPGIPGKAGAKGEQGDLGKAGQPGEPGSQGLAGRPGTPGNPGIPGRVGDQGPRGDIGARGEPGIPGESGSAGAVGVRGPRGPQGLMGLTGIGGNQGSSGSVGLPGEPGAVGPIGLQGPVGIAGFPGEDGDEGEEGPAGLQGPSGVQGSPGATGPAGNQGDIGDTGKTGADGIPGLPGNQGPVGEIGPQGPQGPQGVQGPNGRLGISGIGGDQGSRGPEGVAGQPGPVGMKGLKGPIGDQGPPGLSGPTGREGPSGEAGSQGAPGNEGPIGPMGPTGAVGPQGPIGERGAQGGPGKNGESGARGSQGATGAPGTPGARGDRGPAGSIGSPGRAGAQGKVGEAGPQGPVGPRGPKGPTGSPGIVGPAGPPGAINFVVEQLRKESAIQYSQASAHAHARAQAIAGGGSAAAHAHAHAGASASGSGGAGGGSAAANAAANAAARAAGGFGALGFNLGSASAGANAGANANAAGGGGGGSASSSASSRAHASSSASGGV

>Unigene25068_2A 145 1272 RecName: Full=Actin, adductor muscle; Flags: Precursor >gi|1373222|gb|AAB02227.1| actin [Placopecten magellanicus]

MCDDEVAALVVDNGSGMCKAGFAGDDAPRAVFPSIVGRPRHQGVMVGMGQKDSYVGDEAQSKRGILTLKYPIEHGIVTNWDDMEKIWHHTFYNELRVAPEEHPVLLTEAPLNPKANREKMTQIMFETFNAPAMYVAIQAVLSLYASGRTTGIVLDSGDGVTHTVPIYEGYALPHAILRLDLAGRDLTDYLMKILTERGYSFTTTAEREIVRDIKEKLCYVALDFEQEMATAASSSSLEKSYELPDGQVITIGNERFRCPESLFQPSFLGMESAGIHETTYNSIMKCDVDIRKDLYANTVLSGGTTMFPGIADRMQKEITALAPSTMKIKIIAPPERKYSVWIGGSILASLSTFQQMWISKQEYDESGPSIVHRKCF

>Unigene25120_2A 481 906 minus strand PREDICTED: eppin-like [Equus caballus]

CPNIDLFCKGYADYCKSDKECSTGTVCCYSRNCGRSCQKPLVTDVCKLPKVVGPCDGVFIRYYWDTNTKSCRKFEYGGCGGNGNNFLTKAQCEQKCGPKCGPVCLIFCPFGNVLDDNDCPICKCKTGCRNNVPPLKNMSCQD

>Unigene25185_2A 132 3161 thioester-containing protein [Chlamys farreri]

YRKEKAYKYLSRARSLSNSYLQVSIVNNKRPVTNRYLQLKIRTTESNVRYAFYKVNSKGSLVTKGRFRLYRHDRIVWLKITQAMAPSAKLLVYYTRRSGEIVADAITFTVDDIFKNRVSIRFSKRKASPGEKVRLNIHASAKSMVCVLAVDKSVLLFRGGNDISVQDVMKELLQYDTSMHVPIFRRWDFWYPRSFSGIDAYSVFYLFGVYVLTDANLYKYRPPPRHLLPPTRFGGEKHGMAGQDGGSAPDMAFVDQNTPINPDLVTPTRTRKHFPETWLWKNAFTGLSGRTQISSIAPDTITEWVTSAFAVSPTTGLGVASEFANLTTFQRFFMRFDLPYSAIRGEQIIVQISVFNYLATEQRVVVTLSNSVDFIFVDYNGNALPTGSNGISKTIIVKTNSVGAVYIPIEATAIGKIKLHATARCEAAADAVEKDFLIEAEGIKQSYNMPLIIDLKTTSIFNAVIDIDFPPNLVGDSEFIKLQVIGDLLGSTLNGIEDLLRMSYGCGEQNMIGFVANVYVSTYLKATERLNLDLEAKAKKVIAAGYQRQLTYAHNDGSYSAFGNNDKSGSTWLTAYVIKSFSQASKFVYIDKDHVMKRALVWITKQQEHNGTFDEPGKVLHKAMQGGSVSSKRSLASFVLIALEEAKRNNQVSKSILSSVNLAILKAAKFIAHDAPASIGNLYELAISFYALSLVNHSKKDELLIDLENKAKTDGGGKYWKLTGPVAQKVQPWRRWNPPRTNARAIDVEITSYVLLGYNVKGDTLNGIKVLRWLGNQRNPRGGFISTQDTVIALQALSGLGVKLYVKTFSMTVKTKGDIWSGKAFKINDKNALVLQSEDLPKSVRSISISAHGTGLCLLEAAVYYNVYDELRIPAFDLIPTVLKETINGFTLKVCFRWKRGGESTMSLLEINIPSGMEADTDSVDASEAHGQYTTIEKAFRKVNLFFDAILSIQMCIRIDIRRTSMVARHKPQSIRLSEYYEPSNEVIKTYISNKLANATIIEVCGSDNC

>Unigene25272_2A 71 1993 neurofilament protein NF70 [Helix aspersa]

ITEKRTVITSSSSNYDDGDDSIYYKSGIQPRSSTVVNRSSIGPSMRASVGGGSGGTVYTRTVEYGMGRSSGLGNLSPGSYEKVSNTGVMTVKSSREKEKKDMQDLNERFANYIEKVRFLEAQNRKLAGELEHLKTKWGKETSAIKSMYEQELAEARKLIDDLTRDKNKLEIQNSSLQEEMNGLRRQMDDLKKYHALDQEQINKLNQQLSDYESEINMLRRTISSLETERARDKDRINKLQGEVDRLRIDLNNETLNHLDAENRRQTLEEEMEFLKKVHEQELKELAALAYRDTTEENREFWKSELSQAIRDIQNEYDNKVDQIRGDMESYYNLKVQEFRTGATKQNMEVTHVKEENKKLVKSISDLKGRLADLEARNAQLEAQYNSLLREYESMQSEHTMETVKLKEEITNLRAEMEAILVELQSLMDAKLSLELEIAAYRKLLESEESRSHIIERIRGAGFYRRYNDENFPIDMIDTVDVDIPNDSVQPETRDPDEPPDSDSKPEDSHSSMKMMRGEVSAKTTYQKTSTGPVSIAEVNPEGKFITLENTSSQRREVNLDGWKIRRELDGQREVVYTFRNFTLKPHKSVKIFARGSAQEAGINDLVFRDEETWGVGSQVSTCLVNEKGEEKATHRQRTAYN

>Unigene25277_2A 64 501 minus strand GF17533 [Drosophila ananassae] >gi|190626021|gb|EDV41545.1| GF17533 [Drosophila ananassae]

VSIFCIIIQSVLLLWIIRSTEACSCLSGHPQTLVCRNSFVMVAKILSRIQGLPSGGSTFGIFKYTVRVILPIKNSPKHGSNYDFTTPDDEGLCGVSYPIGSIQLLTGSKPNSISLCETLFKPTLPQLSYIFSRGKARSYLRNCECQ

>Unigene25284_2A 140 658 minus strand RecName: Full=Metalloproteinase inhibitor 3; AltName: Full=Tissue inhibitor of metalloproteinases 3; Short=TIMP-3; Flags: Precursor >gi|4588552|gb|AAD26150.1|AF110767_1 metalloproteinase inhibitor 3 precursor [Scyliorhinus torazame]

LAIISIITTVTFACNCTEKILQEHFCEDDFVLSAHVLDNEDAPDSDNGDRRYKIWILQKMKGLDDKMIMSEDYLYTKPTQQSCGVILQMLRTYVVSGKIIDDKMWINLCGLYYFGSDLPCPLEVYTNGCKAECQICEANETCDDIQRSCFYQSKNLHTIYSNACIPADSGCTW

>Unigene25302_2A 418 1269 minus strand CRE-TYR-3 protein [Caenorhabditis remanei] >gi|308250293|gb|EFO94245.1| CRE-TYR-3 protein [Caenorhabditis remanei]

VGRSTPKGPRQRREYRTLTEEERNTYHRAINKLKEDTSVPPNKYDALAQYHSNTQDSAHGGVAFAGWHRYYLVLYERALQEKEPSVMLPYLDTTLDIEMTDPSDSVIFTDKFLGNPHGQVTTGPFGHWKDKIERSVQKTTQLRVKREDIEQFIKESSDQGEFVRVLENAHNTPHVWVGGGMLDLDRAPFDPIFYMHHAFVDCVWELYREHAKSNGENPEKYPPTDNDLHVGNRSMVNLPPLNGVSLTNSDGYKNSWTQVYYTCAPLPSCSDSHRNCGSDWLECD

>Unigene25318_2A 372 812 minus strand PREDICTED: papilin-like [Nasonia vitripennis]

DGKGCSKFINRCKMNDANCHGGKICCPTECETRCVNPSPCALPKDPGPCRANIRRWYVSSSGWCRVFRYGGCQGNVNNFKTRRQCIRNTRHCRCLLAPDEGSPCRAKLPRYYYSKIKRKCVKFYYGGCKGNANNFKKMKICERICGV

>Unigene25362_2A 3 341 sporozoite cysteine-rich protein, possible [Cryptosporidium parvum]

TRYRVRYTLRPALNGGKECPDLIQTSKVPLPCIASDWSAWSAPDATGTRFRYRYTVRPALNGGEVCDDLIQVTKVPLPCIMGPWTPWTKPDATGTVYRYRYVIRPALNGGKEC

>Unigene25469_2A 3 140 minus strand thrombin inhibitor haemalin [Haemaphysalis longicornis]

PGPCKGYFPRYYYDYTRKGCLQFVYGGCKGNGNNFQTLKECQRRCG

>Unigene25517_2A 65 823 thioredoxin peroxidase 1 [Haliotis discus discus]

VLISICFTLTFSQQALSADDGQCYTYGGDRQCYASPGGHVYPQETRRTSGHAIQWSQAVISKPAPDWNGTAVINGEIKDIKLADFRGKWIVFFFYPLDFTFVCPTEIIAFSDRAVEFQAINTQVIACSVDSQFTHLAWINKPRNEGGLGPIKIPLLSDITHEISKAYGVYLQDLGHSLRGLFIIDPKGILRQITMNDLPVGRSVDETLRLVQAFQYTDKHGEVCPAGWKPGSDTIIPDPKESQKYFSKQKNAN

>Unigene25573_2A 162 875 minus strand papilin, isoform F [Drosophila melanogaster] >gi|272477222|gb|AAF56794.4| papilin, isoform F [Drosophila melanogaster]

ENSCELPKKKGTCNARIERWFFNINKGSCRKFIYSGCDGNPNNFESKNDCKKACNARVCRLPKNEGQCNDTRSKWFYDIDEMRCKQFTYTGCKGNKNRFSSKNKCLKRCGGVKKNACPPPTPCAAPRPDDCVTPRYIFVDGVRCASGCLYPPCKSGNCPPPLPPQKCVQCQYSRDCSGDQLCCSSCCRIPVLDPKGGSCPSIGLRRCIGPSSECSLDSQCTGKRKCCSIDNLKCCVDP

>Unigene25605_2A 128 412 theromacin [Hyriopsis cumingii]

MAPKALVTIIIFLLVAFCIDQYKCDWITECWPPMTSCSGMAPVSGEMKWTTCRRRCRCKRYYTGSCRYNTNQCPNSTPYYCRCYGVWNGELPSYC

>Unigene25617_2A 100 4290 minus strand thioester-containing protein-D [Chlamys farreri]

VVGYVSCKNTYVATLPQRIRPGENAEVCIAAIGKVTSTVSIVVTLLDKDTKVIQSTTTTKFIRLRPVTVQVKIPLKIDQTGVYSMRVKASGGLNFDETAKNIRVHWKSTSIFIQTDKAAYKPGDLVQYRVFGTTSKLKVLKDPLDIFITDPKRNRVQQHLKVQPLFGVYTDSFQLSTLTNLGNWNIEVEQGIEKTSKSFFVERFVLPRFEVNVIMPSFQLTTERFFTVRISAKYTFGKPVQGDCELKIGPDRNAIVKKFKINGTARIRVLMSEINLLKTDNIRVLATVTEGITGISANDTAYTWLTSTPEQLRFSSSMGGTFKPKLPYNIHLRVTKRDNSPLDPPLGRVKVTVTYSPRGKPGVPSSSSNNEVLWSRNIQIPNSGVITTTALFPARADSGLIIAKYRKGRTRRFLLRARSPSENYVQVSIVNDKKPKPTQDIPLKILATQRNIPWVYYKVTSKGGLVDRGRFRLRRGNGNVNLRITYAMAPSAKLLVFYTRSNGEIIADAITFTVDDIFKTKVDINFNKKRALPGEDVRLNIQSSARSLVNVLAVDKSVLLFKTGNDITVQDVMRELQSYGNGFFPIFRSWDFWFPRPIYGVDAYSIFRNFGVYVLTDATLYKYIPPPSRLTDANLGQKGVEGAGGGSAGGGGAGGARIRKHFPETWIWANVTTGPGGSAVINTKAPDTITEWVASAFAVNPRNGLGVASEYSNLTTFQRFFMRFDLPYSAIRGEQIIVQISVFNYLATQQQVVVTLSSSSEYDFVDTNGNPINPGPGGLSKTITVQSNGVGAVYIPIQATAIGKIKLKATARSTAATDAVEREFLIEAEGIKQSYNVPLLIDLRSQNSFKSTIDISFPPNLVKDSEFIKIQVIGDLLGPTLNGIDDLLRMSYGCGEQNLIGFSTNVYVSTYLQATGRFTPELQSKSQRILAAGYQRELAYAHDDGSYSAFGNNDKSGTTWLTSYVIKSFAQASKFTFIDINVMTKAINWVVLTQNTSGAFKEPGKVLHKAMQGGSASSERSLACFVLIALEEARTNGQVAPELMTKVQNAIDLATTFIVKDAPASIGNLYELAIAYYAMSLVKHSIASSLLFELEKNAKTADGGKYWELTGPVAQKVSPWIRWSPPKTKARAIDVEMTSYVLLGYNIKGDTPNGIKVLHWLGSQRNPRGGFISTQDTVIALQALSGLGVKLFVKTFSMSVNTQAATWTGRTFNVNNDNALVLQSEDLPKTVRSISIDATGEGLCLLEAAVFYNVFEELRKPAFALIPTVLKETINGFTLRACFRWLRGGKSTMGLLEIKIPSGMEADTDSIDTSGAGDQFTSIEKAFRQVNLFFDAILSTEMCITIKIIRTSMVAKHKPQSIRLSEYYEPSNEVIKTYISKKLADATIIEVCGPDNC

>Unigene25658_2A 130 684 minus strand tissue inhibitor of metalloproteinase TIMP [Crassostrea gigas]

FLVITISVVYGCYPVYPRHPQISFCKSQWAIIGTVLDTEYIKENQIGNKKALIRYKVRISEKLKGHKKVTAGHTVSVYTPGNAALCILLLHNNNKYVLGGGKRGRNLFIRDEDLHLDYGKLTQEQKTYFSRGRNSYKRNCNCKVTMHDTTTSCGVPDKSPKHDCFAERGLCKMNRYGKCHWYKNK

>Unigene25687_2A 144 377 PREDICTED: Notch homolog Scalloped wings-like [Saccoglossus kowalevskii]

GWNPPSDPCDTNPCGDGAQCNNQGDTYTCTCDARYRYDGTKCVTWPCYGNPCGEFGSCRYEGSKYWCECIDGYKGKPC

>Unigene25692_2A 151 714 PREDICTED: similar to collagen type VI alpha 6 [Ciona intestinalis]

LDIVFLLDMSGSIGSRHFRTMLEATIGISNSLTIGPFATQVGVDVFSTGVTTEIRLKDHNNINTLRSAIRRIRYLSGMTNTFLGLDNLRLRSFTPRNGDRRGYPNVAIVMTDGNSQNKKKTLNAAKLARLYGIKIIAIGIGNNIDWDEIDGIDGDAFSSQASKKAMRAKTFSTLLSKSFQQDLTRRIC

>Unigene25716_2A 395 1453 minus strand CBN-TYR-2 protein [Caenorhabditis brenneri]

RQEIRMMSPQRRKNLRAAWKKAYDDGYFGWLARFHNDQIRDSAHAGPAFPGYHRFFLLMLESVLKHYDKDITMPYWDSTKEANMENPANSVLWTDDYLGEMNGVVNSGMCGGFRDLRGNPIMRNGGNAGSLFTSYDTRFVINIPNIERLTEPSPYVTLESAHDNVHNWVGGTMAPIELAPWDCIFWMHHAYVDYLWEVWRYNHNYDMKYPYKAGLPGHGPNTPMKNMPYMRFLGRVPTNKDGYSARLAKLAYYQLSPTCSAQNTYCGSPDLQCKIGRYASECVSVDVRFRRETQPFRQANNRGGNVNFKVNRAGRKKRRADHQGYYEDYEFSVPADQIDNIHIPIEREPCMGR

>Unigene25721_2A 143 559 minus strand PREDICTED: metalloproteinase inhibitor 1-like [Sus scrofa]

ILVKFSEECSIPPPYILYPQNLFCSAKYVMVGEVLSSNVGTTLYRGYRVRVLAVIKGFKASNVGKVKKIFSPKSSAACGTTLTIGKIIVLQATMDNDGRLIIRTFMFRREYSKMTVQQHMYIWLKRGKFSYQANCRCSV

>Unigene25766_2A 229 573 minus strand phosphatidylethanolamine-binding protein 4 [Macaca mulatta]

PSIKFSSAQKDKKYMMLMRDPDAGDASDVLHWLLTDISGEDLQAGVIDKNINEGYWGPNPPNGQHHRYQFMLFEQKVSPVNESKKKRIGFNTLAFLKDNQLCPSDAVASFQFKSY

>Unigene25769_2A 221 481 minus strand secretory protein [Xenopus laevis]

CNVPPIYREECGWGGISPETCEARGCCFDSSISGTKWCFKKARPECWVLPTYRKECGWPGISRQTCEAKGCCFNDNISGVKWCFHTK

>Unigene25834_2A 4 471 proximal thread matrix protein 1 [Mytilus galloprovincialis]

NGTQFAAVLFSDRVREHFGFGDFTNLKDLHDAIDRMNTSNGGATAIGDGLDYIATNTFKKIRGGEVQPIVILLTDGKNNAGYKNPETAAAELKKKYKAQIVVIGVGNKYEKSELMNIATDPSYVFETATFDGLAKILDDVVSMACEVCAVHGVRHG

>Unigene25995_2A 72 1442 minus strand precollagen-D [Mytilus californianus]

MVHQILTVCLVASLIQHGLAAAIGYKSKPIYGAGRGAGAGLGGGYGDGLGAGNLYGAGGHAVSSSHSHSHSSSHSSSRSHSSSFSHSSAGGFGVGGGAGGASAAAAAAARAAAIANGGLGGAGGSASASAAARAAALANGGLGGIGGIGGGGASAGAGANAGASAGGVGPGPFGPGAPGTPGGPGGPGGPGGPGGPGGPGGPGDFGPGGVGPGGPGGFGPGGFGPGAFGPGASAGGLGSATAFGPGGSANAAARALAQSGGFGLGAQGPQGHPGLPGPRGPPGPSGPQGPAGPHGAPGITPPGQPGPPGEPGAQGHPGAPGAPGAAGTPGVQGPAGPVGRPGRPGSRGESGKPGTPGNAGAQGHAGAGGEPGQPGRPGAPGQPGEQGPTGNRGRPGAPGQPGRAGAAGAVGSPGPRGIAGIIGLPGPHGDLGPQGPIGHPGLPGAPGPMGPQGPAGP

>Unigene26000_2A 3 239 minus strand precollagen D [Mytilus edulis]

PQGPAGPSGPKGPQGDTGAPGPAGKQGPAGPQGPAGPRGPSGDQGPVGEKGAPGAQGPGGRPGDAGLDGSVGPQGPAGP

>Unigene26028_2A 264 629 minus strand C-type LECtin family member (clec-42) [Caenorhabditis elegans] >gi|3876045|emb|CAB04128.1| C. elegans protein F16H6.1, partially confirmed by transcript evidence [Caenorhabditis elegans]

CKISNDPNAKIFLFNGKYYAFSTILLNHDQAQAACKAGCGNLVAINSVEEQAFIVSRTTAGGDWYIGLTKGSGNWHWTDGSAITYNNYGNNPVNAATCGYLDQINKWMRVTTCNVSHRFICE

>Unigene26029_2A 200 1882 minus strand PREDICTED: collagen alpha-1(VII) chain-like [Xenopus (Silurana) tropicalis]

DMVFLFDDSSSISHNSPGNFQIMKNFMKKIVNSFETVGPSGAQFGAVMFSDRVDGPFHLNTFSTKAAITQGVQDIIPKEGGSTAIGAGIEYVRLNSFLPANGGGRDEVAKIVILLTDGKNNGQPPPIDEALKLKNQDVTIIVIGVGSDVEMNELRQIASRDDYVFHPDTFAELNSILDSVLSMACDVCENGMNPHGPPGPPGPPGPKGERGDPGEPGPPGKIGPKGPRGDKGPVGERGVPGMPGDKGGIGDQGPRGEPGRDGRPGLSGQKGEPGEPGAPGQQGRKGEQGNRGEQGYRGLPGYRGEPGEKGKSGDPGLNGQPGIPGAKGERGENGKPGRDGIPGMKGEKGEPGVQGPMGDRGEPGYHGAPGRPGQKGEMGDKGMTGEQGVQGINGEDGENGVPGTDGIPGNKGAHGDRGAPGTDGLNGIPGSKGVKGDRGDKGPSGDPGGRGDPGKPGEPGTNGENGRDGIPGLNGMNGKPGLKGAKGDRGDEGPMGMIGMSGLKGQPGEPGTPGRPGVPGNAGKDGDDGIPGMKGERGDKGLTGDRGAPGIPGKDGTIRVI

>Unigene26137_2A 261 602 papilin [Rhipicephalus microplus]

LATNTRVGVCNLPKDQGPCKMRLIRWYVTPFGRCRHFIYGGCRGNANNFKTRVLCYWSTRRCRCRLPKNPGPCKAKIRRFYYSKTQRKCVKFTYGGCGGNANNFRSRLRCQRTC

>Unigene26138_2A 876 1871 minus strand Unknown (protein for IMAGE:7008304) [Xenopus laevis]

YISVKEMNIDLLSALSHDNDLSGVDWSRGKLAYYVLGLLATCQKPNDFYGHDLVTILKNNLVQQSSYLNSNKFAYALIVIALCKSGENVSDSVLNNISTTEGQYTFGVDEAAMVYLAYRCTNNSSFIDKENAAIQLILASQDPHGTFGNEYSTALALQVIFAGGNASLLDRTNKSINFLAKSIDKMNNPFTSLLLSVLPPLAKKSLLDVGNVQCPPEPTPTVPSGPVISTQIRVTNVVTASNFDQSWIASLTHGQTLYDAMVNLALASQGFSFDSKTTTWGQFITSINGMVASSDERQYWSILDFEGTPLSKGVSETFPTFGDSYQFNMTTW

>Unigene26148_2A 201 1379 PREDICTED: tout-velu-like [Saccoglossus kowalevskii]

KIYIYNLPPALNADIVKELLNTDLISKGMMTTSGFGQEVFKTGKYDDISVRNTYQFALEIIMHQKMMASPYRTLDPDEADIFYVPAYTALRCIVKDKDCQSCLFNRYLSKLVSFLHQQPYFKKKRPHFSTIGKIHRDLANFDCPYLLYPVMKEINFFGIEKVLEEDMGENLLVNQAAQSIIVVPYPSYVHFTGKTASVTNFKYDRTVFALLPVAHSEESSISAKLANQFNGTTSMSYGKYFQDKKRGMYDVVHISTEGKTDDVKIRKTISWMTRSIFCLIPPGDSSTRREFYDAVLSGCIPVLFIKREIPYAFANSLDYSKFSVAIKQSDILDGMKVTDILKTVSPSVIKQLQYNLDLVSQRLQFSVMKANVPDAVDIALIELERLIKSQEKS

>Unigene33183_2A 157 1095 minus strand antitrypsin isoform 3 [Bombyx mori]

DTNITRNLTTVGSPNGQSVVISPYSFSAILMLLMMGANGRTKSQMSSAFFPRHRRHHFKPNHLFRMLDSVLPPPRKGIGFNLQNEIFIDRKCILNETLEQEAKKYFDVTIRRKNFHHNPIGSVAGINRWIKRYTKRKFDNLITPDMITKNTIAVFLNAMTFNMKWKKKFHPVTGAQDFYVSKNDTVPLTMIRSIQKARYGKFNAFEVLVLPLKGKQLNLVVILPENPETLPEFEAMVTMDALHYLKKSTKFNNIDITLPKLFLKNKINLKDYLPKIGVKRIFNTIENDFNNLFKGHKRIYVSGAVHNVVTKMN

>Unigene36167_2A 31 939 minus strand serine protease inhibitor 2 [Bombyx mori] >gi|7341330|gb|AAF61252.1|AF242200_1 serpin-2 [Bombyx mori]

NDALIYYQYRALTGKVFSSTNNELYIATKLFVKQGLSVSSYVRYIAKSYFNADIGYKDFSKAHQSAIDINKYVASKTKNKIKKLISSTMLNDATVMVLVNAMYFKGTWEKQFDPANTRKRYFYRTINNRVKVDMMAMQDYVRFYNGMDYSAIALPYSGGAFEMVIVLPSQIDGFNDLKNSFSPGVANYIESQFVNTNVSINVPKFTFESETDLLDLLPKLGINDIFGTSADFSALIQGQYRNIYVSEAIHKVFIEVNEIGTTAAAATAVVFTYGSPAFIANHPFLFYIRHKPTGIVLFIGHYK

>Unigene42355_2A 1 468 minus strand PREDICTED: superoxide dismutase-like, partial [Saccoglossus kowalevskii]

NPTSITVTSCDQDNQVNCKVGDLSSKHSNLTVGEKKDDSISSYVDTNLPLDGTNSIVGNILVLRATNGTFLTCSKIVQYTYREVSAVFSRDGVKGHIQFSQLSPFDETIVMLNLNGLQNLAGGYHVHKWPVPEKWASNQNVCSDNSVSGHFNPFEI

>Unigene44680_2A 42 1019 serpin 3 [Ctenocephalides felis]

NACVSPYSIISALLMLMVGTHGTSKSQIKTSILKIDLNDDFIYQQYQSLSGQIFRTTGNDVNIATKLFVKNGLTVTADVRNVATTYFDADIGNQDFTNAISSAKDMNNYVASKTNNKITDLVKSNWLNINTVMVLVNAVYFRGIWETQFDPKNTQKEDFFLTNGNKIKVDMMSMKHYIRYYNGGKYSAIALPYKGKQYEVVIVKPSSFNGLRDLKRLFSPALAKVIDSRLSNTTLLVKIPKFTFEAETDLKVILPKLGITDIFAPSRADFSNLVQEVQTSMYVSEAHHKVVIEVNELGTEAAAGSAIVVTRKSQLQEFVANHPVLF

>Unigene48643_2A 32 259 serpin peptidase inhibitor, clade C, member 1 [Xenopus laevis] >gi|18140917|gb|AAL60467.1|AF411693_1 antithrombin [Xenopus laevis] >gi|27370856|gb|AAH41211.1| Serpinc1-prov protein [Xenopus laevis]

VSIYLPKFKFESGTDLKILLPKIGIVDIFDVGNADFSNLINGHKDGIYVSEARHKVVLEVNELGTEAAATTVIVTG

>Unigene57547_2A 51 464 minus strand Metalloproteinase inhibitor 3 [Harpegnathos saltator]

LSLLLLSSHIVFGCRPAYPIHPQNRFCQSDRAIIGKMFSVTESRGEVRYTVQIIKVLKGYPRHIAGQKRYVFTTADSASCKISLEVGKTYVLGGSKKGKHMSVISLGLFLEVPKLTWKQHIYLNTRYGPYSYRRHCKC

>Unigene58614_2A 673 2604 minus strand PREDICTED: circularly permutated Ras protein 1-like [Amphimedon queenslandica]

KRHSWRKVRSRNLYDNKPQAASGRTVKQADTNVVSIDFKQLVTPGHMFTGDPVYCTGCKALLSHISKINKIGNQQVWACEFCGTTNNVHVLDGEIPDDSDIMFMLEPAPSKAAIAEPSEGDNSVVIFCVDVSSSMNDTTEIPGKIEWVNALSVNRSSSWRSTGYDSYRNTTSISRLMGVQAAVLHQLEDMIKNHPNRRVTLITFGNEVSIMEVGRSKPKIVTGETLYKKNKLMDIGKELKLAKAVKDTGKELKEKVQSLYAAGCTALGPALLISVSMAAKCPGSKVILCTDGWANEGVGKVDIHSDEGERFYEEVGTEAAKQGTSISVISFKGTDCKMVHLGKMADQTEGQVNIVDPMKLTEEFSSILAGQITATNAEATIILHKQLYVCNEDMKESRIQKQIGNVTDATVLTFEYGIRASQKIEDNQVTMKIGRKKTNKMEEETSKTTAEISDDFKTKPEIPAELPFQLQMKYTNKEGETLLRVFTHFKPSTKDRMEAEKSTDIDVIGAYVQKQVASMVLDGSYTDARVKALTGQRLVWRYKQIAANKRDRKERKKKYKKIFKNISKLEHRIKHEQKREIEKIGRNLSESEGSDDENDGSSRYRGRSRSRERLQKKKQMRHKVMSDGFSHAVYRSRQARSLSR

>Unigene61287_2A 239 811 intermediate filament [Myxicola infundibulum]

RPYQSLKFNAFTSPRNISVQDYSYTKEVPRSYTPLITSQSEYKSSGTSYSYSPGLSSDFTVSKVGRLEDKEELQVLNNRLSEYISRVRQLWEQRGQIDSSAFLKSTRILEEEIQNLKGMYECELENLRRKLQDNVNERNSLQTKYGDCHHSVKDLEKRLAQEIDKNREMVANVTNLQETISYLEQEIAKLR

>Unigene61623_2A 9 1142 serpin [Ctenocephalides felis]

FETFLLNMASFTDNLINFSHDIYREIDGSQNACVSPFSVISALLMLMAGTGGKSKSQIKLAILGDQLTDILIYRQYKSLSAGIFGAKDNELYIATKLFVSKGLILERNVGYVARTYFKAGIGTLNFANCKRAARIMNRYIARRTNNKIKDLIKQKWIKRNTVMVLVNTFYFKGTWEKQFNPKETRKEDFHLTEQNQVKVDMMQMTHKVRYYNGGSFSAIALPYKGNKYEIIFVLPKRIDGFSELKKSFSPATAKVIDSGFKKTFVSIKIPKFSFAFEANLKLLLPSLGISDIFIDGVADFNNLIQNLKRPMYVSEARHKVVVEVNERGTEAAAATVIVVVFESSPIPQAQFIANHPFMFYIRHEQTGSILFIGHYLGP

>Unigene61943_2A 116 2893 minus strand PREDICTED: similar to centrosome protein 4, partial [Strongylocentrotus purpuratus] >gi|115944266|ref|XP_001189902.1| PREDICTED: similar to centrosome protein 4, partial [Strongylocentrotus purpuratus]

QKALSISIKVDRRDEEIDRLNRMLDGGRPSDVVALEARNRANERMISHLNIQIDFLQQKNRELERKMNETLTMRDEYESENSKLRLKSRQLEAKNKELEYDLGDVDRMAKRLQTDKDLVVKEADREMTEAKDELEKSRHELEDLDQVVAELKAANETLSRQQSELKQQLSVKNGDLLRLEELLDRVTEDKKRISHRVNKLMANEKELVLEIERLKKKNGPATTKKGKTSSKLDAFIRSIEEERNYYRDQADALQKMLRGEIPTRSRSPVRSRSSSRAGSPIREAAGTPTSKTDKKTVAQYETVIRVLEEEKEYYKKEYEMIKATKKSMSSARATPTKILGEDPEVAKLIRERDEMKALLDKFERHMAEIQANVKVLTAERDKLSTMYDETKEELQRVRRELVRSPKSPKTSLAAQAILRRVENERDDAMSDLRRMTTERDSLRERLKIATESSLSDRAKLEQKIEDLENVLHNAETEKNELVIRVTSLREDVRHYEDQVKDQALRLGHTQDEASQQRSTATQMKLLAEETEKSLEDTQKRLNRRDLAVQEQEEKILQLEERIAELNRALLNSKDEAGQQRLTISSLDREKDNLQLAIDEKAEKVARLNEELLTKEKMISDLKIRCNELEAQLEHAGDNIGMKEREIKSLQRQCDSTTEDLSETSRSKDIALRENRRLQDDLAVMTRENQKLNQELQDALDDKEQLRNQVQDYMTEVKRTEDLLSRKEQERSDLLEQYRALSVEAEQYQTSTHQLESEGSNLRLELMTKDSEIRRNRDKIDNLEREIQEHLNAQQAYELQVSNLTRSVGNLEENLRQLEEEKQNLLVDLTAVRELCAKLEGSKESLQRQLTSSSLDREQLQHLVDDMRQETDLLKNQVSAERTNLKNLEGLLQNNREKDFQSQLNQQEKNAEIQMLKDRLSLNESKI

>Unigene62001_2A 68 1645 pernin precursor [Perna canaliculus]

MKLLMLSLVIFAALALQVRADGQCTPNTSSKNHDDPHDDNHKDDQHGDDHHDDDHHDDDETMHYAQCEMEPNPHMASNLHHHVHGSIELSQKGHGAVYLEVHLVGFNTSEDHADHHHGLHLHMLGDMSAGCDSIGDLYNAHPEKHANPGDLGDLVDDDRGVVNEVHHYDWLDIDGTAPNTEALIGHSMTILQGSHKDPDTPASRIACCVIGHGKARPKTAAALHHELEEDKTEHYAHCDVRSNTHQPKALHHHVHGTIDMKQVGYGDLEVTYHLEGFNVSDDYKDHLHDVQIYTNGDLTSGCDNLGAKYDPHEDYHSDLGDLGDIHDDDYGVVNESHRYSWINIFGDDSVLGRSIAIHQRDHLHTSAKIACCVIGRGQSHPEIVHKAKCVVRPNTESTGLHHHVTGNITFEQTPGGATHMTADLTGFNVSEDLSHHRHGVQLHEWGDMSNGCHSLGRMYHGHDDPHAPKRPGDLGDVIDDSNGDVHATRTLDHINVEDLNARSLVIMQGGHEVESERVACCVIGRA

>Unigene62002_2A 31 1191 minus strand PREDICTED: acid ceramidase-like [Xenopus (Silurana) tropicalis]

LLLVVLSACAMIWGQVPPYDETCVTNTYPPSDKSHLVSNYVVNLDLPPVQRWQKLAKDKSTQIKALLTSVINLLDNFGNGTKVQSIIDYLNSKGDSMDDTLPQPYADEMRGIAKASGLGLGEVIIYNIFYEIFTVCTSIVTEDKNGKLYHARNLDFGLFMGWDIKNHTWAVTEVLRPLIVNVDYQRGGKTLYKAVHYAGYIGVLTGIKPGVFTLSMNERFELKGGFIGVIEWIIGIRTGQWMGFLTRDTLHYSNSYEEAKKNLTITKMLAPAYFILGGNSSGQGCVITRDSDKAADVWTMDKAEGWYILETNYDHWKNPLFLDDRRTPANKCMKKMTQDNAGWSGLFNVLSSKPVLNKLTTYTALMQVDAGALETWLQFCPDPCWPF

>Unigene62556_2A 743 1888 minus strand ADAM family mig-17 [Ascaris suum]

GKARRKRATGTYIVEIFIIIDKTIYDFWKKEERNSDNMAMLRIRQYYTFLMFGVDFRFRNMRAVYRMRINLVGIYIAKSNTDLTFINKYRRCSGGRRCKLDANRALSDMRGWIITKRIPKHDHIMAFTRDEMLSGYGDTSGTAYVNSMCTSRSTSIVEDQFDFIAATIAAHEIGHSLGAVHDGDNNRCSRSPGYIMVSVNQAMRRPYHRSPWEFSKCSVEEIGLILDDLDRSKRPNCLRRRHPNSRSYNIGSETRLGQTFNVDKQCELMQGPRSKACRDFLVNTEEVCTGLWCRSTTTSRGEVCIMSLPAEGTSCGHNKWCVGYECRFDSRAPRINAGCIFGDRPGTILRGQTCSNLIRREPWQCYSRSIRKNCCESCYRIK

>CL1167.Contig1_2A 2 337 minus strand PREDICTED: similar to HyTSR1 protein [Ciona intestinalis]

GKECPDLIQMGRVPLPCIMSEWTPWTKPDATGTIYRYRYVLRPALNKGKECEDLLQLKKVPLPCLMSDWTKWTQPDASGTIYRYRYVIRPSLNGGKECEDLLQLKKVPLPCI

>CL1.Contig21_2A 164 1246 minus strand lospin 7 [Amblyomma americanum]

DSLISFSYDIYREIGNDGSTCVSPYSIISALLLLMAGTAGNSKSQIKSAILKNKWDDHVIYDEYGILKDYISYGTKGELYVATKLFVMNGLTVTTGVRDVAKGTFDADISFKDFRKPKISAHHMNRYISKRTNEKIKDLISWKWLNDNTIMVLANAVYFKGTWEREFYPKNTRKADFFLKNTKTEKLQVDMMAMKSFVNLYDGKNYSAITLPYKGGSFEMMIVLPKEIEGLKDLQMSFSPDDAKHIDKNLKNVSAVIKIPKFTFSAATNLKTLLPKVGIKDIFKEGVANFSNLVNEVNRRIYVSEARHKVVIDVNEKGTEAAGATALVVERRSKHQPRFVADHPFMFYIRHGSTGTILFMG

>CL2.Contig3_2A 192 428 minus strand unknown protein 9 [Haliotis diversicolor]

SSSRSIFGSPGYRVRYSVTVHGNAGTLVCCKGYSPATRSYVYIGCGHSSFSGIVNWGNNLATPKIKCKGIPFGASVSWS

>CL37.Contig2_2A 53 1186 GK20759 [Drosophila willistoni] >gi|194157458|gb|EDW72359.1| GK20759 [Drosophila willistoni]

LPMASRTIQSSFEDSLISFSHDLYNLTDGTTSVCMSPYSIISALLMLMIGTRGTSKTQIKTAIFNNTMNDDEIYEQYKSLSSKVFGVPDFELHIATKLFIMNGFTITDDVRRVAREYFSADIGRKDFSKPKQAAFHMNNYVAKRTNNKIKDLISPSWLNANTVMVLINAIYFKGTWETEFDPKDTKKQKFYLSSKKTINVDMMAMNHYMRYCNLLDFTAIALQYKGKQYEMVIVLPKKLDGLHDLKKSFSPDIANQIESNFVNTSVIIRIPKFTFGSETNLKIQLPKLGISDIFVDNKADFSNLVKERNDIYVSEARHKVVIEVNEQGTEAAAGTVIVTVFKSESTQPRFTADHPFLFYIRHRVTGSILFIGHYNPPE

>CL121.Contig1_2A 3 1859 minus strand precollagen-NG [Mytilus californianus]

GGGVGPGGVGGGVGPGGVGGGVGPGGGDGGVGPGGVAPGGPFGPGGVIGGVGPGGSVASGFGQGSAVAGPGGVSANALAGASAGVIPGQPGAIGEPGLPGDQGLRGPPGIPGPPGHHGPPGPPGHSPPGPPGATGRPGAPGSPGTQGAKGRPGSPGPQGPAGHPGADGQKGARGPPGPAGNNGQPGNNGPDGAPGQPGHPGQGGPQGPPGETGPDGFPGPQGQHGSAGAYGEIGPMGPVGPRGIQGRPGCKGRPGIAGVRGVPGPAGKQGPDGLQGPMGKMGPIGGKGPRGDRGPVGDIGPEGPEGKPGLVGPKGAVGPKGAKGAPGDDGEPGSDGAPGERGAEGPPGAAGPKGNAGPEGFEGPAGDKGAPGEGGAAGGPGGHGPQGPGGEQGDTGDTGPAGPPGPTGPAGPKGIRGPQGERGPDGKEGPAGAEGAKGKAGPKGPRGDTGQPGEEGKQGAPGAPGSRGAPGIGGPKGPRGFRGQRGKIGRDGRGGRTGRPGPRGPQGPQGVQGDQGAEGLSGPQGNGGVSGTVRIVVDNNGARIVSMDISGPFGSPELNRQAALDALGPIDLLFTGGAGGFPGGPFGPGGAGGPFGPGGAGGPFGPGGAGGPFGPGGAG

>CL809.Contig1_2A 146 1483 minus strand beta-tubulin [Saccostrea kegaki]

MREIVHLQAGQCGNQIGAKFWEVISDEHGIDPTGTYHGDSDLQLERINVYYNEATGGKYVPRAILVDLEPGTMDSVRSGPFGQIFRPDNFVFGQSGAGNNWAKGHYTEGAELVDSVLDVVRKEAESCDCLQGFQLTHSLGGGTGSGMGTLLISKIREEYPDRIMNTFSVVPSPKVSDTVVEPYNATLSVHQLVENTDETYCIDNEALYDICFRTLKLTTPTYGDLNHLVSATMSGVTTCLRFPGQLNADLRKLAVNMVPFPRLHFFMPGFAPLTSRGSQQYRALTVPELTQQMFDAKNMMAACDPRHGRYLTVAAMFRGRMSMKEVDEQMLNVQNKNSSYFVEWIPNNVKTAVCDIPPRGLKMSATFVGNSTAIQELFKRISEQFTAMFRRKAFLHWYTGEGMDEMEFTEAESNMNDLVSEYQQYQDATAEEEGEFEEEEGEGEEA

>CL8.Contig1_2A 9 311 minus strand PREDICTED: similar to HyTSR1 protein [Ciona intestinalis]

RVRYTVRPALNGGKECPDLIQSKKLPLPCIVSDWNEWSAIDATGTSFRYRYVTRPPININGECPPLIQSRKVPLPCIVSDWVQWTAPDATGTTFRWRFVTR

>CL8.Contig2_2A 2 232 minus strand PREDICTED: similar to HyTSR1 protein [Ciona intestinalis]

RVRYMTRPALNGGKECPDLIQLGKVPLPCIVSDWTEWSAPDASGTRYRVRYMTRPALNGGKECPDLLQLKKVPLPCI

>CL251.Contig1_2A 155 415 PREDICTED: similar to antistasin [Hydra magnipapillata]

PICGPVCLKYCPFGNVLDSNGCSTCVCKKGPKCGPVCAKYCPFGFVLDSNGCPTCECKKGPKCGPVCAKYCPFGFVLDSNGCPTCEC

>Unigene2041_2A 1 214 LEN=214; minus strand; translated

XWNNVSRPIGDQFTQSDCIEYRESFAFVIVREFNMYYCVIDKPVNSTIRGYWKSQEISKYNKQNYCELCDMN

>Unigene16041_2A 1 399 LEN=653; minus strand; translated

VRIIMFGIKSLLVMIGFIALLKWNWLDACRCPDPAKSKIHMQNIFCTTEYILVGKVLNRTQVSRDEKDPQAKYFVKILNSIRGKLRVGSVFDFLDPYNGTPCERNYSVGAVHFLMLDKKKIVDPCGPRKPTSA

>Unigene23300_2A 1 481 LEN=762; translated

XPELQKMLRPFILLSLMATAFAAKKTFLAKAPESEKGPPSLKCIPRKERPFADIKDGGLKGSYPKAAISKAKKSDKSFKSGFSAARIDLCGCYRRPRPPIDLAGVAGPKGPDGPSSPPSPPSPIPFGFFKDTCPAVRSVFTFPRSQFYLGERCWVLRPWAG

>Unigene24029_2A 1 511 LEN=511; translated

XITDGMESPTFIPSSELSVSDKTGNIENLRPTSDTPFESSEPTLSIVYTPSEERPVKEVKLVSTDNIKSFTVKFTNPDGSVTTKQVPVDEVATSVGTRPFVKKVEIIVTPEDKIDAYKPVKLQIQIHACFEVESTTTVTTAVSSTSEKVSSTATPPGITTTGTASTTPVCX

>Unigene24038_2A 1 639 LEN=639; minus strand; translated

LSETYTKYLTMVNIGYILAVVLLWVSTTQSACPCQYPDLQTKFCKSDFAFVGRITSSKPTTDSTSDIEYTAKVIRRIKGRIKVETFVFQSPRPNEDCGVEFPLNSIQFLMGKRLGLPIPSKKFNKITSCEVDPKERPLTPPQWTYVLSRATRNSYVRNCDCEVTTSRDKPGDFCRLVGDGKLRTCYIKNSLCTRIGFANRCGYTNTQPCGGPQ

>Unigene24602_2A 1 639 LEN=809; translated

HDATNLLPASTSTFTGDTLSGKLKIPEFERSCVGEPTQNKFNVDCDIGTPLWSFLPFKIIHLRHGEIKYPSYSVDINGNIINAADVYSIGQQLTFATNTQAYIRANKRDCKFDKSGLTSILVSSYGLNYEGIYQDNVFVDNRRTLSSEISYIGIKTPRNGTTKVFLTATDECGIACQPMVQRRRKPLRYDPFPGAVEISTNGHSYHSQTTRGA

>Unigene24820_2A 1 408 LEN=460; minus strand; translated

SLQTAKMFLAAFVLLMLVNEAYSHIGKGAFGDEILYDKSSQAKCTEVTRQATRDCRWPSGLNRKMDQVAAGGTIIAFKIQWFNGAWSEWYVPGYNDMDGKFNLNTKHCSVPYIKNSLRRMWSYFYDHTHKFIICKP

>Unigene24843_2A 1 307 LEN=307; minus strand; translated

XDTTTYVPAPSTDTSSGTDKTYQTGSLTSEVVSTSGDTETVKQTQTDTSGSSKVKMNKHGVVEYVADSSTDTTKYEPYSPPAESYSPPSIDTYSPPADTYSPP

>Unigene24973_2A 180 953 LEN=1108; translated

VTKIVEAEGECVDKKRCEEYENPCREPFLDWARMYCPKYCNFCIDENGTIGSVTDKVLKPSGQGNTDVIVKPKRKESSNRVSGSSKCKTANGVIDNGERSTDKYRCQHICEDGMLIPDNFGCKFRGKCYRPDETWTYGCSEYRCEINYEPITVKYDAKLTKNNEGCQVDGSDICYGNGERWKTDCIEYSCVVTVKQQENVTRNQTQGQKDVKNDVVIVDRECRDYYGNCRKPGEKFTFKCFGKRCHNCSCELIDGKIS

>Unigene25292_2A 1 220 LEN=220; minus strand; translated

XYVIRPALNGGKKCDEDLLDTKKVPLPCIMSEWTAWSKPDATGTVYRVRYVIRPGLNGGKECEDMLQLKKVPLP

>Unigene25713_2A 1 970 LEN=1777; minus strand; translated

XSVLVIGPDQHTATDFSXELTGYTVKARMDGLKVFSILFAVISVVYGVCDRQAVAKCLEILSKLAPEEGKAPDMQAICGAYSELQTCLSPLREACKDDVTWASVTGMETSYGPMCGGGGAGTGGAGTDGTAGCSMLTVGTCLQPMKGLENLGGFPTSAQMNTLCGTTMTFKDCIKPYKESCDKSQDETIMSLSKSINAIYSLADLFCMKHKSNFIKYAGECFDKGTYATESLKCNKPPPTDSAGKPKAETRDEECSRESANFDCYIQLTKDQCSAEVGNFAESVKSDYMKAMAPLCPTSGAIRLGVQIWIFLAVTMAAVFMYXN

>Unigene25877_2A 1 479 LEN=1053; minus strand; translated

XQYKCRLLLQGVVTLITEFSEIIYQKDPSKVFYSAIMSYISLQDLGKSLECGKTVKFTIKRKAGHDFERGGYAINFVENKNDVHDVQNNVLFHFNPRPNNVLVFNSRYSGHWREERTINNTVYLAETYHLSLHVVNPWVVKIGINGDFYDAYGVYEDITK

>Unigene44441_2A 1 255 LEN=255; minus strand; translated

VKMALMSRTVPVRYDGYWPSRYDRHVPVLRDPIPYDSRFSRFDDLVPYEFERRPLSSSQMWTSFDNEMARINNEMRRNWERSLQS

>Unigene56952_2A 1 493 LEN=493; translated

QRFIDRTIKDRPKLNIVAVMFFVNYLLLVSCIFIAVPNTIQQDGKELAPGDKTAAVAKIKEALTGALESVAIKSIQLRSASYTIQSLVITKHPTITITESNDYYFIWLKNMGVRIRGRWIFFPIVSGSFTATATNVFVHLRYQKKSQSVVYCYVKLRNISVTFT

>Unigene65885_2A 1 259 LEN=259; translated

XSLSSGHTCIGRGRYGDEIIYDRSAQAKCSEITRRPTRDCRWPSGLNLQVDQVAAGGKIIAYKIQWFYGGWSGWYVPGYNDMDGKFX

>Unigene2985_2A 130 405 fibrinogen-related protein [Mytilus galloprovincialis]

GVSFYGTSADRIVDRLLGGTKPWSSKTCNTPRPSDCGELDKTACPSGVYTIYPDDTNGFDVFCEMNRDGGGWTVFQHRENGQVNFYRNWAAY

>Unigene10242_2A 132 1781 peroxidase-like protein [Euprymna scolopes]

CPHHAKYRTFDGSCNNLNHPNWGKSDTLQRRFTDWRGKPMVDFADGKGLPRGYPDKLPDPRRVSNVLSKTTGHNKEDVYRTGQHMAFGQLLTHDILQTVLHSADCCQYPNFDNQDVCYPFNIPKHDDVFQAGCKSFARARRATKGKLPDYRENVNRLSSFIDATFLYGDDLETANYLRVPNSYLMKTDHYDLLPTIDGGSCFKKAKTDRCPYAGDQRVTEAPNLGLNHLLFVREHNRLANLLKELNPYWNNEKVYQETRRIIIAQVQHITYNHFLPSVVLPHTMLAYHLYSLSTGYSNVYDDKIDMSIRNGFGTAPLRYGHSQVMATISELKRDFQTLTEHKMEDNLMSPHLYQRNHGMGVVEDMARWLVTKPALKIDHEFVDSIRDKLFYLSQPPGNDLVSLNIQRGRDQGTPSYNKWREFCGLRNYTTFSEFGYLGYKLARLYNTVDDIDFYVGGLLEKGEKGSVGPTFSCVIGLQFYLLKIGDRFWYESGDKDFAFSLDQLNSIKGYTTLAKMFCINFGLKKIQKDVFTIPSEDNPLVDCDDIPDID

>Unigene10454_2A 129 539 minus strand C-type lectin B [Chlamys farreri]

MCVLPWKRIGSNCYLIRTETVSPDTAFALCARRNAYLANLETLEEALLLQYELKQNKSGRHFIVGGRNINRYKPGGDWRWVKKGSMPTMAYFAFGAGQPDGSNAAPQDCLFLFASDNYQLHDVFCYDTSYNGGYVCE

>Unigene11270_2A 77 778 adenylate kinase 2, mitochondrial-like [Strongylocentrotus purpuratus]

MPPVSSDSNEKGINAVLLGPPGAGKGTQAPWMAEKYCVCHLSTGDMLRAVVASGSELGKRCKDVMDKGQLVSDDLVVELIDDNLDKPMCRNGFLLDGFPRTVGQAEALDRLLEKRQTKLDACVEFAIDDSLLVRRITGRLFHSKSGRSYHVEFNPPKFPMKDDVTGEPLIRRSDDNEEALKKRLESYHKQTKPLVDYYSKKGIHTAIDAAKPPLEVFAAIKKIFAPASSDDSKK

>Unigene11462_2A 45 686 minus strand apextrin-like protein [Mytilus galloprovincialis]

LPLIMMVSYAYAVQWPKGTYTLVKPKVGCPPGWKEGWRKQDNEDKNNQNFITPGHHFFGSFGRNMVFHYCTKDEHVITGHHNWPSGNYCILRSGISCPPDFQTGSIHWDDEDKRNENSYGGVLPSGSYDRNTLINYCCRSDGSASTPIELPTNKPFYLLRYKAPCQQVKGRLVRQEIVKSDDEDSNNKNSVSGSHPLDDGGRNHRLFYCYYYUY

>Unigene22870_2A 228 758 Tumor protein p53-inducible protein 13 [Harpegnathos saltator]

HCDDGHTKLDKDFTCAEFMCNGRQIKGAVKNIIVKKHMKEKDYGNDHACMDKTLSYNVTIPSSGTHRPAWPKYGEYPYVPPQRWIHSLEHGAIVFLYNPCLERSQVELLWKETQRMARKRKHLFIMTRYNLPADMPIALVAWRANVQLNGVDSAAIREFIKAKYNQGLEKVSKDGQY

>Unigene22875_2A 740 1582 foot protein-4 variant-1 [Mytilus californianus]

YNGDAYTLNVNRNKFYRYWYRYYKKYHDVDEIFNVLNRQHFIVKYGGKTGINYVDYYNTHDGFELDDNRFVYDGKTYLLKIARPRFYQLWYQYYNQYHDVDQVFNTLYGRRYFVLDNGDVVAGTDFGDLGNYLDQHRVIANHHVSRINTVASSHVSRISHISHSHVRTINREGNRHVIRIRGEANRHINKLRQIVNTHIRRLENKRRRHLTDIERQHRRHMQQREIQNRNHIANKQAVNTRHETFIARAAAHFSDIDYLNTYHGIKIINNRITYGGKAYVL

>Unigene22934_2A 46 489 minus strand lysozyme [Cyclina sinensis]

ILFIVISTLLILHKCQGIKKTKCEVVQALRARGVPDYDLRDWLCLVEHESRFHYDATYLNDDGYTDYGLFQISEKFNCGRVGGTDSSTCWRLRTYGCADSCQSLIDSNITNDVDCAVRIKNCDGFKRWNAHTKYCSDVSGPEYDYSGC

>Unigene23015_2A 3 1634 78kDa glucose regulated protein [Crassostrea gigas]

IKHYPFKVVNKNSKPHIQVNVGDAAKSFAPEEISAMVLGKMRDIAEGFLGKKVTNAVVTVPAYFNDAQRQATKDAGVIAGLNVMRIINEPTAAAIAYGLDKKEGEKNILVFDLGGGTFDVSLLTIDNGVFEVVSTNGDTHLGGEDFDQRVMEHFIKLYKKKKGKDIRKDNRAVQKLRREVEKAKRALSSAHQARIEIESLFEGEDFSETLTRARFEELNMDLFRSTMKPVQKVLEDADMKKEEIDEIVLVGGSTRIPKIQQLVKDQFNGKEPNRGVNPDEAVAYGAAVQAGVLGGEEDTGDLLLLDVNPLTMGIETVGGVMTKLIPRNTVIPTKKSQVFSTAADNQPTVTIQVFEGERPMTKDNHLLGKFDLTGIPPAPRGVPQIEVTFEIDVNGILKVSAEDKGTGNKNNIVIQNDNNRLSPEDIERMINDAEKFADEDKAVKEKVEAKNELESFTYNLKNQIGDKEKLGGKLSDDDKSTIETAVDEKIKWLDSNPDATTEELKEQKKELEEKVNPIISKIYQGAGGAPPPGGEEEGSEKDEL

>Unigene23092_2A 1157 2902 minus strand RecName: Full=DNA-directed RNA polymerase subunit beta''; AltName: Full=PEP; AltName: Full=Plastid-encoded RNA polymerase subunit beta''; Short=RNA polymerase subunit beta'' >gi|1171589|emb|CAA64574.1| rpoD [Plasmodium falciparum]

NRGFTLTGNTFVFNGRTYFLKCGISNFYRLWHRYYLRYESVNRIFRVLYRKRVFVKIDYLAYYRKHHGFIIKGKRFVYNGRAYVLKCGISRFYRLWYLFYERYQSITKIFRVMYSKRLFIRINYVDYYKSHRGIKLKGKILFYDGRIYVLRCTRNKLYRLWYRYFTRYQSVTKIFSVLFKKKVVVRINFATYYRTHLGLSLKGKTFVYSGHAYELKCGIKRFYRLWHKYYLQYDSVSKIFRVLFRKRVFVRMNYVNYYRKHRGFIIRGKSFVYNGKSYVLRCGLSRFYRLWYRYYERYQCISKIFTKMYSKRLFISVNYVAFFKTHHGFKLRGKLFIYNGKTYILKCSSSRLYRLWYRYYIRYQSVTKVFTALYRKHVFVKVNYLTFYKTHLGFTLNGRRFVYNGNAYVLRCAKHMFYRLWYQNYLRYQSVTRVFRVLYQKRIFVKVNNRINYLVFYQTHRGFTLKGSRFVFNGSEYVLKCKRNRFYRLWYRYYQRYHSIAKVFKLLHHKHLFVKFNYLFYYKKHHGFTLNGKRFVFNGRAYILKLGRSRLYRLWYRYYMKYRSMNKVFRILYKRRFIVRGT

>Unigene23176_2A 95 355 minus strand RecName: Full=Perlwapin-like protein; Flags: Precursor

CPDTSGIASACVIDPELHCFGDHQCRHGYKCCSYGCNRRCLAVNYIPIIHLGSCRNAGPLVNPKTCIIDSVCKSNEKCCSFRCAVVR

>Unigene23286_2A 139 444 Whey acidic protein precursor [Salmo salar]

FTDGSMLLPPQISKSGKCPVNMEPNVICDSDIDIDHCHHDGNCPGKEKCCRQGCFKVCQYPKFPEPVLIRCDSGKIFTGTFCNSNGKVCINGQTCETDSSGH

>Unigene23306_2A 263 682 minus strand Cu/Zn superoxide dismutase [Araneus ventricosus]

VYGNINLFQEFDSNIVRVWGRIRGLKPGLHGFHVHENGNFGWNCSNARGHFNPFTTNHGAPFDPPSDRHVGDLGNIRANYFGRARVDIHDGQISLVKQPHLSILGRAIVVHADRDDLGRGNNAGSRTTGNAGSRVACCQI

>Unigene23532_2A 39 629 AGAP003319-PA [Anopheles gambiae str. PEST]

KMNARLRIIAVHVCLVSWIYQTTDSCSCFPTHPQNQICRDGFVMVAKIVSRKEAPGSAGPFPVGQIEYTVRVIIPVKNSPTHGSTFKFTTADNGAACGVSYPIGSIQLLMGNNNRIGLCDSVFAKSLPQLSYIFSRKKDTSYLKNCDCKIDDGGVSPKLSAKQTCKTPSNQNTKACYANQGLCVKVGRGRRSGCVWK

>Unigene23798_2A 218 4483 minus strand thioester-containing protein-E [Chlamys farreri]

MTALLLTPVVLAACKNTYMVTVPQSIRPGHEFSVCVVPLVRHLGFASMQFTLMNRKNKTITYADKDILLHSDPVVIKIKVPQNVSVGYGYQLDVKSMGGLSFAKRVTDMRMLSKTSSIFVQTDKAAYKPGDLVQFRVFGTNSRLRFLNDPINVILTDPKRNRVDQYLNVKPSYGVFTGSFQLSTLTNLGSWNIKVEQKQEKYTKSFTVEKYVLPKFQINVVFPPIVLRTDKYFTVDLDAQYTFGKPVEGNVNLTISRTWSNRNVINKRFKMRGKTTIRVLMSEILRLGSYIKVKAILSERLTGKKASDTKTIYIRSYPERLIFSDSISSNFKPGLDYTMIIRAAKVDGTLLDKPLGTVNITVNYSIRKKIPEEFETQVKRIYPGATESKVLWSRSVQIPESGIIMISARFPIAAVSGYINLKYRKAYGSKYLSRAYSPSNSFIQVSIANDAKAKPGRPLPLILETTDRRRCVHYKVFSKGTLVTQGKWHCWSKKQRVNLMITTKMAPSAKLLVYYNRGHNGEIVADVISFPIKDVFDNEVSIKFNKKKALPGEKVELQIKADAKSLVNILSVDKSVLLYKTGNDITVQDVMRELQQYDSRYYPFSWGWNFWFPRRLSGVDAFSIFEQAGVYVLTDALLYQYLSECSRGSGGGRRMFLFKGGSGSVSASSSSGKSGTLATPTRIRKNFPATWIWANTVTGPDGNAVYTATAPDTVTTWVTSAFAVNPTSGLGLASELANLTTFQNFFMRFDLPYSVIRGEVVIVQIVVFNYLPRDQEVSVNLASSTDFSFVDTDGKTINQGSKGYSKTVNVKSNRIAGVYIPIIPTKVGAIKLNGQARSTEAADAVEHKLIVEAEGIQQSYNIPFLIDLRQSNSFSGSISISFPPNFVHDSEFIKIQVIGDLMGPTLNNIENLLRMSYGCGEQNLVRFVPNVYVSSYLKATNRLTKLLLRKAQGYLKAGYQRQLTYSRNDGSFSGYGNNDLRGSTWLTAYVIKAFSQAAEFTFIDKNVITKAIQWLLKQQASEGDFREPGIVFSKSMQGGSASSRRSLTAFVLISLIEAKIKRQVDSKIIGTVNSAITNATKFVADGAPFSIRNVYELAISFYALSLAKHSTAPSLLTELENKANTTSGEKFWTLPDADIYKISNWRSWRPPKTRARAIDIETTSYVLLGYNLRKDVNNGIRVLKWLGRQRNPWGGFISTQDTVIALQGLTHLGKKLYSTSTSVTITAKGDTWSGKTFNVNNANALVLQSEDLPKSVRKIEIIARGRGICLLETAVFFNVFKEMRQPAFELKPTVLRDSTRGFVLKMCFRWLRAGQSTMGYLEITLPSGMEADLGSLNTKQTFGQFKKIEKAFRQINLYFDAILSNEMCIEINVDRVSMIARHKPVPCRLSEYYEPSNEVIVMYQSKALANATIIEVCGAGNC

>Unigene23909_2A 176 547 minus strand PREDICTED: c-type lectin domain family 17, member A-like [Equus caballus]

PRVFDPNAKTLSYAGKNYIFSAIKLNFMAAQAACKSACGTLVAINSATEQNFINANTGSTEAWYIGLARVRGMWRWLSGEPVTYMNWFTSNPVGRIYCASLTQRKQWFAYQLPTCSIAQRFICE

>Unigene24351_2A 55 1359 minus strand adenosylhomocysteinase [Danio rerio] >gi|28278417|gb|AAH44200.1| S-adenosylhomocysteine hydrolase [Danio rerio] >gi|37681725|gb|AAQ97740.1| S-adenosylhomocysteine hydrolase [Danio rerio] >gi|182890144|gb|AAI64526.1| Ahcy protein [Danio rerio]

MSTKPPFKVADINLADWGRKCIEIAENEMPGLMQMRKMYGETKPLKGARVAGCLHMTTQTAVLIETLTALGAQVQWSSCNIFSTQDFAAAAIAKTGVPVYAWKGETDEEYIWCIEQTLVFPDGQPLNMILDDGGDLTNLVHERFPQYLPGIVGLSEETTTGVHNLHKMMKDGKLKIPAINVNDSVTKSKFDNLYGCRESLVDGIKRATDVMLAGKVAMVAGYGDVGKGCAHALRAFGARVMVVEIDPIIALQAAMEGFEVTTVEECLPKCRLFVTATGCSSIIHDKMFEQMLEDSIVCNIGHFDCELDVKWLNENCAKKEQIKPQVDRYTLKNGRHVILLAEGRLVNLGCAHGHPSFVMSNSFTNQVLAQIELWTKKEEYKNKISVTVLPKKLDEAVAAAHLDHLGVKLTKLTEEQSSYLGIPRDGPFKPEIYRY

>Unigene24763_2A 79 1725 mitochondrial H+ ATPase a subunit [Pinctada fucata]

MLSARFAATLVRQLPRAAPKVCRHALGAGYVASRNISTSTPLCAGAEVSSILEERILGQTSQTNLEETGRVLSIGDGIARVYGLKNIQAEEMVEFSSGLKGMALNLERDNVGVVVFGNDKLIKEGDIVKRTGAIVDVPVGKEMLGRVVDALGIPIDGKGPLGTSTRARVGVKAPGIIPRISVKEPMQTGIKAVDSLVPIGRGQRELIIGDRQTGKTAIAIDTIINQKRFNDGTDEKAKLYCIYVAIGQKRSTVAQIVKRLTDADAMKYTVIVSATASDAAPLQYLAPYSGCAMGEYFRDNGMHAVIIYDDLSKQAVAYRQMSLLLRRPPGREAYPGDVFYLHSRLLERAAKMNDDNGGGSLTALPVIETQAGDVSAYIPTNVISITDGQIFLETELFFKGIRPAINVGLSVSRVGSAAQTKAMKQVAGSMKLELAQYREVAAFAQFGSDLDQATQNLLNRGVRLTELLKQGQYIPMPIEEQVAIIYAGVRGHLDKLDPTKITDFEEAFLQHIRGSQKDLLATIAKDGMITEDSDAKLKQVVKNFLAGFE

>Unigene24907_2A 52 3738 minus strand thioester-containing protein [Chlamys farreri]

ILKCMATSCNHFITVPKSVTAGSELDISITVMKKGSQSMTMLAILKNSASKGISGASRNVRGKSGKTETLKIKIPDDTPPGSSYQVEISVSGWIFLHKTTYNVVIQKEKSIILIETDRPIYKPGDLVQFRTFGIDTDLKPLDNPLNITIFDSSGNRVKQYTNVMDKFGMFGDSLQLSMVTNLGNWKIDVRQASHRDSVSFEVAEYELPKFEVKVVLAGIPHIMDDFFKIRVTAIYTFGKPVEGYATIKIKRRWSSTDILVHKTIKILGKVDIKISTWDLYPVYGTYFDVNAVVNETVTGKNDTDFMSFDLKSSKWRVTFSPTMTLMKPGLKYDILFRIEDINGKPVKSKGIAKLIVGYDYTIPYKRSTLRKKGILVQKNLKLGPTGEVDYGVLFPPNTTSARIYVIYEKDNIGNKYIGKATSPSENFIQIRTKGIHTVQVDHLLTLILSVTENIRLCNYKILSSGVTVKEGIFRMNRRKTRKQKVYITRDMIPAARILVYYIRHDGEIVADAITFPVSDIFENKVTISFDKDKAEPGQRVNLRLTADPGSLLNVAAVDKSILLLKDANDIDADEVLSEVTSGDFFTQRLSKVWGRTVTTPETGSTANTVFQNSQLNVITDCELVGEITRQPFRGHPGAARTIGTTHTGLLSTLRTSVAATEALQSSLSPTAPVEENLVTPSRKRSNFQETWLWMNTTASSNGVSTLGVVVPDTITQWIASSYAINPLTGLGISLEKANITTFQRFFIRIELPYSVIRGETLILKISVFNYMETDQMVHVSMKKNENLTFVDENGNTIPVQGDSRLKLVNVPKDSIRSVYFPIIPTRIGKTSLDVTAQTTEVADAVKRNLLVKPEGISQKYNLPVAIDLREERRFSTSVKITFPSNVIPDSEFIKVSATGDLLGSTLDGIEDLLKMSYGCGEQNMLNFVPNVFVSEYLNVTNRFDKEIEKKVKKILADGYQRQLSYSRYDGSYSAFGKSDRRGSTWLTAFVLKSFAQAAKFTYIDPNVMQRATQFLINQQRSGQFTESGRVLHKAMQGGSAASVASLTAYAVIALSETLRHQQIPKHMVVRTVGAIRSGVDFIANSLVAKKNTTREMYELVICTYALTLQKALLANTLVKDIEKMANYSDGQMFWVLPDNEVDRLQPYTFWRPPSKQYRALDIEVTAYMLLIYNIRNEISKGVQVMRWLNDRRNPYGGFVSTQVLSINTETCLPTLQURSRYGTTPNISL

>Unigene24922_2A 26 1588 minus strand ATP synthase beta subunit [Pinctada fucata]

MMHAARRACVGLLKATKHSLTSPAVPSTATKALPSYFNTRHYAAEQTQPNTAKGRVVSVIGAVVDVQFDEELPPILNALSVENRTPKLILEVAQHLGENTVRTIAMDGTEGLVRGTSCIDTGYPIRIPVGPATLGRIINVVGDPIDERGPVKTDKFLSIHAEAPDFVEMSVTQEVLETGIKVVDLLAPYAKGGKIGLFGGAGVGKTVLIMELINNVAKAHGGYSVFAGVGERTREGNDLYHEMITSKVISLTDDTSKVSLVYGQMNEPPGARARVALTGLTVAEYFRDQEGQDVLLFIDNIFRFTQAGSEVSALLGRIPSAVGYQPTLATDMGTMQERITTTKKGSITSVQAIYVPADDLTDPAPATTFAHLDATTVLSRGISELGIYPAVDPLDSNSRILDPFVVGEEHYDVARNVQKILQNYKSLQDIIAILGMDELSEEDKLTVARARKIQRFLSQPFQVAEVFTGSEGKYVPLKESIAGFQRILSGELDHLPEVAFYMVGPIEEAVAKAERLAEDQS

>Unigene25134_2A 128 409 minus strand PREDICTED: WAP four-disulfide core domain 2-like [Saccoglossus kowalevskii]

EKPGQCPVTRGITPCVCIPENFECRFDSNCPGAMKCCDFGCGCNKRCVPPVPSPLQCYYNGQYYPIGAHFPSVDGCNTCYCNDDGTVMCTLKAC

>Unigene25176_2A 2 691 minus strand thioester-containing protein [Euphaedusa tau]

RQRNPWGGFISTQDTVIALQALAYLGKELYSPSSSITVTTKGNTWTGKTFIVNNVNALVLQSEDIPKSVRQIQIEARGNGICLLEAAVFFNVYKELREPTFELTPTVIRDSTRGFTLKVCFRWLRGGQSTMGYLEIAIPSGTEADMKSLNTKGAAGQFKKIEKAFRQVNLYFDAILSTTMCIEINIDRVSMVARHKPVFVRLSEYYEPSNEIIAFYQSKALANATIIEVC

>Unigene25183_2A 608 4369 thioester-containing protein [Chlamys farreri]

KLFSVERYVLPRFDVNVIMPSFQLTTDTFFRVKISAKYTFGKPVKGTCNLTIQKPWTTSGAINKRFKINGLASVKVLMSDVFKLGYNIRVTAVVTEGITGISANDTSKTILYRTPELLKFSSSMPRIFKPRLTYTIILRVTKRDESPLEPPLGKIKLTVKYYEKLKSNIPNEKGTKISTGEKGERGPDGSIGARGPRIDISWRPQPRLNERILLTKYVQIPSNGIIKEAVEFPLSAERATVQASYRKGNAYRYLSRARSPSNSYLQVSIVNSRKPTPNRYLILNIRTTESNVRYAFYKVISKGSIVTKGRFRLYRRSSIVRLKIKYTMAPSAKLLFYYTRRSGEIVADAITILVDDIFKNRVGIMFNKRKALPGDKVRLDIQAGANSMVCVLAVDKSVLLFRGGNDISVQDVMKELLQYDTSMHVPIFRRWDFWYPRSFSGIDAYSVFYLFGVYVLTDANLYKYRPPPRHLLPPTRFGGEKHGMAGQDGGSAPDMAFVDQNTPINPDLVTPTRTRKHFPETWLWKNAFTGLSGRTQISSIAPDTITEWVTSAFAVSPTTGLGVASEFANLTTFQRFFMRFDLPYSAIRGEQIIVQISVFNYLATEQRVVVTLSNSVDFIFVDYNGNALPTGSNGISKTIIVKTNSVGAVYIPIEATAIGKIKLHATARCEAAADAVEKDFLIEAEGIKQSYNMPLIIDLKTTSIFNAVIDIDFPPNLVGDSEFIKLQVIGDLLGSTLNGIEDLLRMSYGCGEQNMIGFVANVYVSTYLKATERLNLDLEAKAKKVIAAGYQRQLTYAHNDGSYSAFGNNDKSGSTWLTAYVIKSFSQASKFVYIDKDHVMKRALVWITKQQEHNGTFDEPGKVLHKAMQGGSVSSKRSLASFVLIALEEAKRNNQVSKSILSSVNLAILKAAKFIAHDAPASIGNLYELAISFYALSLVNHSKKDELLIDLENKAKTDGGGKYWKLTGPVAQKVQPWRRWNPPRTNARAIDVEITSYVLLGYNVKGDTLNGIKVLRWLGNQRNPRGGFISTQDTVIALQALSGLGVKLYVKTFSMTVKTKGDIWSGKAFKINDKNALVLQSEDLPKSVRSISISAHGTGLCLLEAAVYYNVYDELRIPAFDLIPTVLKETINGFTLKVCFRWKRGGESTMSLLEINIPSGMEADTDSVDASEAHGQYTTIEKAFRKVNLFFDAILSIQMCIRIDIRRTSMVARHKPQSIRLSEYYEPSNEVIKTYISNKLANATIIEVCGSDNC

>Unigene26037_2A 200 451 pleiotrophin-like protein [Patella caerulea]

GEWSECDSSNQMKRTLTLREQNDGCPATKEQTRPCKDKKSKGCKYRADGDWSECDTALNIRTRKVVLHKGDSHCALEKTNEISC

>Unigene38666_2A 295 633 minus strand hypothetical protein BRAFLDRAFT_84494 [Branchiostoma floridae] >gi|229299135|gb|EEN69755.1| hypothetical protein BRAFLDRAFT_84494 [Branchiostoma floridae]

YNYFDQIAQTYCQGRFVPGWVFAVRRDCRCVAPTCNDICATAKSDILATIEFQRNDIACFDGISIKKDHVKLADNPTGSEPDAGKVSLSTYGYGDGGCTWTPNHCGPNYCCCK

>Unigene58046_2A 196 729 PREDICTED: catechol 1,2-dioxygenase-like, partial [Xenopus (Silurana) tropicalis]

TSSDVIGPFYFPDPPKLKGFCRYERTIKDDEDEVPLYVYGRVKSYDCKTPLRGVRVEVWQADHTGSYRNTSKCRGYIKTDKYGVYQFVTIYPGRYTASHYGDDYRPAHIHFKVFGPDGHKTLVTQMYFHDDIYLGEKDPCVGCSSHRNDLIVKKDRYCDFDNMKKDYCIDTVEFDINL

>Unigene63380_2A 81 332 hypothetical protein [Latrodectus hesperus]

MFMCACDAMIHCMPGICDNYACEPVSESTCAGEFVKSGGVCGCCDLCITVLDEGDDCSKLMMAGPVQDHKCRAGLWCDWDTLRC

>Unigene64269_2A 397 1311 PREDICTED: peptidylglycine alpha-amidating monooxygenase-like [Saccoglossus kowalevskii]

PNWPLGNLKLGQVAGVAVDGNGDVLVFHRADRRWGAGTFDFNNRLSNNEQSKGPIRNATILRINTKGTVVKELGKDQFFMPHGLTIDGKGNIWVTDVGLHQVFKLPSKPDETPLILGEAMVPGSDNDHFCKPTDVAVASTGHFFVADGYCNGRVMKFEKNGHLIKEWGKQSREPVSLAGNDEMFIPHSIALIEESDLVAVADREHGRIQIFTAGITDPNDTGRFVKSISDPRFGKVFAISYDQTDKRLYVVNGQSGRNRVAGFTVDLNGQIHDTWEPNSEDFDEPHDVAVSPDGHHVYVGEIRPN

>CL606.Contig1_2A 62 1819 minus strand PREDICTED: similar to senataxin [Hydra magnipapillata]

LIGLEITVKTRLRQFKMLSSKRSTITSVTSLVSTVRQYIALSHVFRTTIINHILSPSSLRVFHNHPCVKEQDREGLENYNPSQQKAITIASKIAIEPPVQNRIVLLQGPPGTGKSFTIVGIIKKIFERSNGRCKICLCAPSNSAVDELIKRLITERRKMIEEKKNPFGLVRIGKQENMHQDVLKYTFQEILKSNLDVELKRKMRAKVGSSEFKELNTLTSRIQRLQLDMEEKTVRLQNIQAGKLMSEITKLQKKKDDMEAEIQQLNKDVKLTRDEEENVKKNILIKSHIVCGTLSSFGHHQYMNILAPLQRENQIRRSYFNCIIVDEASQATELDTLIPLQYGTSKMILVGDPEQLPPTVLSQKCALKSFGQSLFERFFNHFKHEEVNPVLFLDTQYRMHPDIAYFPSKFVYLGALKTDESVKGRCNKLQLRPYVVFDMQEGQEVTTRRGAIINSIEAEFTIELCEFLIKRGSLQQKEIGIIAPYQQQKKIIMEGLKSSQLDHIEVNTVDGFQGREKEVIILSCTRAKNSSGGIGFLANSKRMNVALTRGKSALYVIGCLDSLKKGDSAWKTLIEDAESRHVITKV

>CL40.Contig1_2A 78 1205 actin [Loligo bleekeri]

MAEDDVAALVVDNGSGMCKAGFAGDDAPRAVFPSIVGRPRHQGVMVGMGQKDSYVGDEAQSKRGILTLKYPIEHGIVTNWDDMEKIWHHTFYNELRVAPEEHPVLLTEAPLNPKANREKMTQIMFETFNSPAMYVAIQAVLSLYASGRTTGIVLDSGDGVTHTVPIYEGYALPHAIMRLDLAGRDLTDYLMKILTERGYSFTTTAEREIVRDIKEKLCYVALDFEQEMATAASSSSLEKSYELPDGQVITIGNERFRCPESLFQPSFLGMENAGIHETTYNSIMKCDVDIRKDLYANIVMSGGTTMFPGIADRMQKEVTALAPQTMKIKVIAPPERKYSVWIGGSILASLSTFQQMWISKQEYDESGPSIVHRKCF

>Unigene23040_2A 1 1657 LEN=1742; minus strand; translated

XRHYYDINKCFGMLYGQKLFHVYVSASAHAHAQSSAKGGNAVSSARAHARAHAVIVFPWKIYNNRFTYNRKVYQLDCTQKNLYNQWNLCYRHYYNRDKCFGMLYGQKLFHVYVSASAHAHAKSSAQGGRAVSSASAHAHAVIAFPWPIYHNRFTYNRKVYQLDCTQKNLYYKWNLCYRHYYDINKCFGMLYGQKLFHVYVSASAHAHAQSSAKGGNAVSSARAHARAHAVIVFPWKIYNNRFTYNRKVYQLDCTQKNLYNQWNLCYRHYYNRDKCFGMLYGQKLFHVYVSASAHAHAKSSAQGGRAVSSASAHAHAVIAFPWPIYHNRFTYNRKVYQLDCTQKNLYYKWNLCYRHYYNKDRCFGMLYGQKLFHVYVSASAHAHAFARSFSRGGHAVSSAHAKAQASARVIVFPWPIYNNRFNYNHKVYQLDCKQKELYNHWNNCYRLHHNQHQCFGLLLNHKFFHVYTAASASAHAVAHASGNGHAFASAKAHAQAYFTIVHKRFTYNGLGYRLGCDRNKFYNLYDQCYARLHSCTKCLNYLIAHKLLYKTTG

>Unigene25190_2A 1 766 LEN=766; translated

XDISITIFSMKFFIILCTAFLLGSEVSAFCSWPCTLQGKTFFNDASNARATFPNAYELVVNVGGVTSDILCLERNGQYVVNRRVGENGIFLYRCITWHNVGEGGFVIFQSDLVESDKPPEFCDICSPERVSSEPEVWVDLETTIMSCNVPKNCQSSSSSVQPCTGCEADTSDDGICCNQSNNDNDKTENNVQVLIDGQATRNPAPVEEIDIDKIIDIGGAVTLNDIEYQYQPSYTVXLKCEVSYLLCFIEEYAKLA

>Unigene60974_2A 1 1842 LEN=1927; minus strand; translated

QNLISNCVEEMKILVVFAILLSQCTAQFGPTVLPQTAIVSRPPDSSGGGFPQPCLQVLAKGGDSPYPPTSVARIYSMFCPVINEFATCLELHLNESSVEYFVLLSKHFDKNLMVSRMQHLCSLIPSDATSLDTLKTNNSDVQACVANFNREMARPSQPLTLNILCRLLDEHVFCMRQYATPDVIPENVLEVYFAYTLIFTPGQCNPPIPSTFMPTINTPPAVSNDPGQTWLAQSVVARCAYETYGEVIKNIMAIYKTHTNQLKTVGQVLGQAMGSLCQNATRFSDCISQNVKQPYSSIDAGLSVIINLQNLGSSLIQICSTSMTQSELMCLFQSGSSMPVCGSLISASTKLLQRSRSSTTPTNSINQLLSYCPLLREGDTCARIQLQRCSPALAETFSAVEKQLLTTNCYYADASVINASPYSNRMAYCAAPVMAEAYQRFANRPWAFDDEHVTSFACKESSNLATCLSREVGISKNLVDKMTSKLIDFSSGPRLQYIFDTVCNNTEEILRQSRCMVNATNSFRVCKMEQSYKDLQARYIFNFVFNISGIDVNGVTAFCKPYKELALCNARQISTCSESLSRLAEDVVNELLRMDCGGSRPLTQYMNFNAASDH

>Unigene63426_2A 1 327 LEN=327; translated

STSNAQVAQREINELKRQGVRVFTVGTYRADESELLSLASSPQDYQHARSFNDLVKGNDALDKITNQIRALQKPSSIPITTPQRPTRPPTTTPRATTRAPVDDLQKHLI
